# Supplementary material for: Genome-Wide Investigation and Expression Analysis of the Nitraria sibirica Pall. CIPK Gene Family
Source: Int J Mol Sci. 2022 Sep 30;23(19):11599. doi: 10.3390/ijms231911599 (PMC9569540; doi:10.3390/ijms231911599)
Supplement: Supplementary file 1 [file ijms-23-11599-s001.zip › Figure captions.pdf]

Figure S1: Chromosome localization of *NsCIPKs*. Figure S2: Transmembrane structure analysis of *NsCIPK* gene family. Figure S3: Prediction Phylogenetic relationships between CIPKs from seven species. Figure S4: Multiple sequence alignment of *NsCIPK* gene family. Figure S5: Motif sequence distribution of *NsCIPK* gene family.
