# Supplementary material for: Genome-Wide Investigation and Expression Analysis of the Nitraria sibirica Pall. CIPK Gene Family
Source: Int J Mol Sci. 2022 Sep 30;23(19):11599. doi: 10.3390/ijms231911599 (PMC9569540; doi:10.3390/ijms231911599)
Supplement: Supplementary file 1 [file ijms-23-11599-s001.zip › Figure S2 Transmembrane structure analysis of NsCIPK gene family.pdf]

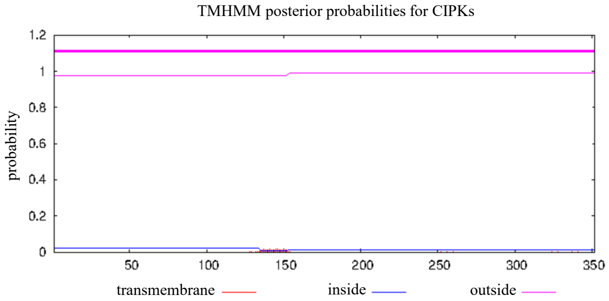

NsCIPK1-1

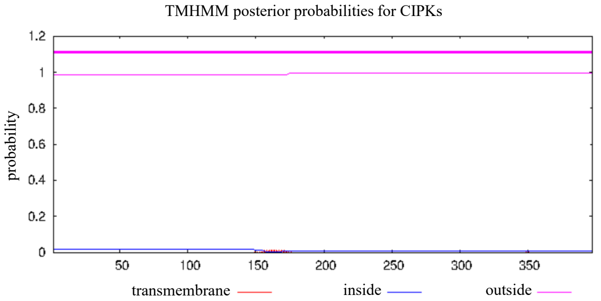

NsCIPK1-2

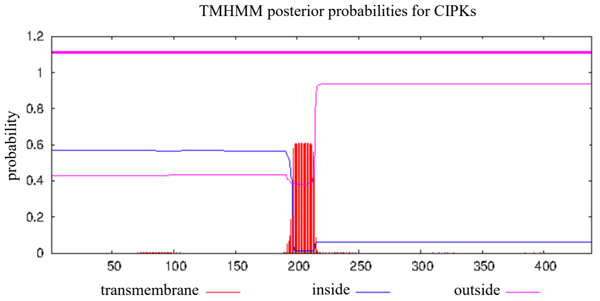

NsCIPK3-1

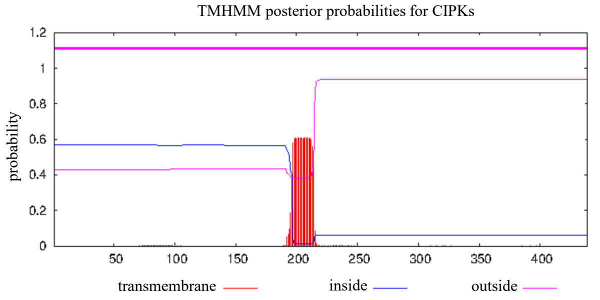

NsCIPK3-2

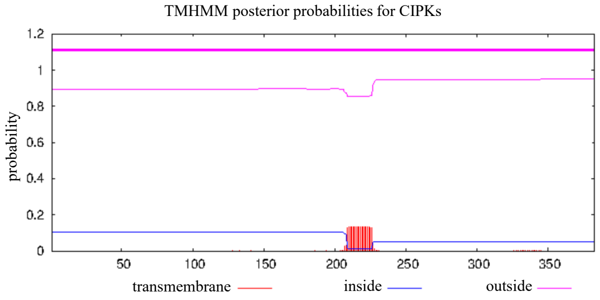

NsCIPK6

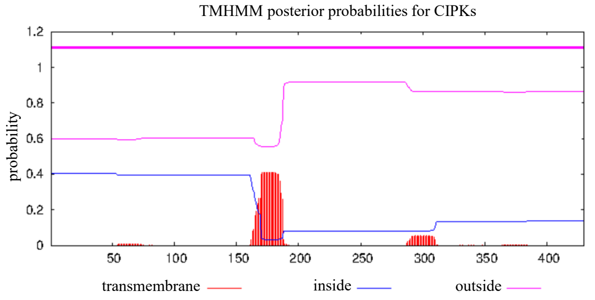

NsCIPK8

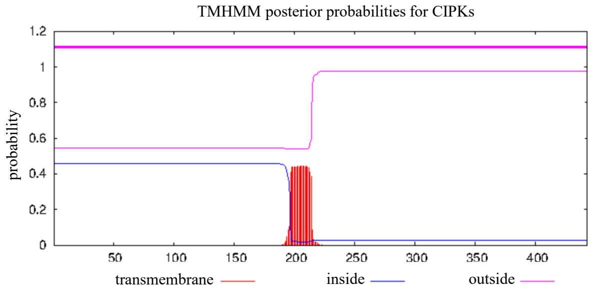

NsCIPK9

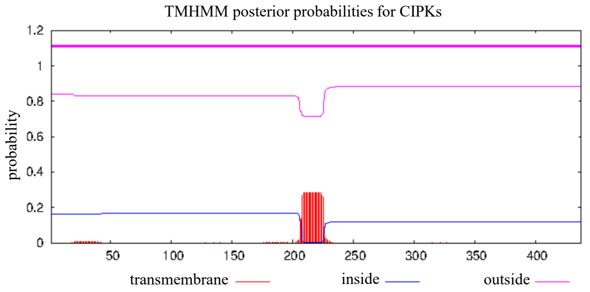

NsCIPK11

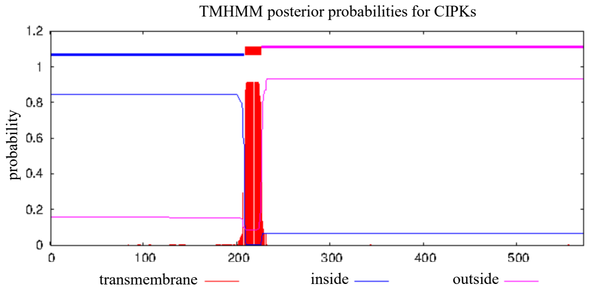

NsCIPK12

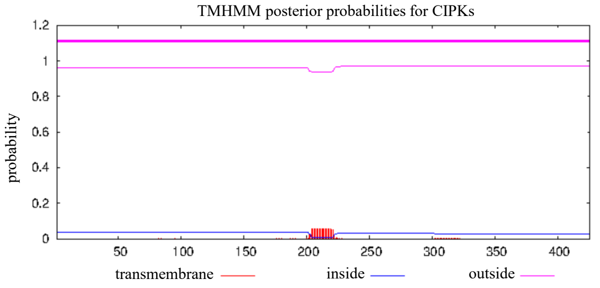

NsCIPK14

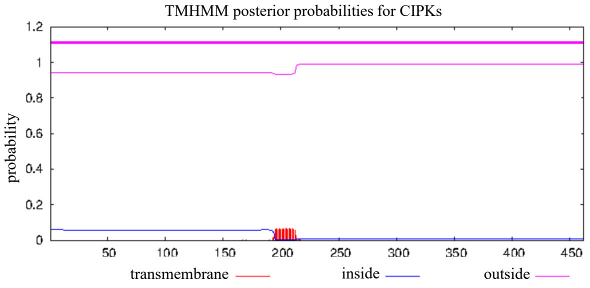

NsCIPK15

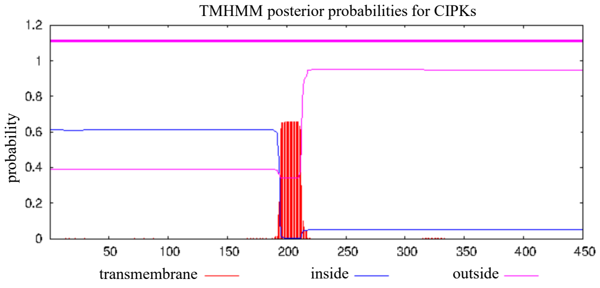

NsCIPK20

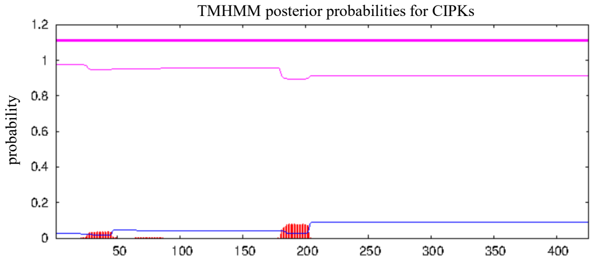

NsCIPK21

transmembrane inside outside

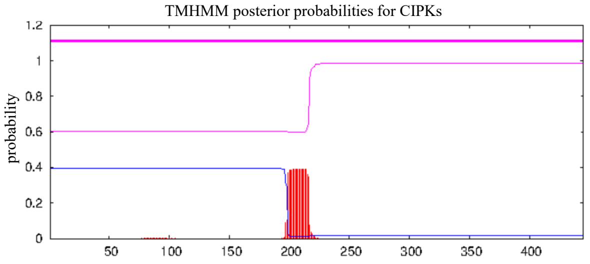

NsCIPK23

transmembrane inside outside
