# Supplementary material for: Genome-Wide Investigation and Expression Analysis of the Nitraria sibirica Pall. CIPK Gene Family
Source: Int J Mol Sci. 2022 Sep 30;23(19):11599. doi: 10.3390/ijms231911599 (PMC9569540; doi:10.3390/ijms231911599)
Supplement: Supplementary file 1 [file ijms-23-11599-s001.zip › Figure S4 Multiple sequence alignment of NsCIPK gene family.pdf]

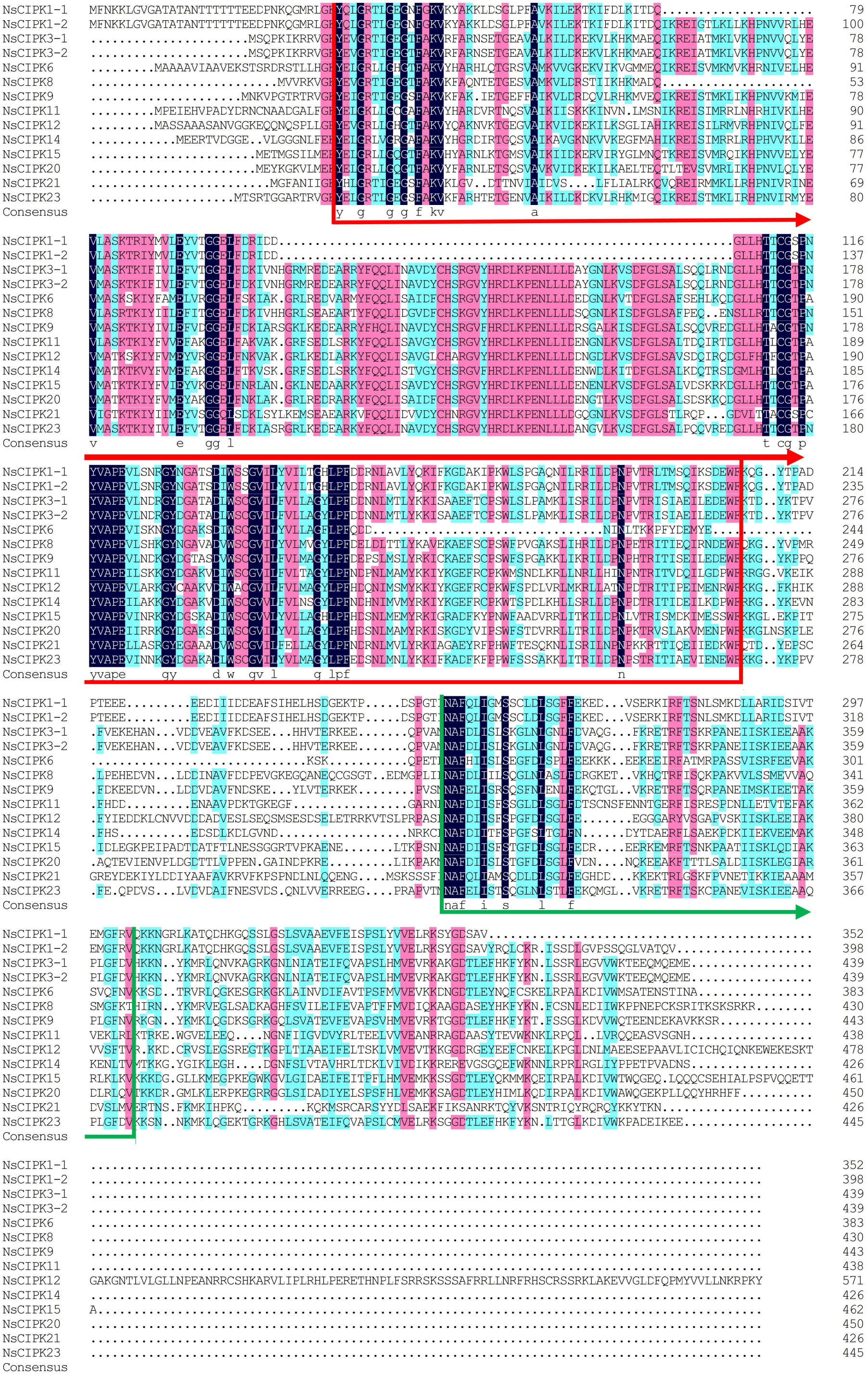

NsCIPK1-1 MFNKKLGVGATATANTTTTTTEEDPNKQGMRLGKYQLGRTLGEGNFGKVKYAKKLDSGLPFAVKILEKTKIFDLKITDQ.................... 79
NsCIPK1-2 MFNKKLGVGATATANTTTTTTEEDPNKQGMRLGKYQLGRTLGEGNFGKVKYAKKLDSGLPFAVKILEKTKIFDLKITDQIKREIGTLKLLKHPNVVRLHE 100
NsCIPK3-1 ......................MSQPKIKRRVGKYEVGRTIGEGTFAKVRFARNSETGEAVALKILDKEKVLKHKMAEQIKREIATMKLVKHPNVVQLYE 78
NsCIPK3-2 ......................MSQPKIKRRVGKYEVGRTIGEGTFAKVRFARNSETGEAVALKILDKEKVLKHKMAEQIKREIATMKLVKHPNVVQLYE 78
NsCIPK6 .........MAAAAVIAAVEKSTSRDRSTLLHGKYELGRLLGHGTFAKVYHARHLQTGRSVAMKVVGKEKVIKVGMMEQIKREISVMKMVKHRNIVELHE 91
NsCIPK8 ........................MVVRKVGKYEVGRTIGEGTFAKVKFAQNTETGESVAMKVLDRSTIIKHKMADQ.......................... 53
NsCIPK9 ...................MNKVPGTRTRVGKYEIGRTIGEGSFAKVKFAK.IETGEFFAIKVLDRDQVLRHKMVEQIKREISTMKLIKHPNVVKMIE 78
NsCIPK11 .........MPEIEHVPADYDRNCNAADGALFGKYELGKLLGCGAFAKVYHARDVRTNQSVAIKIISKKKINVN.LMSNIKREISIMRRLNHRHIVKLHE 90
NsCIPK12 .........MASSAAASANVGGKEQQNQSPLLGKYEVGRLLGHGTFAKVYQAKNVKTGEGVAIKVIDKEKILKSGLIAHIKREISILRMVRHPNIVQLFE 91
NsCIPK14 ....MEERTVDGGE..VLGGGNLFEKYELGRLVGRGAFAKVYHGRDIRTGQSVAIKAVGKNKVVKEGFMAHIKREISIMRRLRHPHVVKLLE 86
NsCIPK15 ....................METMGSILMEKYELGRLLGQGTFAKVHYARNLKTGMSVAIKIIDKERVIRYGLMNQTKREISVMRQIKHPNVVELYE 77
NsCIPK20 ....................MEYKGKVLMEKYELGRLLGQGTFAKVYFARNLETSQSVAVKVIDKEKILKAELTEQTLTEVSVMRLIKHPNVLQLYE 77
NsCIPK21 ........................MGFANIIGKYHLGRTIGEGSFAKVKLGV..DTTNVIAIDVS....LFLIALRKQVQREIRMMKLLRHPNIVRINE 69
NsCIPK23 ....................MTSRTGGARTRVGKYELGRTIGEGSFAKVKFARHTETGENVAIKILDKDKVLRHKMIGQIKREISTMKLIRHPNVIRMYE 80
Consensus y g g g f kv a

NsCIPK1-1 VLASKTRIYMVLEYVTGGELFDRIDD...GLLHTTCGSPN 116
NsCIPK1-2 VLASKTRIYMVLEYVTGGELFDRIDD...GLLHTTCGSPN 137
NsCIPK3-1 VMASKTKIFIVLEFVTGGELFDKIVNHGRMREDEARRYFQQLINAVDYCHSRGVYHRDLKPENLLLDAYGNLKVSDFGLSALSQQLRNDGLLHTTCGTPN 178
NsCIPK3-2 VMASKTKIFIVLEFVTGGELFDKIVNHGRMREDEARRYFQQLINAVDYCHSRGVYHRDLKPENLLLDAYGNLKVSDFGLSALSQQLRNDGLLHTTCGTPN 178
NsCIPK6 VMASKSKIYFAMELVRGGELFSKIAK.GRLREDVARMYFQQLISAIDFCHSRGVYHRDLKPENLLLDEDGNLKVTDFGLSAFSEHLKQDGLLHTTCGTPA 190
NsCIPK8 VLASRTKIYIILEFITGGELFDKIVHHGRLSEAEARTYFQQLIDGVDFCHSKGVYHRDLKPENLLLDSQGNLKISDFGLSAFPEQ..ENSLLRTTCGTPN 151
NsCIPK9 VMASKTKIYIVLEFVDGGELFDKIARSGKLREDEARRYFHQLINAVDYCHSRGVFHRDLKPENLLLDRSGALKISDFGLSALSQQVRDDGLLHTACGTPN 178
NsCIPK11 VLASKTKIYFVVEFAKGGELFAKVAK.GRFSEDLSRKYFQQLISAVGYCHSRGVYHRDLKPENLLIDENGNLKVSDFGLSALTDQIRTDGLLHTLCGTPA 189
NsCIPK12 VMATKSKIYFVMEYVRGGELFNKVAK.GRLKEDLARKYFQQLISAVGLCHARGVYHRDLKPENLLLDDNGDLKVSDFGLSAVSDQIRQDGLFHTFCGTPA 190
NsCIPK14 VMATKTKVYFVMEFAKGGELFTKVSK.GRFSEDLSRKYFQQLISTVGYCHSRGVFHRDLKPENLLLDENWDLKITDFGLSALKDQTRSDGMLHTLCGTPA 185
NsCIPK15 VMATKTKIYFVIEYVKGGELFNRLAN.GKLNEDAARKYFQQLISAVDYCHSRGVYHRDIKPENLLLDENENLKVSDFGLSALVDSKRKDGLLHTTCGTPA 176
NsCIPK20 VMATKTKIYFVMEYAKGGELFNKLAK.GRLREDRARKYFQQLISAIDFCHSRGVYHRDLKPENLLLDENGTLKVSDFGLSALSDSKKQDGLLHTTCGTPA 176
NsCIPK21 VIGTKTKIYIIMEYVSGGQLSDKLSYLKEMSEAEARKVFQQLIDVVDYCHNRGVYHRDLKPENLLLDGQGNLKVSDFGLSTLRQP...GDVLTTACGSPC 166
NsCIPK23 VMASKTKIYIVLEFVTGGELFDKIASRGRLKEDEARKYFQQLINAVDYCHSRGVYHRDLKPENLLLDANGVLKVSDFGLSALPQQVREDGLLHTTCGTPN 180
Consensus v e gg l t cg p

NsCIPK1-1 YVAPEVLSNRGYNGATSDIWSSGVILYVILTGHLPFDDRNLAVLYQKIFKGDAKIPKWLSPGAQNILRRILDPNPVTRLTMSQIKSDEWFKQG..YTPAD 214
NsCIPK1-2 YVAPEVLSNRGYNGATSDIWSSGVILYVILTGHLPFDDRNLAVLYQKIFKGDAKIPKWLSPGAQNILRRILDPNPVTRLTMSQIKSDEWFKQG..YTPAD 235
NsCIPK3-1 YVAPEVLNDRGYDGATADLWSCGVILFVLLAGYLPFDDNNLMTLYKKISAAEFTCPSWLSLPAMKLISRILDPNPVTRISIAEILEDEWFKTD..YKTPV 276
NsCIPK3-2 YVAPEVLNDRGYDGATADLWSCGVILFVLLAGYLPFDDNNLMTLYKKISAAEFTCPSWLSLPAMKLISRILDPNPVTRISIAEILEDEWFKTD..YKTPV 276
NsCIPK6 YVAPEVISKNGYDGAKSDIWSCGVILYVLLAGFLPFQDD.............................NINLTKKPFYDEMYE............ 244
NsCIPK8 YVAPEVLSHKGYNGAVADVWSCGVILYVLMVGYLPFDELDLTTLYKAVEKAEFSCPSWFPVGAKSLIHRILDPNPETRITIEQIRNDEWFQKG..YVPMR 249
NsCIPK9 YVAPEVLNDKGYDGTASDVWSCGVILFVLMAGYLPFDEPSLMSLYRKICKAEFSCPSWFSPGAKKLIKRILDPNPHTRITISEILEDEWFKKG..YKPPQ 276
NsCIPK11 YVAPEVLKNKGYDGAKVDIWSCGVILFVLTAGYLPFNDPNLMAMYRKIYKGEFRCPKWMSNDLKRLLNRLLHINPNTRITVDQILGDPWFRRGG.VKEIK 288
NsCIPK12 YVAPEVLARKGYCAAKVDIWSCGVILFVIMAGYLPFHDQNIMSMYKKIYKGEFRCPKWFSPDLVRLMKRLLATNPDTRITIPEIMENRWFKKG..FKHIK 288
NsCIPK14 YVAPEILAKKGYDGAKVDIWACGVVLFVINSGYLPFNDHNIMVMYRKIYKGEFRCPKWTSPDLKHLLSRLLDTNPDTRITIDEILKDPWFRKG..YKEVN 283
NsCIPK15 YVAPEVINRKGYDGSKADIWSCGVVLYVLLAGHLPFHDSNLMEMYRKIGRADFKYPNWFAADVRRLITKILDPNLVTRISMCKIMESSWFKKGL.EKPIT 275
NsCIPK20 YVAPEIIRRKGYDGAKSDIWSCGVILYVLLAGYLPFHDSNLMAMYRKISKGDYVIPSWFSTDVRRLLTRILDPNPKTRVSLAKVMENPWFKKGLNSKPLE 276
NsCIPK21 YVAPELLASRGYEGAAADVWSCGVILFELLAGYLPFNDRNLMVLYRKIAGAEYRFPHWFTESQKNLISRILNPNPKKRTTIQEIIEDKWFQTD..YEPSC 264
NsCIPK23 YVAPEVINNKGYDGAKADLWSCGVILYVIMAGYLPFEDSNLMNLYKKIFKADFKFPPWFSSSAKKLITRILDPNPSTRITIAEVIENEWFKKG..YKPPV 278
Consensus yvape gy d w gv l g lpf n

NsCIPK1-1 PTEEE........EEDIIIDDEAFSIHELHSDGEKTP.....DSPGTINAFQLIGMSSCLDLSGFFEKED...VSERKIRFTSNLSMKDLLARIDSIVT 297
NsCIPK1-2 PTEEE........EEDIIIDDEAFSIHELHSDGEKTP.....DSPGTINAFQLIGMSSCLDLSGFFEKED...VSERKIRFTSNLSMKDLLARIDSIVT 318
NsCIPK3-1 .FVEKEHAN...VDDVEAVFKDSEE...HHVTERKEE.......QPVAMNAFDLISLSKGLNLGNLFDVAQG...FKRETRFTSKRPANEIISKIEEAAK 359
NsCIPK3-2 .FVEKEHAN...VDDVEAVFKDSEE...HHVTERKEE.......QPVAMNAFDLISLSKGLNLGNLFDVAQG...FKRETRFTSKRPANEIISKIEEAAK 359
NsCIPK6 ..............................KSK...........QPETINAFHIISLSEGFDLSPIFEEKKK..EEKEEIRFATMRPASSVISRFEEVAK 301
NsCIPK8 .LPEHEDVN...LDDINAVFDDPEVGKEGQANEQCGSGT..EDMGPLIINAFDIIILSQGLNIASLFDRGKET..VKHQTRFISQKPAKVVLSSMEVVAQ 341
NsCIPK9 .FDKEEDVN...LDDVDAVFNDSKE...YLVTERKEK........PVSMNAFELISRSQSFNLENLFEKQTGL..VKRETRFTSQRPANEIMSKIEETAK 359
NsCIPK11 .FHDD.........ENAAVPDKTGKEGF.................GARNINAFDIISFSSGLDLSGLFDTSCNSFENNTGERFISRESPDNLLETVTEFAK 362
NsCIPK12 .FYIEDDKLCNVVDDADVESLSEQSMSESDSELETRRKVTSLPRPASINAFDIISFSPGFDLSGLFE.......EGGGARYVSGAPVSKIISKLEEIAK 380
NsCIPK14 .FHS..........EDSDLKDLGVND.................NRKCINAFDIITFSPGFSLTGLFN......DYTDAERFLSAEKPDKIIEKVEEMAK 348
NsCIPK15 .IDLEGKPEIPADTDATFTLNESSGGRTVPKAENE........LTKPSNINAFDIISFSTGFDLSGLFEDR.....EERKEMRFTSNKPAATIISKLQDIAK 363
NsCIPK20 .AQTEVIENVPLDGDTTLVPPEN.GAINDPKRE..........LIKPAKNNAFDIISLSTGFDLSGLFVDN....NQKEEAKFTTTLSALDIISKLEGIAR 361
NsCIPK21 GREYDEKIYLDDIYAAFAVKRVFKPSPNDLNLQQENG....MSKSSSFINAFQLIAMSQDLDLSGLFEGHDD...KKEKTRLGSKFPVNETIKKIEAAAM 357
NsCIPK23 .FE.QPDVS...LVDVDAIFNESVDS.QNLVVERREEG....PRAPVTNNAFELISTSQGLNLSTLFEKQMGL..VKRETRFTSKCPANEVISKIEEAAQ 366
Consensus naf i s l f

NsCIPK1-1 EMGFRVQKKNGRLKATQDHKGQSSLGSLSVAAEVFEISPSLYVVELRKSYGDSAV... 352
NsCIPK1-2 EMGFRVQKKNGRLKATQDHKGQSSLGSLSVAAEVFEISPSLYVVELRKSYGDSAVYRQLCKR.ISSDLGVPSSQGLVATQV.................... 398
NsCIPK3-1 PLGFDVHKKN..YKMRLQNVKAGRKGNLNIATEIFQVAPSLHMVEVRKAKGDTLEFHKFYKN.LSSRLEGVVWKTEEQMQEME................. 439
NsCIPK3-2 PLGFDVHKKN..YKMRLQNVKAGRKGNLNIATEIFQVAPSLHMVEVRKAKGDTLEFHKFYKN.LSSRLEGVVWKTEEQMQEME................. 439
NsCIPK6 SVQFNVKKSD..TRVRLQGKESGRKGKLAINVDIFAVTPSFMVVEVKKDNGDTLEYNQFCSKELRPALKDIVWMSATENSTINA................ 383
NsCIPK8 SMGFKTHIRN..YKMRVEGLSADKAGHFSVILEIFEVAPTFFMVDIQKAAGDASEYHKFYKN.FCSNLEDIIWKPPNEPCKSRITKSKSRKR........ 430
NsCIPK9 PLGFNVRKGN..YKMKLQGDKSGRKGQLSVATEVFEVAPSVHMVEVRKTGGDTLEFHKFYKT.FSSGLKDVVWQTEENDEKAVKKSR............ 443
NsCIPK11 VEKLRLKTRKE.WGVELEEQ....NGNFIIGVDVYRLTEELVVVEANRRAGDAASYTEVWKNKLRPQL..LVRQQEASVSGNH................ 438
NsCIPK12 VVSFTVR.KKD.CRVSLEGSREGTKGPLTIAAEIFELTSKLVMVEVTKKGGDRGEYEEFCNKELKPGLDNLMAEESEPAAVLICICHQIQNKEWEKESKT 478
NsCIPK14 KENLTVMTKKG.YGIKLEGH....DGNFSLVTAVHRLTDKLVIVDIKKREREVGSGQEFWKNNLRPRLRGLIYPPETPVADNS................ 426
NsCIPK15 RLKKLVKKKDG.GLLKMEGPKEGWKGVLGIDAEIFEITPTFLHMVEMKKSSGDTLEYQKMMKQEIRPALKDIVWTWQGEQ.LQQQCSEHIALPSPVQQETT 461
NsCIPK20 DLRLQVTKKDR.GMLKLERPKEGRRGGLSIDADIYELSPSFHLVEMKKSAGDTLEYHIMLKQDIRPALKDIVWAWQGEKPLLQQYHRHFF.......... 450
NsCIPK21 DVSLMVERTNS.FKMKIHPKQ.........KQKMSRCARSYYDLSAEKFIKSANRKTQYVKSNTRIQYRQRQYKKYTKN................ 426
NsCIPK23 PLGFDVKKSN..NKMKLQGEKTGRKGHLSVATEIFQVAPSLCMVELRKSGGDTLEFHKFYKN.LTTGLKDIVWKPADEIKEE................ 445
Consensus

NsCIPK1-1 352
NsCIPK1-2 398
NsCIPK3-1 439
NsCIPK3-2 439
NsCIPK6 383
NsCIPK8 430
NsCIPK9 443
NsCIPK11 438
NsCIPK12 GAKGNTLVLGLLNPEANRRCSHKARVLIPLRHLPERETHNPLFSRRSKSSSAFRRLLNRFRHSCRSSRKLAKEVVGLDFQPMYVVLLNKRPKY 571
NsCIPK14 426
NsCIPK15 A... 462
NsCIPK20 450
NsCIPK21 426
NsCIPK23 445
Consensus
