# Supplementary material for: Genome-Wide Investigation and Expression Analysis of the Nitraria sibirica Pall. CIPK Gene Family
Source: Int J Mol Sci. 2022 Sep 30;23(19):11599. doi: 10.3390/ijms231911599 (PMC9569540; doi:10.3390/ijms231911599)
Supplement: Supplementary file 1 [file ijms-23-11599-s001.zip › Supplyment File1.pdf]

Additional file S1. The genome DNA, mRNA and protein sequences of CIPKs of *N. sibirica*

1>NISI03G1369
ATGCCGGAGATTGAACATGTCCCCGCGGATTACGACCGCAATTGCAACGCTGCCGACGGT
GCCTTGTTTGGAAAGTATGAGCTCGGCAAGCTCCTCGGCTGCGGAGCCTTCGCTAAGGTG
TACCATGCGCGTGACGTCCGTACGAACCAGAGCGTGGCGATTAAGATCATTAGCAAGAAG
AAGATCAACGTTAATCTGATGTCGAACATCAAGCGTGAGATCTCGATCATGAGGCGGTTG
AACCATCGCCATATCGTGAAGCTCCACGAGGTTCTGGCGTCGAAAACGAAGATTTATTTC
GTCGTGGAGTTCGCCAAGGGCGGCGAGTTGTTCGCCAAGGTGGCGAAAGGAAGGTTCAGC
GAGGATCTCAGCAGGAAGTACTTCCAGCAGTTGATATCCGCCGTTGGTTATTGCCATTCG
CGCGGCGTCTATCACCGTGATCTGAAGCCGGAGAATCTCCTGATCGACGAGAACGGGAAT
TTGAAAGTTTCAGATTTCGGACTCAGCGCTCTGACGGATCAGATCCGAACCGACGGGTTG
TTGCACACGCTGTGTGGGACCCCTGCTTACGTGGCACCAGAGATATTGTCGAAGAAAGGA
TACGACGGAGCCAAGGTGGATATCTGGTCATGCGGCGTCATTCTGTTTGTTTTAACGGCC
GGTTACCTGCCGTTTAACGACCCGAATCTCATGGCCATGTACAAGAAGATATACAAAGGC
GAATTCCGGTGTCCGAAATGGATGTCCAACGATCTTAAACGGCTGTTAAACCGTCTCCTT
CATATCAATCCTAATACAAGGATTACCGTCGATCAGATTCTCGGAGATCCATGGTTCAGA
AGGGGCGGGGTCAAGGAAATCAAATTCCACGACGACGAAAACGCCGCCGTTCCGGATAAA
ACCGGTAAGGAGGGGTTCGGTGCGAGGAATTTGAACGCGTTTGATATAATCTCATTTTCG
TCCGGTTTGGACCTGTCTGGTTTGTTCGATACGTCGTGCAACTCGTTCGAGAATAATACT
GGCGAACGTTTCATCTCGCGAGAGTCGCCTGATAATTTGTTGGAGACGGTGACGGAGTTC
GCCAAGGTTGAGAAATTAAGGTTGAAGACGAGGAAAGAATGGGGGGTGGAGTTGGAAGAA
CAAAACGGTAATTTCATCATCGGGGTGGACGTTTACCGGTTAACGGAGGAACTAGTGGTC
GTGGAGGCCAACAGAAGAGCGGGTGACGCCGCATCTTACACTGAGGTGTGGAAGAATAAG
CTGAGACCGCAACTTCTTGTGCGTCAACAGGAAGCTTCGGTTTCTGGTAATCATTAA
2>NISI03G1063
ATGGAGGAGAGAACGGTTGATGGCGGCGAGGTATTGGGAGGAGGGAACCTGTTCGAGAAG
TACGAATTGGGCAGATTAGTCGGTCGGGGAGCGTTCGCGAAGGTTTATCACGGCCGAGAT
ATTCGTACGGGACAAAGCGTGGCGATTAAGGCCGTTGGCAAAAACAAAGTCGTTAAAGAA
GGATTTATGGCGCACATTAAGAGGGAAATCTCTATTATGCGCCGGTTGCGCCATCCTCAC
GTCGTCAAACTGCTAGAGGTCATGGCTACCAAAACCAAGGTTTATTTTGTGATGGAGTTC
GCCAAAGGTGGAGAACTCTTCACCAAGGTTTCCAAGGGAAGGTTTAGCGAAGATCTCAGC
CGTAAATACTTCCAGCAGCTAATCTCAACCGTTGGTTATTGTCATTCTAGAGGAGTTTTT
CACAGGGATTTGAAACCGGAGAATTTATTGCTAGATGAAAATTGGGACCTGAAGATAACC
GATTTCGGACTCAGTGCGCTAAAGGATCAGACCCGATCCGATGGAATGCTTCACACTTTG
TGCGGCACTCCTGCTTACGTGGCACCCGAGATTTTGGCGAAAAAAGGTTACGACGGCGCT
AAGGTGGACATTTGGTCATGCGGCGTCGTTTTGTTCGTCCTCAACTCCGGTTATCTACCG
TTCAATGATCATAATATTATGGTCATGTACCGGAAGATCTATAAAGGTGAATTTCGGTGC
CCCAAGTGGACGTCTCCGGATCTTAAACACCTCTTATCACGGCTACTCGACCCGAACCCA
GATACAAGGATCACTATTGACGAGATCTTAAAGGATCCATGGTTCAGAAAAGGTTACAAG
GAGGTAAATTTCCATTCCGAAGATTCTGATTTGAAAGACTTGGGAGTTAACGACAACCGC

AAATGTTTAAATGCTTTTGATATAATCACTTTCTCTCCGGGTTTTAGTTTAACCGGCTTG
TTCAACGACTACACCGATGCCGAGAGATTCTTATCGGCTGAAAAACCAGATAAAATTATA
GAGAAAGTCGAGGAGATGGCGAAGAAGGAGAATTTGACAGTGATGACGAAGAAAGGTTAT
GGGATAAAGTTGGAAGGACATGACGGTAATTTCTCCTTGGTCACAGCTGTTCACCGGTTA
ACGGATAAACTGGTTATTGTGGATATTAAGAAGAGAGAGAGAGAAGTTGGATCTGGTCAA
GAATTCTGGAAAAATAACTTGAGACCTCGGCTTCGTGGTTTGATTTATCCACCGGAAACG
CCGGTTGCCGACAACTCATAG
3>NISI03G1060
ATGGAAACCATGGGGAGCATATTGATGGAACGTTATGAATTAGGAAGGCTACTAGGTCAA
GGGACTTTTGCCAAGGTTCATTATGCAAGGAATCTTAAGACTGGGATGAGCGTTGCCATT
AAGATAATTGACAAAGAGAGGGTGATCAGATATGGGTTGATGAATCAGACTAAGCGAGAA
ATTTCTGTTATGAGACAGATTAAACATCCAAATGTAGTCGAGCTGTATGAGGTCATGGCA
ACCAAAAACCAAGATTTACTTTGTGATAGAATATGTTAAGGGTGGCGAGCTTTTCAACAGG
TTGGCCAACGGGAAGCTCAACGAGGATGCTGCAAGGAAATATTTTCAACAGCTAATAAGT
GCAGTTGATTACTGCCACAGTAGAGGTGTGTATCACCGGGATATAAAGCCAGAAAACCTT
CTATTGGATGAGAATGAAAATCTAAAGGTTTCAGATTTTGGATTGAGTGCACTTGTGGAT
TCCAAGCGTAAAGATGGGTTGCTCCATACAACGTGTGGGACCCCTGCTTATGTTGCTCCA
GAGGTAATCAACAGAAAAGGCTATGATGGATCCAAGGCTGATATATGGTCATGTGGAGTG
GTCTTGTATGTTCTATTGGCTGGACATCTCCCATTCCATGATTCAAATCTGATGGAGATG
TATAGGAAGATTGGTAGGGCGGATTTCAAATACCCTAACTGGTTTGCCGCAGATGTGCGC
AGGTTAATCACAAAGATCTTGGATCCAAACCTGGTGACGAGGATATCTATGGACAAGATA
ATGGAAAGTTCTTGGTTCAAAAAGGGGTTAGAAAAACCCATAACTATTGATTTAGAAGGG
AAACCAGAAATCCCTGCGGATACTGACGCAACTTTCACTTTAAATGAAAGCAGCGGTGGT
CGTACTGTGCCCAAGGCGGAGAATGAGTTGACAAAGCCTTCCAACTTGAATGCTTTTGAT
ATAATCTCTTTCTCCACTGGCTTCGATTTGTCTGGTTTGTTTGAGGATAGGGAAGAAAGG
AAGGAAATGAGATTCACATCCAACAAGCCAGCTGCAACCATCATATCAAAGCTTCAGGAT
ATTGCCAAGCGTCTGAAACTCAAAGTAAAGAAGAAAGATGGAGGGTTGCTAAAAATGGAG
GGCCCAAAGGAAGGTTGGAAAGGAGTGCTGGGTATCGATGCAGAAATATTTGAGATCACT
CCATTTTTGCATATGGTGGAAATGAAAAAGAGCAGCGGAGACACACTCGAGTACCAGAAG
ATGATGAAACAAGAGATAAGGCCAGCTCTTAAGGACATTGTGTGGACATGGCAAGGGGAG
CAGCTACAGCAGCAGTGCTCGGAACATATAGCACTGCCATCACCAGTACAACAGGAAACA
ACTGCCTAG
4>NISI07G1958
ACAAAATCAGCAGTATAATTGCATTCCACACCGGCTCATTCGAATTCGTGTTCGCTTTTT
CAGGCTATCACCAATCGGACCCTGTCCTCTCCTTTTTTCTCTGTCCGTCGGAGGTAAAAC
GATCTTCGTCTTCGCCTTTCTGTTTTACTGTTTTTGATCTGAAATTGTTTCATGACTTGG
ATTCTTGTTGTTGTTATCTGCATTTCGTTTTACTCTCTGGATCTATTTTCATTTGTTTTT
TCTGATAGATTTTTTTTACTCATACATTAAACTTCATTATATTATCGGTTGGATATTTTT
CTTTTGCGATTCAGAGGAAATATTATTTTGCTTGTACGATTTTCTCCGTCGCTTGATTAA
AAAACTTGTTACTAGGTCTGTTGTCTAGCTTGCGTTTGATTTTGTTCGTCCATTATTTGA
ATTCATGGCTTGGTTATCTGCATTCAGCTTCGTATTGTTCAGTGTTTATATGTAGATGTG
ATAGCCGTTTTCCTTGAGACGCTTTGGCAAGCCTCCGTTGTTTCATTGCTTGAGATATCT
ATAATCTAGTAGGTTGAATATTGATGTTAGCGGCCATGAATTTGAACTTGACATTCATTT
AATAAATTAAAGGTGGTCGGGTGTGTGTGAAAATAAGGAGAAAAAAACAAAAAGCAACAT

GGCTATGAAGAAAGTGTAGTTCAAATGTGATCACCGATTTTTCAGATCATTGATACGATT
GGTTCTCTGTTTTCGAGGAAGACTGTAAAACAATTCTGCATATGATGTTAGAATCCACAT
GTGTTATTTTGCCACCTAGAGAAACCAGATATAATTTGTAAAGCAAGCTTCTGATGCCAA
TCTTTGCGAGGTTGTTTAGTACCGTTGTCGTATTGTACCAGTGATCTGATTAGCAGGGTT
TCTATGAGTGTCTAGATATAATTCTAATATAAATTTCCTCTTTTCAAAACATGTTGCTTA
GTGAAGCTGTTTGTTAAAATTTCTTTGGCTTCTGATAGCTATCAATGTCTATATGCCTCA
TGGTTCTTCATCCTTTTGCTTATAGGTCTGAAGGCCAATTCCAGGGAATGCAGAAAGATG
AGCTTAATTGCTGAAAATACTTCAATGATGCCTGTGGGCTGCCATTATTCATCTTCAATA
GGTTCTTATACTCAACGAAGAGTGGGATTCAGCTCTTGCCCCAAGTGCACGATGAAACAC
CAAATAGAAATCCAGTTGTGTTGAATCACAACTTTTTTGAGGACGGTTAGTTCTTCAGCT
TTCAAAAGCTCCTGCATAACGATTCATATCGTACTTGCAAGAGAGAATAAAATTTATATT
TGCTAGGGTGCATATTAAAAAATAATAATAAAAAAAAATAAATAAAAAAGAAAAAAAA
AGAATAATGGAATACAAAGGGAAGGTGCTGATGGAGAAGTATGAGTTGGGGAGATTGTTG
GGCCAGGGGACCTTTGCTAAGGTTTACTTTGCTAGGAATCTTGAAACCAGCCAAAGTGTA
GCCGTTAAGGTTATTGACAAGGAGAAGATCCTTAAAGCAGAATTGACTGAGCAAACCCTA
ACAGAAGTATCTGTTATGAGACTAATCAAACATCCAAATGTGTTGCAACTTTATGAGGTC
ATGGCCACCAAGACCAAGATTTACTTTGTCATGGAATATGCCAAAGGTGGTGAGCTCTTT
AACAAGCTAGCAAAAGGAAGGCTTAGAGAAGACAGAGCAAGGAAGTATTTCCAGCAGTTG
ATAAGTGCTATTGATTTTTGCCACAGCAGAGGTGTATACCACCGTGATCTAAAACCAGAG
AACTTACTATTGGATGAGAATGGAACACTAAAGGTTTCTGATTTTGGGTTGAGTGCTCTC
TCTGATTCGAAGAAACAAGATGGGTTGCTTCACACTACTTGTGGAACCCCTGCCTATGTT
GCTCCTGAAATCATTCGTAGAAAAGGCTATGATGGAGCAAAATCAGATATCTGGTCTTGT
GGGGTTATCTTGTATGTTCTTTTAGCCGGTTATCTCCCATTCCATGATTCAAATTTGATG
GCCATGTATAGAAAAATCAGCAAGGGAGACTATGTGATCCCTAGTTGGTTTTCAACAGAT
GTGCGAAGATTGCTGACGAGAATCCTCGACCCTAACCCAAAAACCAGGGTTTCCCTTGCT
AAAGTTATGGAAAACCCATGGTTTAAGAAAGGATTGAATTCAAAACCCCTTGAAGCTCAA
ACAGAAGTTATTGAGAACGTACCTTTGGATGGTGATACAACTTTGGTTCCTCCTGAAAAT
GGTGCTATTAACGATCCAAAGAGAGAGTTGATTAAACCTGCTAAGATGAATGCATTTGAT
ATCATCTCTCTTTCAACTGGGTTTGATTTATCTGGTTTGTTTGTGGACAACAACCAGAAG
GAGGAAGCAAAATTCACGACGACACTCTCAGCCTTAGATATTATATCTAAACTAGAAGGT
ATTGCTAGAGATCTGAGGCTGCAAGTAACAAAGAAGGACAGAGGGATGTTAAAATTGGAG
AGACCAAAGGAAGGTAGAAGGGGGGGGTTGTCCATTGATGCAGACATATATGAGTTGAGT
CCTTCCTTTCATTTGGTTGAAATGAAGAAGTCTGCTGGTGACACTCTGGAATATCATATC
ATGTTGAAACAAGACATTAGACCAGCTCTCAAGGATATTGTTTGGGCTTGGCAAGGGGAG
AAGCCCCTGCTACAACAATACCATCGGCACTTTTTTTAGGTTTGTCCGTTCGTAAGTATT
TTGTTCTTTAAACTGTCTTGGAATCATAATTTTATTCATATATTTGAGTTTTCTCCAAAA
CAACTGCGGTTGGATTTATGTGGTATCCTTTTGGCCATCAGCTCAAAGGTTACTATTTCT
GCCGCAAACTATTCAACATTGGGCCACTAATATGTTAAACAACGAAATATCATGTTTCTT
ATTGTTTTTGTATCAATCGTTTCCTTGTACTGCCTTTACTCAAGTGTGTAGCTGTTATAT
ACATAATCTGGAATTGTCTATTTTCTTTTGTCTGATTAACGATTGTCCTCTTGATGTTTT
ACTGTCATTTTTGTCTAGTTTACTTTAAGGGAAGAATCGTATTAGGAAAATTTATTTTCA
TTAGACATAATTTAATGAAATAAAACATGCCGTTAATGTATAGCTAAAGAGAACATAATT
ACTACCGGAGGTTGTTAGCTCTGGTTGTACACACTGAATTAACAAAATGTTGATCTTCTA
CAGATGTAGTTCGGTACAACTGAATCTAAAATAGGTAACCCCAAAATTATTAGTTTAAAC

ACTTAGAAAGTTTATTGAGGATAAATGGGAATGCTCAATCCACTCGATTTTTAGTATCCA
GAAACAATCTCTTCATAAGGGCCAAAGGCTATCATGATAGGGTGAAGCAAATTGGAACTG
GACCATCTAAATCAGGTCCATTCTATGGGTATATACCCATTCTTGTCTTTCGTACAAGGT
GATTTGAAGACCTTTGATAATTTTGGAGTTAGATAGTTTCTCATTCTTAGATACTTACTG
GATAGTTCCTTTACATACATAAAATAATTGCTGAAGTTTTTGAGGTAAGTAGATGGATGG
ATGCTAGTTTCAACTCAAGTTTGTTAAGTGGATTCAGAGGATCTGGAATATCAGGAAAAC
ATATAAGAAACATTCAGTCTCCGAAGAGTTAGAACTTGAGCGTGTCAGCTGAATTACGCA
GTTGGAATTGGCACGGCAGAGGAAAGGTTTTCCTCTACCAAATATCTTAAGTAGTCTAAG
ATAAGCTTATCTGAAATAAAGATGACCACGCGGGCTTTGAAAATTAAGATGCTAGAAGTT
ATTAGGAGGTACTGAAAGGTTCATGCCAACCTCACTTGAGGGAACTTTATATTGAGTCAC
CTATTTCACATCTTGGTAAGAAAGAATCCTATCTAGTTTGAATACCCTATAAATCCTTAA
ATTGCCATGGGTCTTTTGGTTTACAAAAATCCAAATACAAAATCATGAGGACTGTGTGTT
CAAAACAAACACATCTAGTAATAGCTTAATAGGTCGGAGTGTTCCTATTGGTATCAAAGC
CGGGTTGTGACACGGCATTGAATTACTGTTCCATCCATAGGTCTGCCTCTCGTAAGAACA
TGAGATCCAAAAGAGGTGATTGAGGGAGTCCTGTTGTGGTGAGCCACCAATCCCACATCA
TTAGTAAGAACAAATACTAACTAGTTATAAATACCCTATGAATCATTCAAAATTCTCAAA
ATCTTTTTGGATTGTAAAGCCCAAAAGCAAAATCATGAAGACTGAATAATATCTAACAAT
GTGTCGAGGTGTTTCACCGTTCTTGTGTATACATGCTTGTGCATGTATCTATATATGCTA
TATTCAGTCTTGATGAATATGTAATCCTACTCCTCAGATTACTCGAAAACCCCTTTTAAA
ATTTTTTCACTATAGACTGATGTTAGTGGAGAGAAAAGTTTGAGTGGCTATTTTCGGGAA
GATCCTAGAATTGGATGATTTATGCAGATATTAACTCTAAAAGAATTTCCAATTTTATAT
TTTTGTAATGGAAAATTATTGAGTGAATCTCCTGTTTGGAAAGTTAGTGAAGTAAAATCC
AAGACTTTATAATTATATGACCAAACTAAATCCACATTAGTAGATGATTAATAATAAACT
ATTGAGCTCAGAAGCCTCAGATACATCTAATCTGATCTAAATTTCAATGTGGCTGGCTGG
ATTGCAATTAAGATATTTTTTATTTTTATTTTTATTTTTTAAAGGAAAAAGAAAGATTTC
AACTTTATTTTGTCCCTACACGTTGGCCTTGGTCCTGGCTTTATGTCATATTAATGCCTC
TTGTTTCAGTAATGCTGTCACTTTTCATTCTTAATATTATTCTACTGCCTGCTGTCAGTC
TTTATTTATTACATTATCTCTTTTTCTGGGTATGATTCCATAGGGGGACCTAAAATCTTT
TGCTGAGTGAAAATGTTTCATTCCAAATGATGAGCCCACATGTCAAGATTATAGATATTA
TTATAAAATTAAGTACCCATTACCATAATAAACCCAAAACCCTACACAAATAAAAAAATT
GAAAAATTATAAATAAATAATTTTTTTTTTTAAAAAAAAAGAAAAAAGAAAGAAAAAGAA
AAAGAAATATCATCGAAAAATTTAAATGGTCCCCAACCCAGAAGAAGAGGGTTCCCACTT
TGTTGTTTGAAGCATCCATTCCTAGGCTTTTTTCCTGATATGACATGCTCTAATCTCATT
GTGCAACGTATCCAATTTCATATGAGTAATGTTAAGGATATTTAGGGTATTTTTCAGATT
TTATATCCTATCTGATATATTAGTGAATCTCAATAATAAATAAAAGGATAATTACAGATT
GGTAGTTCAAAATTCATATAATTATATCTGAGTACTTGAAATTTTAAAAACTAACACTTA
GATACCTCAAATTTTGAAATATTGCATTTTATAGCAAAAATGTAAGCAGTTTTGGTACAA
ATGAAAGCAAAATATGATCAATTTGATACTCTCAAAATTGTTATTTTTTCTATATTTTGG
GTACTAAACTCGTAATTTTCACTTTAAAATTTAAGATACTCAAATGTTAATTTTCAAATT
TTAAGATACTCAAGTGTAATTATATAAAATTTGAAAAAATAAAATGTATTTTCTCTAAAT
AAAATCAAAATCATGTGCTTTGTGTAATAAACATCAAAGATTTCGGCAAAATGAACTGAC
ATGTAAAAATTACGATAAAGAATAAAAATGTCTACATCGTTTCCTTATGTTATAATTACT
CTATACTCTCTCCAAACATCACATGAGTAGAAGACAAATTCACTAAAAGAGTTTGCCCAC
TTGGGCCTTGTCTCTAGAAGAACAAAGTGAAACTTCACTGATTGGTTGTGGGACCTGATG

CAGCTTTTTTTTTTTTTTTTTTTTTTTTTTTTTTTTTTTTTTTTTTAAAAAGAATAATTA
CAACTATTAAGATCAATTATTTAGATTTTTTTTTTCTTTAGCGTGACTTAAATATTTAGT
CTCAAATTTTGGGACAAATTTATTCTCACAAGGAAAGATATCTACCGACATCTCTGTTTT
TCATAATTGTAGAAAGTGAGAATTGAATCTGACCACGTAAGTGGTAGAGAAAGTGAAAAA
CTATAGTGATCAAATACTCTTTTTTTTTTTTTTTTTTTTTTTATATATATATATCTTAAT
TTAATTTGATTGGTTATTGTTGTTTTCATGGCAATTAGGCCATTCTAATATCTCCATTTG
ATTGATGTCTAGATGCTACTTGCAGTTGGCGAACTTTACAGCAATACAAATTCCTGTGTC
AGAACTCATCCACATTATGTTCTTCATTATTTGTGTCTTTATTGGTATCTCTCATAACCC
TTTTCTCTTTTATTACGATGTGGATACCTCCAAGTGTCGTTATATGTTATTTGTCATTTT
ACATTTAGTCTAGATTCTCGACAACATGTTACATAGGCACGACTTAAAATTGAAGGCTT
5>NISI11G0565
TGAGTGATTTGAATTTCAATTAGCTATATTAAAATGGGACACATAAATTCTTTATTTATT
TATTTATAAAATAAGATTTAAAAATATTACCTAATGATGAGTGATTTGAATTTCAATTAA
CTATTCTAAAATGCGACACATAAATATATTTTTTTCTGTCTTTAGCCCAACTGGGATATT
CTTATATTTTTTATTGTGTCTAATTCTCAATTAGAGAGAGATATATTATAGCTACTTCAT
CTTTCAAATTTCAATATAATCTTTCAAAAGTCAAACTTTAGCTAAGGTTATTCTAGAGTT
TAATATATTGAGCATTTATTATTATTATTATTATTTTTTTTTTTGTGTGTTTCCATATTT
AGTCTGATAATATCATTTCAATTTATATTATTATAAGGAAAAGCAAACAGGGAATTTAGT
TTTTTTTTTTTTTTTTTTTCTAATTCCTTTGTAATAAATAGGGGCTATTTCGTAAATTGA
AATTTTATTTCGAATTAAGTTTATTGAATCAAAGTAGTTACGGAACATACACGGAGCATT
ATGATTGGCCAGATCAAGCAGCAAACCCAGGCCCACATCATCCACGTGGGTCCCACATTT
CATATAATACAAGGAAAATCCAAATCTTCTTCTTCTTCCACCTCGAAACATCTCCACTCC
GTCCCTCCTTCCCTCTGTATTTAACACCCAAACTCTGCAAAGAAAAAACAACGTTTGCAG
AGATTAGATAAAACCAACCTTGTGCGCGTCGCTTTCGACAGTTATCCGAATTGCTTCCGA
TTAGTTATAAGTATCGTCAGCCGGTGTCGTTTGGCAACGGGAGATTTTCGTCGTACTTAC
TGATCGGACGGCGGTGTCGGAACGATTGAAAGCATGAATAAGGTACCGGGGACGAGGACA
CGTGTGGGGAAATATGAAATAGGAAGGACAATTGGTGAGGGTAGCTTTGCCAAGGTGAAA
TTCGCCAAGATTGAGACCGGAGAGTTTTTCGCCATTAAAGTGCTCGACCGTGATCAAGTC
CTCCGTCACAAGATGGTCGAACAGGTATTTTTCAATCCAACCGTACGCACATATACTTTT
CCATGTTGCGTTTCTAGAAGTAGGTTAAGGGTAGTAGCTTCTTAATAAAACCAAAAAAAA
GGTTATTCAAAATGAATTGATTTTGTTAATCATGACTAAGGATGGATTTCTGTGACCTTC
ATTAGTGTAAGTTTGCTTGATTGTAAGACTTTTTTTTTTAAGATGAGTGGACATTGAATA
TGCAAAGCTGTCGGTCCTATCAACCCAGGTGGGCCCATTTTGGGATAAAAAGGCAATTGA
AAATTTTAAAATTCAATCTTTTTGTGGGTATCTGTTGATTTATTAATCATTTGAGACTAT
ATTTTTATTCCGATAACTTTCTTAAAACCAATCGATTATCAGGGGACGCCCAATAATGGA
TAAATTCACACTGATGTGTATACTGTATCTGTATGAACTAAGGTATTGAACCATGATGGA
AGAATTGACTTTATTTTTTCATCATGTTTTTGATGTTCTTATTTGCAGATAAAGAGAGAG
ATATCAACAATGAAGCTGATCAAACATCCTAATGTCGTGAAAATGATTGAGGTACCAAAA
GTTTTTCCCAAATTTATTTTTTATTTTGTACCCGGTGCTATTTGTACTGTTCTAGTGATA
TAATCTACAGTATTTGAGATTGCAGAGTCATTAGTGTAATTGACAATGCATTTTTAGGTT
GGAAAGTGACTAAGTATGATGCTTTTATTGTCTAATTACCAATAATTATTGTCTTCTGTC
TTGAACTATAGGTTATGGCAAGCAAAACAAAGATCTACATTGTTCTCGAGTTTGTCGATG
GGGGTGAGCTCTTTGATAAAATTGTAAGACACTTTTTCAAGCAAATTGCTTCTTCTTATG
CTGAGTTCGTGTTAGTAAATAACTTCTTGATCGGTCAGGTATTAAAATATGTACATTCTT

TGATTGGTATTTTGATCCCTAGGCAAGGAGTGGGAAACTCAAAGAAGATGAAGCAAGGAG
ATATTTCCACCAGCTCATTAATGCTGTGGACTATTGTCACAGTAGAGGGGTGTTCCACAG
AGATTTGAAGGTATAACAAGTTTCATCTATTGAAATCATACTTTTAGGTCATTTATCAGG
GTTGCTTGAATCATTAATACACTGTACGTTTGCAGCCGGAGAATCTTCTTCTTGACAGAT
CTGGCGCTCTGAAAATTTCAGATTTCGGTTTAAGTGCGCTGTCGCAGCAAGTGCGGGTAA
GGATCATTGAATGACTTTAAGAATTGCGAAGAATCATGTAAAGATTGAGAAATAGAGTCA
TAATTAACATCAATTGCTCATACTGCAATGTAGTCGATATTATCCGACTCTTCTTTTTTC
TGATTTCAATTTTGCTCATACTGCAATCTAGTCAAGGTCTCAGTTATAAAGACCGGTTAT
AAGTGTTGGGTAACTCATGTTATTCTGTGACTAGTTCATTATCTTAGCTTTTTGTTGGGG
GGATTATCGAACTCTTTATTCAGTTTGCCAATGTACCTTGTGGTTGTTACTATTATGTAA
ATGTTTACCTTCGGACCATCCCCCGGAGTTAGCCTATATTTTTCAGTCACTTGCATAATG
TAGCTCAATATATGAAAATTTGAGAAAACAACCCTTGCATTTGTGCACTGAGCATGCAGA
GCTAACCAATATTCTTTTCATGGCAGGAAGATGGGCTGCTTCACACAGCTTGTGGGACTC
CAAATTATGTTGCTCCTGAGGTTTTTGCCAATGACTTGTTGCAGTAATGCAATTACAGAA
TTGTTTCTTAATATGCATTTGTTTTCTGCGTCAGGTGCTTAATGACAAAGGCTATGATGG
TACTGCATCGGATGTTTGGTCCTGTGGAGTCATTCTCTTTGTCCTGATGGCAGGATACTT
ACCTTTTGACGAGCCAAGTCTAATGTCCTTATATAGAAAAGTAAGTATATTTTGCTTTTC
ATAGTTTGCTTACATGATTAAGAGCAAAAAATTAACTCGCCCATACTTGTTCCAGATATG
CAAGGCTGAGTTCTCTTGTCCATCATGGTTCTCACCTGGTGCTAAGAAATTGATCAAGCG
TATTCTTGACCCAAATCCTCATACTGTAAGATCCAAAATCCCTGCCTAGTTTGGATGTAT
GATTGGATTATTGACATAGGAATATCATGTATTTGATGGGATGCATTAATATTAGATGCA
TGTCTATTATTGCTGATGCTATCTTTTGTTGCAGCGAATTACTATTTCGGAAATATTAGA
GGATGAATGGTTTAAGAAGGGGTACAAGCCACCACAATTTGATAAGGAGGAAGATGTTAA
TCTAGATGATGTGGATGCCGTTTTCAATGACTCGAAGGTGAGAAATTTATATCATGTTTA
CTTTCCATATCTTATGTGCTTAAACCTACATTATAAGGTAAATTTCACGAAAGGTATCTG
TGTTGTTGAAGTTGAAGATTTTATTTTCGGAATGACCAGGTTAGTCTGTTTTGTGAACTA
AAATTTAGCTGTTTTGAAGAAGGAAACCTATGGAAATTTCATTTGAGATTCTTTATTTGT
CTTTGTGCTTTGGCAGGAATATCTTGTAACAGAAAGGAAGGAGAAACCTGTATCAATGAA
TGCTTTTGAGCTAATCTCGAGGTCACAGAGTTTTAACCTCGAGAACTTATTTGAGAAGCA
GACGGTATGTGTTGTGGGATTATAAGTTTTAAGTTCAAATTTATTTTACTACGGTGATGT
TGTTGGAGAGATTTCCTTAACCAACGTTATCATTTCGGATCGTTTTCCATCTCTGAGTTA
TCGAAAATGAGAGATGTAGACTCTATTAGTCCTGCCTGAATAAACGCTCTTACTTTTTTG
AGAAATGATGTCCATAGGGTCTTGTGAAGCGAGAAACGCGTTTTACTTCCCAACGCCCGG
CAAATGAGATCATGTCTAAAATTGAGGAAACTGCAAAGCCTTTGGGCTTCAATGTTCGCA
AAGGAAACTATAAGGTAACATTATCTCTTATTCTCTTGCATCCTTCCAATTTGCTTTTCA
ACAACTTGTTTCTATTCGAAATCACTCTTTCTGAACTTTCAACATTTATTTTGTAATCAT
TCGCGAATCTGTTTCATGGTTTCTCTGTATCTATTGCAGATGAAGTTGCAAGGTGACAAA
AGTGGAAGGAAAGGCCAGCTCTCTGTAGCTACTGAGGTACATAACTTTTGCAGTCAACTT
TAACTTTTTGAGTTCCACCACAATGAATGAAATGCATTATTTATGGGGAAGAAGAGTGAG
ATTCTACATCTGGTACAGTTACTCTTTGAAGCGTGTAATTGGAACATACTATCTGTATCT
TAAAACGGAGTATTTCTATGATTTTTATTGAGTTGACATAAGGCAGGCGGGACACACATA
CTATAGGAATAATGATATTTCAAAGGCAGGACATGAAAAATACATCCCTTCCTTTTCAGC
GATAATTGCAGTCGCAGGCTTGAGTGATGTACCTGTTGTATACAGAATGCTCAAAGATCC
TACTATCTCATTATTCCAAGCTGCATGCACCAATGATGTTTGCATATTAAGATTTGATGA

ACAGATTAATAATATCAGTGATAGCTTACTGGATTTCATAAGCTTTCCTTATTAGTGCAA
ATTCTAGCTGTAGATTCACTTCCAAACGATGTGAACTTGACGATTTTGGGTTGGGTTTGT
TCAATTCTTGGTTTTATCATTTCTGTATAGAATTACAAATTTGGTAAACCTCATTCAGTT
GCACTAACTTGGATCGAATTAAAATCTAGTTCATTGATGACTTCGATATATACTTTACGG
CTTCCTTCTATAGATTTTCCACCTAGATTTTTCATATTTAATATCAATTAATAATTATTT
CTGATTTCAGGTGTTTGAGGTGGCTCCCTCTGTGCACATGGTGGAGGTCCGTAAAACTGG
TGGCGACACACTAGAATTTCACAAGGTACCATATTTTACACTTTATACGAATCTGTTACA
TCTCGCAATGAGAGATAATATCTGCATTTTGAATCTTGAAATGTCTTAATTAGCAACATA
TGTAGGTCGTAGTAATCTTTTTGTTTTCTTTTATTATTACTATGAATTCTTGAATCTGGA
ACGGTTTTGACTTGCCAAAGTTATTGAGGACTAATACCGTTAACCTATTCCCTGTTCATG
CTGCAGTTCTACAAAACTTTCTCATCAGGACTGAAAGATGTAGTCTGGCAAACAGAAGAA
AATGACGAAAAAGTAATGTGATTTCGCTATCTGTCCATGCACATGCTCGTTTTTTAAAGC
TTAAAAGCATAAAATATCGACTCTTAATTCATTTATTCCCCTATTTTGCAGGCTGTAAAG
AAATCACGTTAGGAGGATCGTGCTGCTTAGTCGGTCCTTCTGATTTTGTTACAGGCCATT
GTCCATACAAACATACCATACCCATAGAAAGTTTGAAGGGTAAGGATGCATGACTAGCAC
AGGCCATAGTTGTGAAAAACGAACTGAAGAGCCCCAAAAAAAAAAAAAAAAAAAAAACCAT
GGTTTTTGTTGTGTATCTACTGTGCCAGTATGTCTTTTCTCTCCTTAGTTTTGAACTCCC
ATTTTATGTCCTTCAGCTTAAGCATTTGGTAAAAGTTTCAGGGGTCATAACTAAAGTCAT
ATAAACATTGGTGGGATATTTGAGAACCGCCTCTTTAATCTATAATGCATGCAAGAAGAG
AACTGGTTTTTTTGCATTCAATGTGTTTATGATGAAGAGGTTGTCATTTCTCAACCATTT
GAAAGAGAAAGAAAACCATCATCATGATTCATAATAAGTGGTTTCCTTTCAATTGTAGTA
AGAATGTTCATCTTACCATTATATGTCCTTCTTTCTGTCTTATTTGTTTGATAATGATCG
AACTGGTCTTATGAAATAATTTGGTTTATGAGGAGGTTGTCAATCGTCAACCATTCAAGA
GCATAAATTATTCTTACGTTCTTTTTTAGTCGTGATGCTATAATGTATGATATTGTAGTT
ATGTGTTTATGATATGTTCGATCATTGGAAAGATATGAGGTTGAAATGCT
6>NISI06G1821
AAATCTTTCACTCTCCTGCCGCTGCTGGTTGTGTCTTTTTCTCCTTCCCAAAACTCATTT
TCTTTTCATTTAATTATTCAAAATTTCAAAGACAAGATTGGAAATTTAGTTGCCCCACTT
CCTCGCCTCGTCCCTTTTTCTGCTTCAACTTCTCTCAATTTCGGACCAACAAATTCGTCT
TAAGAAACTCCATTTTCCAACCAACCAATAAAGTCCCTCCCTTTCCCTTGCTTTAAATTT
TTAAATCCAATACTTTCAATTTACAAATTCTCTAATTCTTACTCTTTCGCATAATCATAT
TAGTCTCTTCATCAACCCACCAACAGCGACATCTAAATCCAAATACTATTTTTTTATTCG
AAAGGGAAAAAATGAAAAGCCAAAAAGCTTCCCACTAACATCTCTTGGTGCTCTGTCAAA
AATGGCGAGATTCAGTTTCGTTGAAGAAAGATAAGCAGCTGCTGAGAAGTAATTGGAGAA
AGAGAGAGAGTTAAAGCAGAGAAGGGAAAATGGTGGTGAGAAAAGTCGGAAAGTACGAGG
TCGGAAGGACGATCGGGGAAGGAACATTCGCCAAAGTGAAGTTCGCTCAAAACACGGAGA
CAGGGGAAAGTGTTGCCATGAAAGTTCTCGATCGAAGTACCATAATCAAACACAAGATGG
CTGATCAGGTATTGTCTTGTCTGAATTTCTATATATTTTTTTCCACTAATGGATAAAAGT
TTATTTCTGGGGTTTGGAATTTAGTTTATTTTCCATTTTAGTGGGTTTTCTTTGAAATTA
ATTAGTACTAGTAGTAGCTTGTTATCCAGTGTTGAAGTTTTCAGCTTTGGTTTCATTAAT
GAATTATGATTTAGTTAGTAGCACGGATTCAACTCACGGATTAACCCTGAGCTGACTGAG
TACTTTAAATTTTACTAATAATATAATTATCAATAAGTAAAATATTTGGGGCTAGAGAAT
TGCCAAATTAGGGTTTTGTGAACGTATTATGCTATATTTAGACAATTTGACGAAGAACTA
TTTACTTTGACCAGGCATAACCCTGCAATTACCGTAATTGGTAGTCAAAGTTACTGATGT

TCTTGGGTTGGCAATATTTTCAGTATCAAGAATCTAACTTGAACTTTCATTTATGGATGA
TAAAGATATAATCTTTTTTCCTTTTTAATTTGGTTTTGCAGATCAAGAGGGAGATATCGA
TAATGAAGCGTGTCAGACATCCTTATGTTGTTCGTTTGAACGAGGCATGGGATTTATGTT
CATAGTTTAGAATACTGACTTAACTGTAATGTACTCCAAATCTGACTTCTCATTTTGATT
TGTTAGGTTCTCGCTAGTCGTACTAAGATTTACATTATCTTGGAGTTCATCACAGGCGGT
GAATTGTTTGATAAGATAGTGAGTGGACTGATTTTGCATCATCCGATCTTCCAATATTTT
AAAAATAAATGAATAAGTTGACGTTGATCTTGATCATGTAGGTTCATCACGGACGTCTTA
GCGAGGCTGAAGCTAGGACGTATTTCCAACAGCTTATTGATGGTGTAGATTTTTGTCACA
GTAAGGGTGTCTACCACAGAGATTTGAAGGTCTTCTTTGCTGGCATTTTTGAAACTATTG
TTTTGTTCTGCTTATGCTTAATTCCTTACTGTTTGGTTACTTTTAACCTTGCAGCCTGAA
AATCTTTTACTTGATTCTCAAGGAAATCTAAAGATATCAGATTTTGGTCTAAGTGCATTC
CCTGAGCAAGTATGTTATTGTCTGAGTCTCAATTTCACTTTTCTTGATAACTCCGAGAGG
AAACCTAAACGGGTCGTTAATCTATTGTCTACTTACTTGTGTAGGAAAATAGCCTACTTC
GCACCACATGTGGGACGCCTAACTATGTAGCACCTGAGGTAATTTCGAAGAATTTTTTCT
GGGTTAATTGCAACTTTACTTTTTGACAATTTATGTTCACCGTCTTTCTTTATTTTCTTT
TTATTTGTATCTTTTTGGCTGTTTAGGTGCTAAGTCACAAGGGTTATAATGGTGCTGTGG
CTGATGTGTGGTCTTGTGGGGTCATCCTTTATGTTTTAATGGTTGGATATCTTCCGTTTG
ATGAGCTTGATCTCACCACTCTGTACAGTAAGGCATGATAATTTCATCGTTCAATTATTT
TGTCAGAAGCTAGTTTTGTGATAGACAGTAGATTAGATACTGGGAGTTTATATCATTATG
AAACTCGGTTGATTATAGAAAAGCGTATACTCTATTTACGGACTTTACTCTAGATTTTAT
TTACAGCTTTTGTCTAATGCAAGCAGTGATCTAGGATGTGGCATCATTCATAAATAAGAG
TTTAATTTAAATTAAATTAAATTAGTGGTTTTCTGCAGGTTGAGAAAGCAGAATTTTCAT
GCCCATCTTGGTTCCCAGTGGGGGCAAAATCTTTGATCCATAGAATATTGGACCCAAATC
CTGAAACTGTAAGTATCAGCCATCTGAAGACTGCTCTCTGTATGAATTTATATACTTTGT
TTTTCAATGACACAAACACATATGGTAGTCTGATACAATATTTGCTTGAATAAGATTTCT
TTAACCAACTGAAAATCATAATGTTTACTAATGTACGGTTGTCAAAACCAGTCAAATTCT
TCTAGAAATAACTATTTGTTGTCCCAGTTACTGGATTTTATCAATTTAGGCTCATTATAA
TTAACACAGTTTGATTAAGAATCATTACCCTCAAACAGTGTTGAAGATGGTCATTGTGGA
TAATGAATTTTGTCTCTTTGCAGCGTATTACCATTGAACAGATAAGGAATGATGAGTGGT
TTCAGAAGGGTTATGTTCCTATGAGACTTCCAGAACACGAGGATGTGAACTTGGACGATA
TAAATGCTGTTTTTGATGATCCCGAGGTTGGTAAGGTTTTCATACAGTAAAATTTCATTC
AGAAGCTTTCAAGGTTTAGTCGTAGCTATGAATATCCAATCATTTATCGGTATCCAATTG
TCTTCCCCTACTATGGGAATCATTCTTTAGCTTAATCCTCAAATTGTCAAATTCCCCACT
CTCGTACTTGCTTAATGGTGAGGAAGATTGAAATTTGTCTCACTGGAATGCTCTTCATCA
ATCCTACGATCAATGATTGCTTGCTCACACGAAGAGTACCCAAGTTTTTACCGATTTTGA
TCTAAGTGGTGTGGTGCGGGATTCCAACAAAGGTTATAAGAACATCATTAACGTAGCAAT
TGGTGGAGTTTGATGAGTTGGGATGCAAAAGTTGAGGCATTTATTTTGGTTCTTTATGAT
ATAGTTAATTAATTTTTTGTTCACCATGAGGTTTGACTCTTCAGTTAATCAGAGGCTTCT
CTGTTAAATATATGAAAGTGGTCTTGGTGATATATGATAGAGCAAATCCCTTAAAAAGAA
TATACTTTTTGAGCAATATGGATGGCTTTGGATCAACTCTTTTTGCTCTGGCAAATGTTT
ACAGTGATTTCCCTTTTTAATATCTTAACAACACTGGTAATATGGGCATTTCCAGGAAGG
GCAGGCAAACGAGCAATGTGGAAGTGGGACAGAGGACATGGGTCCTTTAATTCTCAATGC
GTTTGACTTGATTATTTTATCTCAAGGCTTAAACCTCGCATCACTTTTTGATCGTGGGAA
GGTACGGATATGTCAATAAGGCAATCTATTGTTCCTCGAATCTTATGCTAACTGCTTGGC

TGTCGTAACTTTCTTCATGCCATGGAACCTGCTTCACTGGCTGTCAGATAGTCTCCATGG
TAGTGATATTGAAAATAAATGAATGCAACTTCTTAATAGTGTACAGCTAAGCATTTGTTA
TTCGTTTCAATAGGAAACTGTGAAGCATCAGACCCGCTTCATTTCACAGAAGCCGGCGAA
GGTTGTTTTATCAAGTATGGAGGTTGTTGCACAATCCATGGGTTTTAAGACACATATTCG
CAATTATAAGGTAGAGAGGCAGGCAACTCCTCGGGATTTACCATGTTATGCATTGCTGTC
ATTGCTTCTTTTTAACAATTTTTTTTTTTTTTTTTATCCATGGTTCTTCTTTCCTCTGG
TATTCTATATATTGTTTGATATAAAATATGTAAATGTAATGGTACTTAATGTTGAAGAAC
AACTTATCTGGGTTTGAACTCATTTGATATCTTCTTTTTGATGTTTTAGATGAGAGTTGA
AGGCCTTTCTGCAGATAAAGCTGGTCATTTCTCTGTCATCCTGGAAGTAGGTTCTGAAAC
CCATTTTTCTGTCTAAAATTTATCAGCGTTACTCATAACTTGATCTACCAGATTTTATGC
TAACTTTATGAATCGGTTTGAATCGAAGATTTTTGAAGTGGCACCGACGTTTTTTATGGT
GGACATTCAGAAAGCAGCTGGAGATGCAAGTGAATACCACAAGGTCAGTAAGCGTAGACT
AACATTCTCGCATTCCTATGGCATTGAGCTAGCAATTCATATTCACGGTGGTCAGTTTTT
GAGACACCATGGAACAACATTTTATTTTGATCTCGGACTTGCCCGTGTTAGTTTTACAAA
AGAAAAACGAAAATTTTATTTATTTATTTTGTTCTTGTTAAGTTTTGGGGTTTTTTTTCT
TCCCTTTTTCTCTTCAAGGGTTTTGCTTTGTTTTTGCAGTTTTACAAAAACTTTTGTAGC
AATCTTGAGGATATCATCTGGAAACCCCCAAATGAACCATGCAAATCAAGGATCACCAAG
TCAAAGAGTAGAAAGCGTTGATTTCTTTTATGACAAAAACCATATTCTAAATCTGATACA
GGGGCTGACAATCAGGTCATTAACAGAAAGAAATTAGTAGAGAAGTGTTTGTGTTAGTAT
TTTCTACTATAGTGTTTGTATTGAGATGACAGTTTTTGATGAGATGATAGTTGCTATAAA
TATACAGAAAATTAGCAAAATACATGCTGATAGAGTTGTGTTGTATCTGATGATATTGCT
CCTGTTAAATTGCATTTTATTGTATAGCATTTTTGTATTGATACGTTTACTAAAGAACAC
TGGAGTTTGCTTTGATGGACTATTGTTCC
7>NISI05G0308
ATGGCGGCGGCGGCGGTAATTGCGGCAGTAGAGAAAAGCACTTCCAGGGATAGAAGCACT
TTACTGCATGGCAAATACGAGCTTGGGCGGCTACTCGGCCATGGCACCTTCGCGAAGGTG
TATCACGCGCGTCACTTGCAGACAGGAAGGAGCGTGGCAATGAAAGTTGTGGGGAAGGAG
AAGGTGATTAAAGTCGGGATGATGGAGCAGATCAAAAGAGAGATCTCCGTTATGAAGATG
GTGAAACACCGTAACATCGTTGAGTTACACGAAGTCATGGCGAGTAAATCGAAGATTTAC
TTTGCGATGGAGCTCGTGCGCGGCGGCGAGTTGTTTTCGAAGATCGCCAAAGGTCGATTA
AGAGAAGACGTGGCCAGAATGTATTTCCAGCAGTTAATCTCCGCCATCGATTTCTGTCAT
AGCCGCGGCGTTTACCACCGCGATTTGAAGCCGGAGAATCTCCTGCTAGACGAAGACGGT
AATCTGAAGGTAACTGATTTTGGATTAAGTGCTTTCTCCGAGCATCTAAAGCAGGATGGG
CTTTTGCATACGACTTGTGGAACGCCTGCTTACGTGGCGCCGGAGGTCATCAGCAAAAAT
GGTTACGACGGCGCCAAATCGGATATTTGGTCCTGCGGCGTGATTCTTTACGTTCTCCTC
GCCGGTTTTTTGCCGTTTCAAGACGATAACATCGTATCGATGTATAGAAAGATTTATAGA
GGAGATTTCAAGTGTCCGCCGTGGTTTTCATCTGAAGCCCGGAGATTAATAACCAAACTC
TTGGACCCAAACCCGAATAGCCGAATCACCATATCCAAAATCATGGATTCCTCCTGGTTT
AAAAAACCCGTTCCGAGAAGTTTGAAATCGTCAGAACTTGACGAAAAAGCCATTCTACGA
CGAGATGTACGAGAAATCAAAACAGCCGGAAACTCTAAACGCGTTTCATATAATTTCATT
ATCGGAAGGGTTCGATCTGTCGCCGTTGTTCGAGGAGAAGAAGAAAGAGGAGAAAGAAGA
GATAAGATTCGCGACGATGAGGCCTGCGAGTAGCGTGATTTCTAGATTCGAGGAGGTGGC
TAAGTCGGTGCAGTTCAATGTGAAGAAGAGCGATACAAGAGTGAGATTACAAGGTAAAGA
GAGCGGTAGAAAAGGGAAGCTGGCAATAAATGTCGATATATTCGCCGTAACGCCATCGTT

CATGGTGGTGGAAGTAAAGAAGGATAACGGTGACACTTTGGAGTATAACCAGTTCTGCAG
TAAAGAACTGCGGCCCGCACTTAAGGATATTGTCTGGATGTCAGCGACCGAGAATTCCAC
CATTAATGCTTAA
8>NISI05G0518
ATGGGGTTTGCAAACATCATAGGGAAGTATCATCTAGGCAGAACGATCGGGGAAGGCAGT
TTTGCCAAAGTGAAGCTGGGAGTAGATACAACAAATGGTCAATATGTTGCAGTCAAGATT
TTAGATAAGAGAATGGTCATGGAAACCAATCTCAAGAATCAGGCAAATTTCCGACTTCTT
TTTTTTTTTTTTTTCTCCGTTCTCAGTTATAGCAATAGATGTTTCACTCTTTCTAATAGC
ACTACGGAAACAGGTACAAAGAGAGATAAGAATGATGAAGCTTCTACGTCATCCAAACAT
TGTACGTATAAACGAGGTATGTTTGCTCTCTTCAGTTTCATTTTAAACCACTGTAAGTCT
GGGTTTTTTCCATATTTCATTCACATCAAGTTTTCACTACAGGTTATTGGCACAAAGACG
AAGATATATATAATAATGGAATATGTATCTGGAGGACAACTCTCGGATAAGCTGGTAAGA
TTCAGTGATTCATTGCATCTAGGGTTTCAGTGATAAGAGAATGTTAATGATCTCCAATAG
AGCAAGAAAGCTTTTTTAATTTGCATTTCCAATATAATCTTTCAGTTTTTCTATCTGTCT
AGTCTTATCTCAAGGAAATGAGTGAAGCAGAAGCAAGAAAGGTCTTCCAGCAATTGATCG
ACGTGGTTGACTATTGCCATAACAGAGGAGTTTACCACAGAGATCTAAAGGTCTTCTTAT
CAACTGACTCTTCTCTCTAACAATCAGATCTACAGCTTAATTTAACTTTGGTTAACTCAA
AACTATATGTATGATCATTCTTAGAAATTTATTTTTTATTTTTTATTTTTAGTTTTTATG
TTTAACTTCGATCACTATAGATCTAAAATTTAAAAATGGCCACTTTCGTCCAATTCAATC
TTAAAGAAAAAAGTTTTATAGGAGTAAAATAGTAATACTATCTATATAATTAAAAGTTTT
GTCCAAGGAAAAACCGGTGTAGTAGGTGAGATTTTTCCTCCAAGAAGGTCCTAAATTTGA
TCTTCCTTGGAGAATGGGGAGATTTTTAGTCTCCTCCACAGTGGAGGAGTACGGTAGTTG
TTTTTGTTCTTTTTATGGTTTTTAAGTTTTTATTAATTTTAAGCTCGATACCTTATTTGA
TTTTAGTATCTTCTATTAGAGTTCGTTATAAGGTTGAAAATGCAACGTTAGCTTAAAAAA
AAAAAAAAAAAATTGAAATAAAAGAAAATTTTTGTCCAAGAAGAACCGATAGAGTAGGTG
AGACTCTCCTTCAAGAAGGTCCAAATTCGATCCTCCTTGAAAAATGGGGAGATTGCTGGT
CTTTTTCACAATGAAGGAAGACATTAGTTGTTTCTTTATATCGTTTTTAAGTTTTTATTT
CAAACTTTAAGCTTGATACCTTATTTGATTTTAGTATCTTCTATATTAGATTTCGTTATA
AAGTTAAAAGTGTCATGTCAGCTAAAAATGAAAAAAAAAAAAAAAAATTGAAGTAAAAGAC
TAATTTACCCCTCATATCTTCTTCTTCAACATAACCCTAGACTACTCTCTCTTCTCTCAA
ACTGGCGGCCATCTGCTTCGCCTTCGCCGTCGCCGTTTCCGTTTCTGCTCAACACCACCG
TCGATTCTTCTTCCTTCCCTCTCTCCTTCCTCCCACTCGTCGCTCCCCTCTGGAAATCCA
GAATTTTATATCAAGGATACTTATACAAATACATCAGGATGCATTGTTTATTTGCCAACC
GCTTATACTTAACTCGTCTAGACCAGATATCGCATCAATTAATGTTTCTGGAAACACAAA
GATCAGTGACTTAAGAGCTTTTGCATCTACATGTAACTGCTTTGAATTGTTCATACTGTA
CATAGACCAGATATCGCATCAATTAATGGTTGTGTTTTGGCTGTTTCGATTACTTTTCTA
GAAAGTTTTCGGTTTTCGCGGACATTTTCCTGGAAAAATTTCTAGGTTTTCCGAGAAAAT
TTAAAATTTCTTAAAAATTTATTATTATTATTATTATTTCTTTCTTCTTTTCGCTCA
AATGTAAATGGAAACGGAAGAGGATGACGAGAAGAAGAAAATGAGAGCGCCGAAATTTTA
CTTAGATATTTTGGTGAAAAGTTTTTTATTTTTTTCAGAAATTTTTGGTTTTTTGGGAAA
ATTGTAAATTTTCCTGAAAAATTGAATATCCTTCTAAAATATTAAAACATACTAAAGTGT
AAACTTCCCTTCTTACCCTTATCATGTCAACCTTAACAGATTTTTTTAAACGAAAAGTAC
TAATTCAAGGGTTTTTCGAAATGAAATACCTAATTTAAAGTTAACAAAAAAAAAAAAAATG
ATACTATCTTCAAATTAGCTTTAATTAACAGATATCAAAATCAAATTATCCCTTTTTATT

ATAGTAATTTTTATAAAAATATTTTAATCATTTCACAATAAAAATTCGAATCTGTTATAA
TGTTTTGAATGAAAATGACTATTTTTTAAATTTTCGATCTAACATCTATCAAAGTTAAAA
GAATTTTTCTTAGAATGACTATATATATATATATATATATATATATATAGTTTTAAAGAC
CACGTTGGTGGCCGGCAAGTAAGATAAATGTTTATGATGCATATGCAGCCAGAAAACTTG
CTATTGGATGGTCAAGGAAATCTAAAAGTATCCGACTTTGGACTCAGTACTTTGCGGCAG
GTAGAAATACTTTTCTCATAATTTCCTTCTACTTACGTCTTATTCGTATCTAGGTATGTT
TTCAGAGGCCATATGATTTGCCATAGTTCTAGATCAGGAAGGGATTAACACTATATTTTA
TTACTGAGTTTAATGTATGCTGAACAGCCTGGAGATGTACTAACAACAGCCTGTGGCTCC
CCATGTTACGTGGCACCAGAGGTAACAAATTATTCTTCCCTACAGGGTTTCTTATACTTT
AGTCCCCAAGAAAAATTAAAAAAAAAAAAAAAAAAAAAAAAAAAGTTTTTGCTGTCTGAAAA
TTTATACTTTAGTTTAAGTTATAAGTTAAAACAACTTATCTATCATTGCATAGCTTAGCT
ATATACAATTGACTAATTTCATCAAGAAATTAAGAGATAAGGAGGAATAAGTAGAGAGAA
TTCGGCACTCGTATTTTTGAATAATGAAAGACTCTTCTTCCTTCTACGCGCTCCCCCTTT
TATAGCTATGAGATAAAAATTTATATTTTGGCCCTTCATCTCTATTAAATACCAATGGAC
CCCCAAACTATTAGTAATTACAAATGAACCCCAGAGCTATTAGAAATTACAAAGACCTGA
AACTATTAGTAATTACAAATGACTTCAAGAACCACTACAAATAAAACCAAGATTTAATGA
TTATAAGACTTTCTAAAAGAGTAAAGTGAAATTGGACTTAAGCAAAGTTTATATGTCTAA
ACCCAACATCATACAATCGGTATTTTTAGACGGGTTAATTTGACTTTGGTCTCTGTTAGA
TAGGTGTAATTAACTTTGATCTCTATTAGATAGGTTTGATTAACTTTGATCCCTATTAGA
TTGATAATATAACTTTAATCCCTGTTAGATTAATTTTGATCTCTATTAGATGAATAATAT
AACTTTAGTTCTTAAATCAGAACATAACTATAATTTTAATCCCTAAATCACATATAGTTT
TTGAAAAATTTTACTTCGACCCTTTTTTTTTATCTAATGTCATTTTAAGACTATTTTTAT
TTTTTTTGAACAAATGCATGATTTGGTAAAAACACCTTTTGTTAATTCAAAATAAATTTG
ATAATAATTTTATTATAAAAAAAAAAGGGATAATTACAATTTAATCCCCGTTATATAAAGC
TAATAAACATTAGTCCCTACTAGTGAGATAATGTGGTTTTAATCCAGAATTTAAAAATAA
TTAATATTGTTGTCCACGCTATTAGTCAAAATCTGTTAATTTTAATGATCAAAGGGTAAA
ATAGTAAAACTACCCTAAAATTTCTTTAATTTTTTTTAAAAAATAAAAAATTGAAATAAT
AATATTTTTAAAAGTTAATTAAAATAATAAACTAAATCCCTTTCTCCTTTCTCTCCTCTC
CCCGTTACCGAATTTCCATCACCGGTTGTCCTTCTCTTCTCAATTTTCCGTCACCGATTC
CATCCCCATCACCAGCACTCCTCCGTCGACCTTCTCTTCCCTTGTCGTCCCTGTCTCTTT
GTTTCCTCTTTCCAAAAGAACAATAAAAAAGAAAAGGAGATCTAGGAAGGGGAGAGAAAT
CGTAGATCTGAAACCCAAATCCTCTTCTCTTCTTTTTCTACCCAGATCAAAAAAAATGAA
AGAGAAAGATGGGGAAGATGAAGAAGATCCAGATCTACAATTTCTAGAAAGTATCGGTGA
CTGAAATAAAAAGCAAGAAGATGAAACCACTGCCACCACCCAGCCGAGGAGGAGAAACCG
GCGAAAGTAACTCAACTGAAGCCGAGAAGTCAAACCGGAAGTCAATGGTGAGAGATCCGA
CGACGATTTTCAGATCTTATCCTCTCTCTATCTTTCATCCTTTTTTTTTTTTTTTTTTTT
TCTCTTTGAATTTTTTTTTCTTTTTCAAATTGATGGTTGGCGATTTATGGATTTGGGTTGT
GTTGGATGTTGGCTGATTTGGACATGGTGGTAGGTGATGGGTGATTTGGGGGGAATGGTG
GTTAAGGTGGTGGCCGACAGTGGTGAATGAACAGTGATAGGCTTTTAAAAAAATAAAAAT
TTATTAAAAAATAATTAATAAAAATTATGGACTGTTAGATCTAATTGAGAAGTTGATCAG
ATGGTCTAAAAAAAAATGAGTAACTTTGCATCTATCATAAAGTGTAATTGTCTCTTTTTTA
ATTTTAAAAATTTACATAGATAAGGATAGTTCAGTAATTTCAACAAAATTTGTTGATGTC
ATTATTTTCTGTTAAAACGAACATTAACGGTAGGGACTCAGTTACATATTACTTTGCAAT
CGAGGAACTAAATTTTATTGCAGTGACTATTGGGGACCAATGTTTATTAACTCTATCTAA

CAGAGACTTCGGCCAAATTATCCCATAAAAAATATTATTTATTTATTTAAAAAATTAAAA
TTAAATTATCCCATAAAAAATATTATTTATTTATTTAAAAAATTAAAATTAAAAAAAAAA
AAAAAAGATCGACAGGAGACCAAGCGGTCTCCTCCATGATGTGCACTAGAGGAGACCAGA
TTTGGTCTCCTCCAGTGGAGGAGAGGAGATCTTCTCTCCGTCGGAGGAGACCAGATCTGG
TCTCCTTCAGCAGGGGAGGAGAACGTCGATCTCCTCCCACCATGGAGGAGACCACTTGGT
CTCCTCTGGGGTTTTTGTTTTTGTTTTTTTTTTTTAATTTTTTTTAATTTTAATTTTTTA
AATAAATAAATATTACTATATTAAAAATAAAATTATTATTAAATTTATTTTGAATTAACA
AAGGGTGTTTTGGTCAATTATCTCTTTTGAGGTTCCATTTTTTTAATTTTTTGGACCAAA
ACGTGCATTTGTAAAAACAATACTCTTAAAGTGACATTAAACAAAAAAACAGGGGTCAAA
GTAGAATTTTTCAAAAATTATATGTGATTCAAGGACTAAAATTATAGTTATTTTCTGATT
TAAAGACTAAAGTTATATTATCCATCTAATAGGGATCAAAATTAATCTAACAAGGACTTA
AGTTATATTACCCATCTAACATGGATTAAAGTTAATTAGACCTATTTAACATGGACTAAA
GTTAAATTATCTCTTTTTAGACATTAAAAAATTTGCAACGTTGATTTCAAATTCAGTAAA
AGAGTGCTTGTCTTTTTACTCTTGTATTTGTAGTAATATACTGAAAAACAGTTCTATAAT
AACATACTTCTTAATTTAACTGATAGCATAGCAGGGACTAAAGTATAATTTTCAAATTCC
AAAGACTAAAGTATAATTAAGCTTTTCCCATGGACTAAAGTGTAATAAACCCTAAAATCT
AACGGTTAGTTTTTATTCCTTAAAACATAAAAGCCAAAAGGAGAGAGGCAGGAAAAAGAA
AACAAGTAAAAATAATTTCTTAACAAAGATATCACACCTGAAATCTGCTTCAATCTCTGT
CCAACAGCTGCTTGCAAGTAGAGGCTATGAAGGAGCAGCTGCAGATGTTTGGTCTTGTGG
AGTAATCCTCTTTGAACTACTTGCTGGTTATCTGCCATTCAATGACCGTAACCTTATGGT
CTTGTATAGGAAGGTATGGCTTTAATCACAGTATGAAAAATTCTTTGTTGGCCCGATACA
TCTCTTTTCACTGATAACTGTTCTACAAAAAGATAGCAGGAGCAGAATACAGATTTCCAC
ATTGGTTTACAGAAAGCCAGAAGAATCTAATCTCCAGGATACTTAATCCAAATCCTAAGA
AGGTATAAACTCCAAACCATTCTACATATCTTAACACATTTTTGTAATGGACATAACTAT
TTCTTTGTGCATTGCGACAGAGAACGACAATACAAGAGATCATTGAGGATAAATGGTTTC
AAACAGATTATGAACCTTCTTGTGGACGTGAATACGATGAGAAAATCTACTTGGACGATA
TTTATGCCGCTTTTGCAGTCAAACGAGGTAAATGAAAAGTATTAACTAGCAGAAAAAACA
GTTTTTAAACCATCACCTAATGACCTGAATTTGCAGCAGGAGAATGGTATGTCAAAATCG
TCAAGTTTTATAAATGCATTCCAGTTAATAGCAATGTCACAAGACCTAGATTTGTCGGGA
CTGTTTGAAGGGCATGTAAGTATATAAAACAATATCTCTCTGGTTTTACCTCTCGACAGG
CTTCTTTTGCACAATGGTACTGCTGTTTGCTGTACTATTATTGACCTATGTTCTTTTGAA
AAATATCACAACATAGTTTCCAAAATATCACAAAAGACCTCATTAATTTGCTTAATTTTT
CATAAAACCATGCAATTTGAAGATGTATACGAAATTATATAGAAAACATGACATTTCCTA
ATATTCTTAAAACTAAGTGGAGCATTGTAATGTTTTTCAAACAAATAGGAACCAAACGAT
ATCGTGTGAATGAACAAAGATGTTTTTGAAATTAACCGTAGTTTTAACATAGCCTTTCCT
ACAGGATGACAAGAAGGAGAAAACAAGGCTTGGATCCAAGTTTCCAGTCAACGAAACAAT
AAAGAAAATAGAAGCTGCAGCAATGGATGTGAGTCTAATGGTTGAGAGGACAAACAGCTT
TAAAGTAAGGTCTTCTCGTATTCTAGATACTTGAAGAAAGTGGATTTTTTAGTTCCAGTG
CAATTAACAGTGTCCTATTTAATGCTCAGATGAAAATTCATCCAAAACAGAAGCAGAAGA
TGAGTAGATGCGCAAGATCATATTATGACCTCTCAGCAGAGGTAAGCAAATGTGATATAT
ATGCTCTTCCTGTATTTTGAATGTCAAATTCTCACCATCATGTCTCTTAAATGTCAGGTT
ATTGAGGTGGCTCCAACTAATTGTGTCATAGAAATATCAAAGTCCGCAGGAGAGCTAACT
GTATTCAAAGAGGTACTATAGGATTTCTATGATTTGGTTACTTGGGTAAATTACAGAAAA
ATCTACTGACTTAGAATTATGTTGTTTATCAGTTTTGCAGAAGTTTATCAAGTCTGCTAA

CAGAAAAACCCAATACGTCAAATCAAACACAAGAATTCAGTATAGGCAGCGTCAATACAA
GAAGTACACCAAAAATTGA
9>NISI09G0114
AGTTAATGTTTTGCATCAGCCACCAGAGGAAGGGGAACAAATTAAGAAGATGGAAATGGT
CAAATGGAATGAGAAGTAGAAGCAGCTTAGCTGGCTGGTTAGTGATTTCAGTGTATTTAC
TTATATATATATATATATATATATATATATATATATATATATATATATATATCCATTTTTAC
CATTCTTATCATCATCATATTCATCATCTGTCTTATTGCAAGATTCAAGTATATCCTCTC
TCTCTCTCTCCCTCCCTCCCTCCCTCTATCAATTATTTGTTTTAATTCTTTTCCTTTTCT
TATTGTAAATGAATAACTAAAATTGCTTGAGCCTACGCATAAATTGCTTGTTTGTCTTTT
GAATTTTGGTTCCCCTTCCCTTTCCACTGTCTGCTCTGCCTCTGCTGCATATCTTTCTCT
TTTGGTGCTCTGCAAGAGGTGAATTTTGCATTTCCACAACTCAATTGAAGCTCTTTTCAC
CATTTTTATTTATTTATTTATTCATTTTGATAAAGACTGCAGTAGGGGAGGCATGGGTCG
GGTTGTTTTGAGTTAAAAACAATACTTCTTCCTTTCACTAAATAATTTATTCCATCAATT
CTTCAGTTTATGAATTAATATATCAAGCATGGTGAACTAGGAATAGTAGCAAATTCAATT
GCTTAGAATTTCCAAACCAAACCTTCTTTCTTCTTGGTCGGAAATTTAAGTTTTGATTTG
TGGACTTCTGACAAGTTTTTCTTATTTAAATTTGTTGAGATCTCATCATTGGTTTTATTG
GTATGAACATGAAACAACATTGATTTTAATTATATGTTTCTGTACTTACTAATCTCTACC
CTTTGTTTTCTATGTGAAATGGGCAATAATGTTAGCTTTGGTTAAAGCTTAAACTGGAAT
TTCAATCCTCTAATCCTCTTTTTTGCCTTAATTAACAGCTTTCTTATATCAAGGTAGAGA
TTCGTCCCCCTTGAAGTCCTGTCTTTGGACCTGTTTCTTTTATTAAGAAGTTTGCTGTTT
GGGTTAAAAATTGTATTTTTTACCATGTTTGAGTTGACACATTCAAATCAATTTATAATC
ATATATTCCTGCTTGCTTATTTCATATTTGCTAATTAAAATGTGAGTATTACAGTTGCTT
GGTTCTGGTGAGACTTCTTTATTTGGGGCCGCAGAGTTGAAAGCCTATTGAAGTTACAGA
GGACAAGTTTTTTGTGTAAAGGAATAATGAGTCAGCCTAAAATAAAGCGTAGGGTGGGTA
AATACGAGGTTGGCAGAACCATAGGCGAAGGAACGTTTGCAAAGGTGAGGTTTGCTAGGA
ATTCTGAGACTGGGGAAGCCGTAGCTCTCAAGATTCTTGATAAAGAGAAGGTTCTTAAGC
ACAAGATGGCTGAACAGGTCTACCTTTCATTCTCTTTAGTCCTTTTATTCATCATGGTCT
CTTTCTTTAGCTAAGAATCACATACTAGTTCATGGTGTACCAAGTTAGCTTAAGCTTTTG
AGAGAATTACTTTCAAACAATTTCGGCTGATGATAACTGATGTAGTGTAGTTACTTATTA
ATACAGTAGTCTGTAGCCGTTGAGACTTCTATTTCTATTATTCATGAAAGAAACGGTGTT
CTTGGTTTATGAACTTTTTTTATGTGCTTGCTTTTCTCTAGATCAAGCGGGAAATTGCAA
CGATGAAGTTAGTAAAGCACCCAAATGTTGTTCAGTTGTATGAGGTCACTCTCTCTCTCC
TTGAAAAGCATGTGTTCTCTTTTTCCTTTTTTATTGTGCTTTGCATCCAGGCAATATATC
TTACCCTTTGGTTCAGGTGATGGCAAGCAAAACGAAGATATTCATAGTGTTGGAGTTTGT
CACTGGAGGAGAGCTCTTTGACAAAATTGTGAGTAGCCAACTCTTGTAGTCTTCTTAAGT
GCGTTTGGCAGTACTTCTAATTTTAGAAGTGCTTTACTAATTGAAAACGGATACTTGGAT
GTTGCATTGAGAAACATTTTGTTATATTTTAGAAACACGAGGGGAGGTGCTTCTTCAAGA
ATTGCTTCGAATAAACCCCCTTTAGTAAAAAAAACACTCTCAAGTGGACCTAATGTTAAG
AGGTTACTAACTAATCTTATAAAAGATTGGGTGTCCTCAAGGAGTTTGACTATTCCCCCA
TATAGCTTTACTTTACTCATAAACCACTTCTCTATGTCGTTATAATTAGAATGGATCATG
GTTGCTTGTATAAGTTAACACCTATGATTTGTAATGCATCGGTATTAATGGGACTTCTGA
TCTTATTTTGATCACTCATGTGCACTGGTGGATGTGCTTATTTTGATATATTGGATTGTG
AAAAATGAAGGTAAACCATGGACGGATGAGAGAAGATGAGGCACGTAGATATTTCCAGCA
GCTTATAAATGCCGTTGATTATTGCCATAGCCGAGGTGTTTATCATAGAGACCTCAAGGT

ATTACACCCCCTAAAATACAGGTTATACCTTTTCTTCTATGTTATTGGATTTTTTATCAT
AAGTTCTTTTTTCTGTTCCTCATTATGAGTTTTTGCTGCAGCCGGAGAATCTGCTTTTGG
ATGCCTATGGAAACCTTAAAGTTTCTGATTTCGGATTGAGCGCTTTGTCTCAACAATTGA
GGGTAATTATGATGTTATGGGTTTGTTTACTTTGTGATCGTTCTGGTCTTGGCATATGGC
TTTTTTTTTTAGTGCTTATTGACTAAAAGGTTTTGCTTACATTGCTTCTCAAATCTTCAC
CAAGAATTACTTGAAATGCTTCAAAATATAATAAAGCGTTTCTCACATTCAAATACTCTT
GTTTTATCAAGCAAAATGCTTCTAGAAGCCAGAAGCTACATCAATGCACCCAAAATTCTC
TGCAAATTATTAGCAGGAAGAAGAGTAATATAAAATGAGAAAGCATGCATCTAAATGTTA
ATTATTTTGGAATACCTGTATTATTTAAGTTAATGTTATCTCTGCTATTATACTCTTTAA
GCATCTCAGATGATTATATTCTTTATCAAGGAAGAATATTTTTCTCAGTCAAAATATTTT
TCTTAGTCAAACTCACTTTTAATCAATTATCCTCATGCGGCCCACTATATTCATACAATC
AAATCCATAGATTTTGTTTGGCTTAAATGATCCTGAAACTGGTATTATGGAAGACATTAC
TAACTTAGCTAATATAAGAGGCAGAGGAAAACTGGCCTCCCTCATTCTTATGACTATACG
AAAGACTATGTCCTACTTAAGGATTTTTGTTTATTATTATTTTTATTGTATATGTATATA
TCTTAATCTCATTGTTAATGTTGGCAGAATGATGGCCTTCTCCACACAACCTGTGGAACT
CCAAACTACGTTGCTCCAGAGGTATGAGCTGAGGCTGAAAGGATTTCAGCTTTTAACACA
TATGTAACCTTTTCTAGCACTTTGGATGACAAAAATGTTTGTATATCCTTAGGTTCTGAA
TGATAGAGGATATGATGGGGCAACCGCAGATTTGTGGTCGTGTGGAGTCATACTCTTTGT
ACTGCTTGCTGGGTACTTGCCTTTTGATGATAATAATCTTATGACCCTTTATAAAAAAGT
GAGCACTTTTTCGTACTTGTAATGTAATTCTCTCCTTTTTTGCAGAAGCTGAAAATCACT
CTAAACTTTTCCTTTCTTAATGTGTAGATCTCTGCTGCTGAATTCACATGCCCCTCATGG
CTCTCTCTTCCTGCCATGAAATTGATATCTCGTATCTTGGATCCCAACCCCGTGACAGTA
AGTATCACTCTGTAGAACTTCTGGCTTAAAATTTCCCATCATGACACTTCTATTTGTCCA
TCTCCTAACATTTCCGAGAATAAACTTCTGGTGTTGTCTGTATTGACTTCAGATTATTAA
GATATCATTACTAACTACAAAAAAGGTCGAACACATACATGATACTAATGTCCATGTTTG
TATTTTATCATGACGTGTCCAGTAATAATATCATACTTGCCAAATGAGTTAGCCATATTG
CTGCTTCCAAAAGAAAGCCATGATGAACATGTATTATGATTTGTAAATGTACATTGAACC
GGAATTTTAATGAAAGAGTTTATCATTAGTTGGGGCCTTCAGTTTTGATTTATTTGGAGG
GCTTTATATTTTATTTAATTCAAACCTTTAAATTTAATTCACATGGAGTGCAAAATGCCT
CCTCTTTTCGTCATTGATGTTGTGTGCATCCACATGAACAAATATCCAGTTAGAGTTTTC
CTTTTGCTTCTAGGTCAAAGATTTCTTTGCAGCTTGAATAGATAACCATCTAGTATATTA
ATTATATTGAAACCATTAAAATGATAATTGATTAGAATTTGTACATGCACTCCTCGAACA
GTTTAACTTGGAAGTGAAATATTGAAGACGATCTTATGTCTCTTTTTATGTTTCTTTTTT
CCATAATATGCTTATATCATTTCTTTTTCTGGATAAACACACTATTGGAGATACAATCTA
AGAACCCGGTATCAAAATGTATCATTCTTTCTGTTTCTTTATTCTCCCAACTATTCTCAG
GAAACATGCGTAGAGTTGTTGTTGGTCTAGTTAGTCGCCGAGCATCGCAAACACCATTCC
TTCAGGAGAAAAAAAAAAATGAAGAAGAAAAGATTTTTTATTTAAATCTTCTTCTTGGTCT
CTTTAAGATTTGTTGCACTGTCTCAGTTAGTTGACATATTTTTCTCTCTCTGGTTTTCTG
TGGCAGCGAATTTCTATTGCGGAAATTCTGGAAGATGAATGGTTTAAGACAGATTATAAA
ACCCCAGTGTTCGTAGAGAAAGAACATGCCAATGTGGATGATGTTGAAGCTGTTTTTAAG
GATTCGGAAGTAAGTTGGATGAGTTTTAAGTAAACAAACAATAATAGAATGAAGCATCAC
ATATAATATGCACTTGTAAAAAATTGTCTAAGCAATTGTGTGCTATATCGTAACAGGAGC
ATCATGTAACAGAGAGGAAAGAAGAACAGCCAGTGGCAATGAATGCCTTCGATTTGATTT
CTTTGTCAAAGGGTCTAAACCTTGGCAATCTGTTTGATGTAGCACAGGTATTATTGTTAA

CCCCTTATGCTCTGATCAACTTTCAAATCGGTACATATGCCATTGTCTCTATTTAGGTTT
TTCTTTTCCCCCTCAATTATATATAATTTAAACCCCAATCCTGGCCCACCCACCAAATGC
AAATGAAACAGAAATTAGATTAGATAACCTTGCACCAAGGTGGTTTGGTAGGAGTCGAAC
TTCAGACCTCTTATCTCCATTTGGTTTGAATTAATGGCTGAATGAGAGACCTTTATTCCT
CCTGTCTCTCTCAACCCTAATTTTTCAATTCCCTCTCCCTTCTGCTCATATAGTCATATT
ATCCGGTCGTAGTTTTGTAATCAGAAAATAGACTAGATGGCTCGGAAACCTGGCTGGATC
AGTTAAACCTGACCTGGCCGGAGCTACTCCAAATCACGCTAAGGGACCTTCATTTCACTG
CTTTATCACCTAGGAAAAATGGTAACCTGGATCCTGTCAATAGTGACATTGTTCTTCTTT
TCCAATTGCTGTAATGTCCCTCATGAAACCTTGATTATTTCAAACTAGTTTGTTTTGTTT
GTCGGCGGTAGAAGACTGTGAAATATTAAATCAAATTTCGTATTATTGGTAAACACTTTG
TGGATTGCAGGGATTCAAGAGGGAAACAAGGTTCACATCCAAACGTCCAGCTAATGAGAT
AATCAGTAAAATTGAAGAAGCGGCTAAGCCTCTTGGGTTTGATGTTCATAAGAAAAACTA
CAAGGTGAGGGAATTCATTCTTCACATATTCAACTTTGATGATCAAGTAAATTTTTTTTA
ATTTTTATCTTTTTGAAAATTAATTATTATTGAACTCAGATGAGGCTTCAAAATGTGAAA
GCGGGAAGAAAGGGAAATCTTAATATAGCGACAGAGGTAAGGAGGAACAACTCCCACACC
TTTCATATTTATATGTAACTGATGGACTTTGTGCTTATTATATTATAATATATAAACCAT
AGTTCAACCTCCTAACAGCTTAAGCTTTTGGGAGGATTGGTTTTCAAACATGGTATCAAA
ACCAAGTTTGAACCATGGCTATCCTTCAAAATATAATTAAATTATGTAGGGCTACAGAAT
GAGTCTATGAATAGGTCTTGCATGTTAAAGGACGTGTTAAGAGTATAGTATGTATGTTAG
CTTAAGCCTGCGTTTGAGAGTGTTGGTTTGAAAAAGTGTTAATATGAGAAATGTCCTAAC
AGATATTTCAAGTGGCACCCTCTCTTCATATGGTCGAGGTGAGAAAGGCAAAAGGCGACA
CATTGGAGTTTCATAAGGTATCATTTTTTTTATTTTCTTTTTTTCCTTTTTCAGTTTTAG
TTGTATTTTCAAAAATGTTTCATCATGATGGCCAGTTTTGATGAATTGGATTCTGTATGT
ATGTGGGGTGCAGTTCTATAAGAATCTGTCAAGCAGACTGGAGGGAGTAGTGTGGAAAAC
AGAAGAGCAAATGCAAGAAATGGAGTAAAAAAGTAGTAATTATAATGTTGGTGAACAAGG
TTGTTTGTGGTGTATATCTTCTTCATTCATTCTTCTCTCTGTAGAATTCTTTAGTTTTTT
TCCAACTTTCCATGTTATTATTGTTGGAAAGCATCAAGCATGGCTCCTGTAAAGGTTGAA
TTCTTTGTTCTTCTCTTTCTTCTGCTTTGAAATTCAATATTTTGGTGTCTCTCTCTCTCT
CTCTC
10>NISI09G0120
TTGCTTGGTTCTGGTGAGACTTCTTTATTTGGGGCCTGAAGTTACAGAGGACAAGTTTTT
TGTGTAAAGGAATAATGAGTCAGCCTAAAATAAAGCGTAGGGTGGGTAAATACGAGGTTG
GCAGAACCATAGGCGAAGGAACGTTTGCAAAGGTGAGGTTTGCTAGGAATTCTGAGACTG
GGGAAGCCGTAGCTCTCAAGATTCTTGATAAAGAGAAGGTTCTTAAGCACAAGATGGCTG
AACAGGTCTACCTTTCATTCTCTTTAGTCCTTTTATTCATCATGGTCTCTTTCTTTAGCT
AAGAATCACATACTAGTTCATGGTGTACCAAGTTAGCTTAAGCTTTTGAGAGAATTACTT
TCAAACAATTTCGGCTGATGATAACTGATGTAGTGAAGTTACTTATTAATACAGTAGTCT
GTAGCCGTTGAGACTTCTATTTCTATTATTCATGAAAGAAACGGTGTTCTTGGTTTATGA
ACTTTTTTTATGTGCTTGCTTTTCTCTAGATCAAGCGGGAAATTGCAACGATGAAGTTAG
TAAAGCACCCAAATGTTGTTCAGTTGTATGAGGTCACTCTCTCTCTCCTTGAAAAGCATG
TGTTCTCTTTTTCTTTTTTTATTGTGCTTTGCATCCAGGCAATATATCTTACCCTTTGGT
TCAGGTGATGGCAAGCAAAACGAAGATATTCATAGTGTTGGAGTTTGTCACTGGAGGAGA
GCTCTTCGACAAAATTGTGAGTAGCCAACTCTTGTAGTCTTCTTAAGTGCGTTTGGCAGT
ACTTCTAATTTTAGAAGTGCTTTACTAATTGAAAACGGATACTTGGATGTTGCATTGAGA

AACATTTTGTTATATTTTAGAAACACGAGGGGAGGTGCTTCTTCAAGAATTGCTTCGAAT
AAACCCCCTTTAGTAAAAAAAACACTCTCAAGTGGACCTAATGTTAAGAGGTTACTAACT
AATCTTATAAAAGATTGGGTGTCCTCAAGGAGTTTGACTATTCCCCCATATAGCTTTACT
TTACTCATAAACCACTTCTCTATGTCTTTATTGTTGTAGTTATAATTAGAATGGATCATG
GTTGCTTGTATAAGTTAACACCTATGATTTGTAATGCATCGGTATTAATGAGACTTCTGA
TCTTATTTTGATCACTCATGTGCACTGGTGGATGTGCTTATTTTGATATATTGGATTGTG
AAAAATGAAGGTAAACCATGGACGGATGAGAGAAGATGAGGCACGTAGATATTTCCAGCA
GCTTATAAATGCCGTTGATTATTGCCATAGCCGAGGTGTTTACCATAGAGACCTCAAGGT
ATTACACCCCCTAAAATACAGGTTATACCTTTTCTTCTATGTTATTGGATTTTTTATCAT
AAGTTCTTTTTTCTGTTCCTCATTATGAGTTTTTGCTGCAGCCGGAGAATCTGCTTTTGG
ATGCCTATGGAAACCTTAAAGTTTCTGATTTCGGATTGAGTGCTTTGTCTCAACAATTGA
GGGTAATTATGATGTTATGGGTTTGTTTACTTTGTGATCGTTCTGGTCTTGGCATATGGC
TTTTTTTTTTAGTGCTTATTGACTAAAAGGTTTTGCTTACATTGCTTCTCAAATCTTCAC
CAAGAATTACTTGAAATGCTTCAAAATATAATAAAGCGTTTCTCACATTCAAATACTCTT
TTTTTATCAAGCAAAATGCTTCTAGAAGCCAGAAGCTACATCAATGCACCCAAAATTCTC
TGCAAATTATTAGCTGGAAGAAGAGTAATATAAAATGAGAAAGCATGCATCTAAATGTTA
ATTATTTTGGAATACCTGTATTATTTAAGTTAATGTTATCTCTGCTATTATACTCTTTAA
GCATCTCAGATGATTATATTCTTTATCAAGGAAGAATATTTTTCTCAGTCAAAATATTTT
TCTTAGTCAAACTCACTTTTAATCAATTATCTTCATGCGGCCCACTATATTCATACAATC
AAATCCATAGATTTTGTTTGGCTTAAATGATCCTGAAACTGGTATTATGGAAGACATTAC
TAACTTAGCTAATATAAGAGGCAGAGGAAAACTGGCCTCCCTCATTCTTATGACTATACG
AAAGACTATGTCCTACTTAAGGATTTTTGTTTATTATTATTTTTATTGTATATGTATATA
TCTTAATCTCATTGTTAATGTTGGCAGAATGATGGCCTTCTCCACACAACCTGTGGAACT
CCAAACTACGTTGCTCCAGAGGTATGAGCTGAGGCTGAAAGGATTTCAGCTTTTAACACA
TATGTAACCTTTTCTAGCACTTTGGATGACAAAAATGTTTGTATATCCTTAGGTTCTGAA
TGATAGAGGATATGATGGGGCAACCGCAGATTTGTGGTCGTGTGGAGTCATACTCTTTGT
ACTGCTTGCTGGGTACTTGCCTTTTGATGATAATAATCTTATGACCCTTTATAAAAAAGT
GAGCACTTTTTCGTACTTGTAATGTAATTCTCTCCTTTTTTGCAGAAGCTGAAAATCACT
CTAAACTTTTCCTTTCTTAATGTGTAGATCTCTGCTGCTGAATTCACATGCCCCTCATGG
CTCTCTCTTCCTGCCATGAAATTGATATCTCGTATCTTGGATCCCAACCCCGTGACTGTA
AGTATCACTCTGTAGAACATCTCCTAACATTTCCGAGAATAAACTTCTGGTGTTGTCTGT
ATTGACTTCAGATTATTAAGATATCATTACTAACTACAAAAAAGGTCGAACACATACATG
ATACTAATGTCCATGTTTGTATTTTATCATGACGTGTCCACTAATAATATCATACTTGCC
AAATGAGTTAGCCATATTGCTGCTGCCAAAAGAAAGCCATGATGAACATGTATTATGATT
TGTAAATGTACATTGAACCAGAATTTTAATGAAAGAGTTTATCATTAGTTGGGGCCTTCA
GTTTTGATTTATTTGGAGGGCTTTATATTTTATTTAATTCAAACCTTTAAATTTAATTCA
CATGGAGTGCAAAATGCCTCCTCTTTTCGTCATTGATGTTGTGTGCATCCACATGAACAA
ATATCCAGTTAGAGTTTTCCTTTTGCTTCTAGGTCAAAGATTTCTTTGCAGCTTGAATAG
ATAACCGTCTAGTATATTAATTATATTGAAACCATTAAAATGATAATTGATTAGAATTTG
TACATGCACTCCTCGAACAGTTTAACTTGGAAGTGAAATATTGAAGACGATCTTATGTCT
CTTTTTATGTTTCTTTTTTCCATAATATGCTTATATCATTTCTTTTTCTGGATAAACACA
CTATTGGAGATACAATCTAAGAACCCGGTATCAAAATGTATCATTCTTTCTGTTTCTTTA
TTCTCCCAACTATTCTCAGGAAACATGCGTAGAGTTGTTGTTGGTCTAGTTAGTCGCCGA
GCATCGCAAACACCATTCCTTCAGGAGAAAAAAAAAATGAAGAAGAAGAAAAGCTTTTTTA

TTTAAATCTTCTTCTTGGTCTCTTTAAGATTTGTTGCACTGTCTCAGTTAGTTGACATAT
TTTTCTCTCTCTGGTTTTCTGTGGCAGCGAATTTCTATTGCGGAAATTCTCGAGGATGAA
TGGTTTAAGACAGATTATAAAACCCCAGTGTTCGTAGAGAAAGAACATGCCAATGTGGAT
GATGTTGAAGCTGTTTTTAAGGATTCGGAAGTAAGTTGGATGAGTTTTAAGTAAACAAAC
AATAATAGAATGAAGCATCACATATAATATGCACTTGTAAAAAATTGTCTAAGCAATTGT
GTGCTATATCGTAACAGGAGCATCATGTAACAGAGAGGAAAGAAGAACAGCCAGTGGCAA
TGAATGCCTTCGATTTGATTTCTTTGTCAAAGGGTCTAAACCTTGGCAATCTGTTTGATG
TAGCACAGGTATTATTGTTAACCCCTTATGCTCTGATCAACTTTCAAATCAGTACATATG
CCATTGTCTCTATTTAGGTTTTTCTTTTCCCCCTCAATTATATATAATTTAAACCCCAAT
CCTGGCCCACCCACCAAATGCAAATGAAACAGAAATTAGATTAGATAACCTTGCACCAAG
GTGGTTTGGTAGGAGTCGAACTTCAGACCTCTTATCTCCATTTGGTTTGAATTAATGGCT
GAATGAGAGACCTTTATACCTCCTGTCTCTCTCAACCCTAATTTTTTAATTCCCTCTCCC
TTCTGCTCATATAGTCATATTATCCGGTCGTAGTTTTGTAATCAGAAAATAGACTAGATG
GCTCGGAAACCTGGCTGGATCAGTTAAACCTGACCTGGCCGGAGCTACTCCAAATCACCC
TAAGGGACCTTCATTTCACTGTTTTATCACCTAGGAAAAATGGTAACCTGGATCCTGTCA
ATAGTGACATTGTTCTTCTTTTCCAACTGCTGTAATGTCCCTCATGAAACCTTGATTATT
TCAAACTAGTTTGTTTTGTTTGTCGGCGGTAGAAGACTGTGAAATATTAAATCAAATTTC
GTATTATTGGTAAACACTTTGTGGATTGCAGGGATTCAAGAGGGAAACAAGGTTCACATC
CAAACGTCCAGCTAATGAGATAATCAGTAAAATTGAAGAAGCGGCTAAGCCTCTTGGGTT
TGATGTTCATAAGAAAAACTACAAGGTGAGGGAATTCATTCTTCACATATTCAACTTTGA
TGATCAAGTAAATTTTTTTTTAATTTTTATCTTTTTGAAAATTAATTATTATTGAACTCA
GATGAGGCTTCAAAATGTGAAAGCGGGAAGAAAGGGAAATCTTAATATAGCGACAGAGGT
AAGGAGGAACAACTCCCACACCTTTCATATTTATATGTAACTGATGGACTTTGTGCTTAT
TATATTATAATATATAAACCATAGTTCAACCTCCTAACAGCTTAAGCTTTTGGGAGGATT
GGTTTTCAAACATGGTATCAAAACCAAGTTTGAACCGTGGCTATCCTTCAAAATATAATT
AAATTATGTAGGGCTACAGAATGAGTCTATGAACAGGTCTTGCATGTTAAAGGACGTGTT
AAGAGTATAGTATGTATGTTAGCTTAAGCCTGCGTTTGAGAGTGTTGGTTTGAAAAAGTG
TTAATATGAGAAATGTCCTCACAGATATTTCAAGTGGCACCCTCTCTTCATATGGTCGAG
GTGAGAAAGGCAAAAGGCGACACATTGGAGTTTCATAAGGTATCATTTTTTTTATTTTCT
TTTTTTCCTTTTTCAGTTTTAGTTGTATTTTCAAAAATGTTTCATCATGATGGAGAGTTT
TGATGAATTGGATTCTGTATGTATGTGGGGTGCAGTTCTATAAGAATCTGTCAAGCAGAC
TGGAGGGAGTAGTGTGGAAAACAGAAGAGCAAATGCAAGAAATGGAGTAAAAAAGTAGTA
ATTATAATGTTGGTGAACAAGGTTGTTTGTGGTGTATATCTTCTTCATTCATTCTTCTCT
CTGTAGAATTCTTTAGTTTTTTTCCAACTTTCCATGTTATTATTGTTGGAAAGCATCAAG
CATGGCTCCTGTAAAGGTTGAATTCTTTGTTCTTCTCTTTCTTCTGCTTTGAAATTCAAT
GTTTTGGTGTCTCTCTCTCTCTCTCTC
11>NISI10G0902
ATGGCAAGCTCGGCCGCCGCGAGCGCCAACGTCGGTGGCAAAGAGCAGCAGAACCAGAGC
CCGCTTCTCGGCCGCTATGAGGTCGGGAGACTTCTAGGCCATGGGACTTTTGCGAAAGTC
TACCAGGCTAAGAACGTCAAGACGGGAGAAGGCGTCGCGATCAAGGTGATCGACAAGGAA
AAGATATTGAAGAGTGGACTAATCGCTCATATCAAGCGGGAGATCTCAATTCTGCGTATG
GTACGCCATCCGAACATCGTGCAGCTCTTCGAGGTTATGGCGACCAAGTCGAAGATCTAC
TTCGTCATGGAGTATGTTCGCGGCGGTGAACTTTTTAACAAAGTCGCCAAAGGAAGATTA
AAGGAAGATCTCGCGAGGAAATACTTCCAGCAGCTAATTTCGGCAGTAGGGCTTTGCCAT

GCGCGTGGAGTCTACCACCGCGACCTGAAACCTGAAAATTTGCTGCTGGACGATAACGGC
GATTTGAAGGTTTCCGACTTCGGACTTAGTGCTGTGTCCGATCAGATTCGGCAAGACGGT
TTGTTTCATACTTTTTGTGGAACTCCGGCGTATGTTGCTCCGGAGGTTTTAGCTAGGAAA
GGATACTGTGCGGCCAAAGTAGACATTTGGGCGTGCGGTGTCATATTGTTTGTTTTAATG
GCCGGATATTTACCGTTTCACGACCAAAACATAATGTCTATGTATAAAAAGATATATAAG
GGTGAATTTAGGTGCCCTAAATGGTTTTCGCCAGATTTAGTCAGGCTTATGAAGAGGCTT
CTCGCTACAAACCCTGATACTAGAATTACCATTCCTGAAATTATGGAGAATAGGTGGTTT
AAAAAAGGGTTTAAGCATATTAAATTCTATATAGAGGATGACAAGCTCTGTAATGTTGTT
GATGATGATGCCGATGTGGAGTCATTATCTGAGCAGTCAATGTCTGAATCAGATTCTGAA
TTGGAGACCAGAAGGAAAGTTACTTCTTTGCCCAGACCGGCAAGTTTGAATGCATTTGAT
ATTATATCGTTTTCTCCTGGGTTTGATTTATCGGGACTATTTGAAGAAGGAGGAGGAGCA
AGATACGTGTCTGGTGCTCCAGTTTCAAAAATTATATCAAAATTGGAGGAGATTGCTAAG
GTGGTGAGTTTTACAGTGAGGAAGAAGGATTGTAGAGTGAGTTTGGAAGGGTCTAGGGAA
GGTACGAAGGGGCCATTAACTATAGCGGCTGAAATTTTTGAGTTAACATCTAAGCTGGTG
ATGGTGGAGGTGACCAAAAAAGGAGGGGATAGAGGTGAGTATGAGGAGTTTTGTAATAAG
GAATTGAAACCTGGGTTGGATAATTTAATGGCCGAGGAATCAGAACCTGCTGCTGTTGTT
CCTGCTGCTGCAGCTGATTTGCATCTGCCATCAGATACAGAATAAGGAGTGGGAGAAGGT
AAAGGGTAAGTTTTGCTCTTCTTCTCTTCTCTCTCTTATTTTTATTCCCCGAGGCATTTG
AATGATGTAAATGCATTTTTTCTCGTCCGTCCTGTGTGATGCTCTGTTGTGCTACTAAAA
AGAAGCATCTGTTGCTACTATTTATTGTATGCTATTTAATTATATAAAGAAGGATATATT
ATAATGCTGAGAATGAAAAATGCTTAATTCTCTTAGTATCTGCATTGTGTGAATAACTTG
TATTGCTGAATCAATATCAATTGAGAAATGAACGAATAGAACTGTCCTTGTTCTTCTTTG
TCATGTTTGTGTGGACTTTGTTGTGTTTCCTCCTTTTGTTGGGACAGAGTTGCACCATAA
TTTTAACCTGTGGTTTGCATGAGTTCATCTATATTTTAGGTTATGGTGATATATATGACC
TTTGGTGGATGGTAATTGATTGCTTCTCTTATCTTGACTGCTGATGTCAAGTTTTCTTGA
GAGGTTTCATTCTTGCTTCTCATTTGGCTGCTGTTTGCGAACCTTTTTATGGCATTGGCA
CTTTGAAAACTAGAAATGCAAGTAATATGAGTCGGGAAGTTAGGCTGCCTATATGTACAT
CATTCTTGATTAGGATGAAATACATGCTCAATGATTCAATCCCATATCAATTCTGAAAGA
TTTATACGAACTAGAAGCCTTAATGTATATGTAGTTTGGAGCTCGAGCTGAACTTTGGAC
ATTATTAACTGCAATAATTTGTTCAGATGATTTTTGCGAAACGTGAGGTGCAGTCACCAG
GTTGAATTTTTGTTACTGACCTAGAAACTATGTCAATGTAAATTATGCAAGAAACTTCTT
AAAAGTGCCATTTAGGTGTACTTTATACCAGAAGATCACTGAAGTGGTGCATCTCGTTTG
TTTAAATTAAAAAATCGAGACCATTTAAATAAGATTACATGGAAACCAAGATGTGGAATT
TAGAGGGCAATTGATTATAACATAGATTCTTTATGTTCGAATAACTTCTACCTTCTGTTA
GTTCTGGTATTAATTTGTGCATTTAATGCACCCTGTAGTTGGGTAATTTGAAGGATATAA
CTGCGTTATGTTGTGATATTTCCCACAATTAGATGCACGTTCGCATATTTTAATTGAAAA
GAGAAGATTCAGTTTCATACCTAAATTGGTAAAATATATAAAAGCCTTATCGTTCGTGGA
CAGCATCGCATAAATGGGAAACGGATGGCATATTTCTTATATGTTGATTAATTGCTCTTT
GCAATATTATATACGGTAACGGTACCGTCCTTTTTTCTCGCTGCATGAAATCTTTCTCTA
TTGGTTCTATTAGTAGTCAGGTCTTTTGCCATGTTGAAAATGGGGTTGATGAAGATGTGA
AAGGTAGTTACAGATGTTATTTTAGTTGAAGGAGGAATTTGGTTGACATGTATGCACTAT
GCACACTTGTTCTGTTTCTTTTTTTTCGGGTAAGATAAATGATTTATATGTTTCGATGAT
TGTTAGACTACTATTGTCTCTGTATTGACAATGTAAATGATCAATTGAGGCTTTCAAGCA
GATTTCTGACAACAAACTGTTGATATTTCTTTTCCTCTCCCATTTTATGTTCAATTTATC

TCCAGTTGATCTATACTTTTGGAGTTTTGTGTCATTCACAGTCGAGATGTTCTCTTAATA
GTTCTTAGATTCAGCAACTTATGTAATGCATTTCCCTATTCCCCTTGTCTTTTAAGGATA
TCAGCTTGAAAGATAAATCTTATCGAGCCATAGACCTTATGCTTGATTGAGGGTGTTTGT
GTTTTGGTAGCGTAATTCTTTATTATATATTTATTTTTCACATTATTAATGAAGGCTCTG
CTGGCATTGATATATAAATTATGACATACTAATACTTCTGTATTTCGTGGTTTTAAGAAT
TGATGATATCATATTGTGAAATGTTAAAAGTCTGGTACTAGAAGTTTCAGTTGAAATTGC
TGCTTTGTGAAGTTTTGCAACGACTTAAACCAAGGATGAGTAATTCTAAAACTGGCAAAT
TGTTTGTCTGCTTAATACCTTTATATAAGACTTGTGAAAAAAACTTTTGTACGTGATCTGA
TTATCTGGCTTTCAGTACTGTAAGTGGGTTACGGGATTGCAAACTGAACCAGCAAAGCAA
TAGCCTGTCTCTCTGATTTTTCTATTCCGTGTTCATATAAGTCTGTTTTGAATGTTTCAC
ATTTCTCTGTCTAACTACTTGAAGCATCTAGCCTTTTTTAAGTTGTTGAAAAGGTTTATT
CAATACCGTTTGTGATAGTATGATGTATGTATTGCATCGGGTGGGAAGCCACATTTTGGA
AGTTATTTTTGAAGCATTAATTGCAGCTCCCAATGGCATATTCTTTTTCCGGATTTTTCG
AATTCTAATTAGTGTGTTTACATGAAAATTTGCAATTTAAGGAATCAAAAACGGGAGCAA
AGGGAAACACATTGGTGCTTGGACTGCTGAATCCTGAAGCAAACAGAAGGTGCTCTCACA
AAGCTCGAGTTTTGATTCCTCTGAGACACCTGCCTGAACGAGAAACGCACAACCCTCTCT
TTTCCCGAAGATCAAAGAGTTCTTCTGCGTTTCGTCGGTTGCTAAACAGGTTCAGACATA
GCTGCAGGTCATCAAGAAAACTGGCGAAGGAGGTGGTGGGTCTTGATTTTCAACCTATGT
ATGTAGTCCTATTGAATAAGAGACCAAAGTATATGTAAATATTATAACATTTTTCTATAA
TATCTGATGATTGTTGAAGTTTGTCTTTTGTATAAGATTCTTTCTAGTTACCATATACAC
ATCATGCTTTGCCAGATTTAGGTAAACCAAGTAGAACCAAATTTAAGATAGCAAGTCAAA
ATCTTGACTTCAGATGGTGATCTGGCTTAACAGCTTTCTTTAATTAGATCTTCCGAATCA
ACCACCAAATTGAACTGTCTCCATCCCTCCAAAAAGATTAAAAATTTAAGCTCCCCCGGT
AGGAAGGATTAAAAATTTAATCACATGATTAGCTTCTCTGGTAGACTGACATCACTTTAT
ATTATCTTCATTGTTGTTTACTCTGCCACTTGATTTTCTTTTGTTTGTGTATTTAGGTGG
ACCATCTTGTAGGGTCACTGCATTAGTATTCTATGTTAGACGAAAGCATTGGTTGTGATT
GAGTCAACAAAATCAACCTAAACCGCATCATCACATTGTGGCTATTATGTGAGGAATCTG
GGTGAGTGTCATTTTCCTATGCTTTTTTTTTTTAGTGCATGAATGATGTTATTGGATAGC
AGATAGAATTGACATGGTGATAAGAAATGAGCGATAGCCAATTTCTCTTGTATGAAAATA
TTTGGGGATAAGTTATCTGGGGTGTCTGGAATCTGCTTCCTGTTTTCAAAAGTGAAGATA
AAATGACCCATTGCAATAATGGGAGTGTAGAATTGAAACTCTTACCCAATAAATTTGACT
CTTTTAAAAGGG
12>NISI10G0044
GGAAAATTAAAGGCCGATCTTCACGGTTTCAACGTTTTCGTAATTTTCCCCAAGTAAGAA
AAAAGAAAAAAAGACAAAAAATGAGATAGAATCATAGAAAGATGCGGGTCGGTAAGTCTC
AGGTATGGCAACCATGCAAAAAGAAGAGGTTTTAATCTACTGTGAAATCGATCTCCGGCG
TCACGAGAAATCGGAGGAGAAATAGCAGTCTCTGAGGAGAAGCGGAGATAAACTCAAAGA
CAAAATTATAGGAACGGAAGGGATCTAGGGATCGGAAAGAGGGCGAGAGGGAGAATTAGG
AGAATGACGTCTCGGACGGGCGGAGCGAGGACGCGTGTGGGAAAGTACGAGTTAGGGAGG
ACACTGGGAGAAGGGAGCTTCGCGAAGGTGAAGTTTGCCAGACACACTGAAACAGGGGAG
AATGTCGCCATCAAAATCCTTGACAAAGATAAAGTTCTTAGGCATAAGATGATCGGTCAG
GTAATTATTTTCCTTTTAAATCATGAATCACCACTTGAATTCGAATTCTAAGGTATTGAT
TCAGTTTTTGTGGTTTTTCATATCTCTTCGAATTATTTCTTACTAATTTTTAGGTCACGG
TGAACGAATTTTGATTAATAAATAAGTCAAATTGTATTGTTTCTTCGTATAAATTGATCA

GGTGTCGTGTTTATTTTGGTTACATGTTTTGATCGTGAACAGCCGAGTCAAAGTTATTAA
CTTATAGAACACTAATACAACGAATTGCTTTGTGATTTTTGGATACAATTCTTTTACACC
CGTTGGTTCTATTTTATTTTATCTGCAGTATTGCCCCTTTCTTGCAGATTAAACGCGAAA
TTTCTACCATGAAGCTCATTAGACATCCAAATGTCATCCGTATGTATGAGGTCAGAAGGC
CCCTTTTCATGCTTTTTCATGTTTAATGTTTCATATGTGCCTTTTCTTTTTCTTCTTTCC
TTGGATTTGTTTCTTGGTGTGGTGATCTCCTTGTATCCTAGCTCAATTGTTAGTACATCT
ATTCCCATTATATTTCTTTACTGACATTTAAAAGTCTTTTGGGTTTAGGTGATGGCAAGC
AAGACAAAAATATATATTGTTCTAGAATTTGTCACTGGTGGTGAACTTTTTGACAAAATT
GTAAGTTGGATTAATCTTCCAGTCATTATCATTTTTCCTCTCATGTTATCCGTGAAGTGG
CGAAAATGTTGTGAGTTGTTTGTTAGTCATTATATTTTTTTACACTATGTTTCTTGATGC
AGGCTAGTAGAGGCAGGTTGAAGGAAGATGAAGCTAGAAAATATTTTCAGCAGCTTATTA
ATGCTGTGGATTACTGCCATAGCAGAGGTGTTTATCATAGGGACTTAAAGGTGTGGGTTC
TCATAAATTGATTGATATTACTGAGATGCATAAACTTTAAACAGGCAAGTGTGATGAAGT
AATAACTTTTATATAAGATTTGGATTGAAGTTTGGTTTACTATTTTGTATCGCAGCCTGA
GAATTTACTATTGGATGCTAATGGAGTATTGAAGGTTTCAGATTTTGGGTTGAGTGCTTT
ACCTCAGCAAGTTAGAGTAAGTGATATACCTCATGTCTCAAGTATTTCTTTTGTTGTTAA
CTCTATATTTAAGTATGGTGGATCACCAAAGGACCATTCCTGTCTTGCCTGTGTCTCAGG
AGCCACTTCTTCATGATGATCTTTTGATCCATAGATTTTCTAATTATTATACAAAGACTT
TGGAACTTATCATCTGGTACTTTAATACAGGAAGATGGTTTACTTCACACAACATGTGGA
ACACCTAATTATGTTGCTCCTGAGGTATATTTGCTTTAGGTACAAAAGTATTATTTCCTT
ACTCTTCTGGGCCTGTAACTTATCTTCTTTTTTTTTTTTTTTTGTTTTTTGTGCAATACC
ATACCAGGTAATCAATAATAAAGGTTACGACGGCGCTAAGGCAGATTTGTGGTCATGTGG
TGTCATTCTTTATGTCTTAATGGCTGGTTATTTGCCTTTTGAAGATTCCAATCTCATGAA
TTTATATAAAAAGGTAATTTGTCCCTCCATTCTGTTTATCATACTTTTATTTTTGTTATT
AGAATTTTCTCAAATATCATTTATTTGGGAGCTGTTACCTTGGCCCAGTCAAACAAAGGC
ATTGGCAATGATTATCTACCAATATTATAATTCTTTAAGTGATAGCTGAATATTTGCATT
AATATTATCTCATAAGATTTTATTATAATTTATCCTCCTAAAACTTTCATGATGTGAATC
ACCTGTATGTATATTGTGTTGTGTGAAGTCATGGAGACTCCAACATCATCTGCCTCGTGA
CAAACATGTATTTTAAATTTCGTTAACCCAAAAACCAGTACTGGGCTTCCTGCTTTGAAT
CCAATGACATAATTGACATTATCTCTATAATGATATGTGAAGTTTGATTGACATCATTAT
TATTAGCTATTTTAGGATAAGTTAAATAACAGCTACATGTGTAAATGATAAGTGTATCTG
TTGGTGTACTTATTCTATTAATTGGTATGAAGAGTTGAATTTTGTTTTTTCTGGGTTAAT
CTAAATCTAGATTATTAATTTTCAATATTTTAATCAATCTGCCGATTCTAATGTTTCTTG
GAAAGAATGGCATCATTTGGTTCTTTATTGTGGTTGCAATTTAGTTATGTATGCATAATG
CTTGTAAATGGTGGAAATGACCTCATGAAGAATAACTTTGACAGGAAATTCCTTCTGGGA
TTAATGTGCCCTGTTATGGAAGCACTTGTGTAGTTTTCCCACCAAATCAGAAACAATGCA
ATGATAGCTAGCATTCCAATATTGAACATATTTCCCTTTTTCTTGTACAAAAACCAAAAT
TTCTCAGTTACTTGAATTTCTTATTATTTTATTTTTATCTGGACACAATTTTTTTGTTAA
GGTCCTGTGGCAATTGACATGGCCATGTCGTAGCAGCTGTACATTGGTTATGATAAAAGA
CTAATAGGTACACTAATGTGGGTTTTAATGGTTACAGATATTTAAGGCTGACTTCAAGTT
CCCTCCTTGGTTCTCATCAAGTGCAAAGAAACTAATCACAAGAATCCTGGACCCTAATCC
TTCAACTGTACTTCCTTTTCTCTCATACTTATGTTTTTAGTTCCCTTTTTTTCCAAGTGA
AAATTGTGTCGTAGAAAAGGATGGGTAACTTCAATTTGTAAGTATATTTTGACTTATTAT
GATGATGGTAATTATTTGCTGGTGGTTTGAAACAGCGGATAACAATTGCCGAGGTCATTG

AGAATGAGTGGTTTAAGAAGGGATATAAGCCCCCTGTTTTTGAACAACCTGATGTTAGTC
TTGTTGACGTGGATGCTATCTTTAATGAATCGGTGGTAAGAGTTGTGTTTCATATTTATC
GGGATATTGTGGTCATAATGTAACAGGTTTTGACTTGCTGCTTCATGTTGATTTCTTGAA
AAGGATTCTCAAAACCTTGTGGTGGAAAGGCGTGAAGAAGGGCCTAGAGCACCGGTGACT
ATGAATGCATTTGAGCTGATTTCCACATCTCAGGGTCTCAACCTGAGTACTCTTTTTGAA
AAACAAATGGTATGTTCAATATTGATGGCAGGTGTTCATGAATGAGGTAGTCTAGGTATA
ATATTCAATTTAATTCGAAGAAGCATTTGTCAAAATAGTCTTGAGCAGGGCCGGCCTTGA
GGGTTTTGGGGCCTAAGGTGAGAAGTGAAAATGGTGCAATCTAGATACAAAAATAGTAGA
TTTGAATTTTTATTTTATTTATTTATTTATTAAATAACAAGTTACATATATTTCAAAAAC
AAGTGTTTAAGTGCCATTAAAAAATATAAGTTTACATTTAAAAAAGAAAATTGACTAAAT
GTTGCAAACATTTAAAAAAAAAAAAAAAAATTAAAATAAAAAAAAATTGATAGGGAAAAGAGT
ATGTTGAAATGTTACCAAAGTAATTAAGCAGAGGTACCAAAGTGCAATTAACCTTTTAAA
AAACTGAAGTAAAAAGTCACAAAAATAAAAAAGAGAAAAAAAAAAAAATCCCAATAAAAGT
ATATTGCAATACAGATTTTAAATCTATATGTTTTATGTTCAACCTTCATGTATAATTCAT
ATTCTAAAATCTACACACTAAAAGTAATTTGAGTAAAGCATATTTATCAGAAAAGACAAG
AATGTAAAGAGAATATAGAATCAAAGTGCTAGTTTTTATAAAAGCGATTCCAAGGTGTAG
TTAGACAGAGGTGCCAAACTGAAATTTAACCTTTTAAAAAACTGAAAAAAAAAGGTTACAA
AAATAGAAAAGAAAAAAGAATCCCAATAGAAAAAATTGTATTAAAGACTTTAAATATTAG
CTTCATATTCAACCTTCACGTGTAATTCATATTTTAGAAATCTTCTACGTGCTAAAAATG
CAACTTGAGCAAAATAACACTATATGTTTCGGAAAGTAGGAACGAAATTGTCAAAAGAAG
AAAAAGAGAAATATATAAAGAATATATGGTATTAAAGTGCTAGATTTTATAAAGAAATAC
TACTACAGTGTAATTAGACAAAACAAGAGGTATCAAGGTGCAATTAACCCATTTTTTTTT
AAGTCTTTAAGTGTTTCATGTTTAACCTTCACATGTAATTCATATTCTAGAAATCTTCTA
CACGGGTCAAAAGTAACTTGAGTAAAGTAACATTATATATACTTGAACATAGCAATGGAA
CCCAAAAAAAAAAAAAAAAAAAAAAAAAAAAAAAAAAAAAAAAAAAGTGTTAAGAGAAAAATA
TAAAGAAGATGTGGTAGTGCTAGTTTTCTTTAAAGAAATAGTACCAAACTATTATTAGAC
AAAATAAGAAATACAAAGATGCAATTAACCCTTTTAAAAAACTGAAAAAGAAATTTTTGG
AGGGTGCTGAGACTTGATCCCAAGCTCCTCCTCAAGACCTTAGCTTCCCTGATCAGTCTA
TCTATTTAGCCCTTAAATTTTTTTTTTTTTTATCTCGCAAAATATATACATATCGTTTTA
TCTGTTTTTATCTTAAGCCAACTTTCCGATTTTTTCTCGGAAATGGTGCTCCCTTGCCAG
ATGGGGTCTAGTGCAGTCGCCTTGGGTCAAGGCCGGCCCTGGCCTTGAGTAAAACAAATA
GTGTTAGAGATCCTGGGATTTGCCATTATTTTGATGTTGTACAGCATGTTCTCCTGTTGC
ACTCCTGGCAGTATGCAGTTTGTAATCACATAGTTTGACAGGTTACAACTGGCCTCAGCT
GGGTGTGGTGTTGAGTTTGTTCTGGAACATGAAACTGAAATCACCTGACATATTCTTGTG
TTGTAGGGACTTGTCAAACGGGAGACAAGGTTTACTTCCAAATGTCCTGCTAATGAAGTA
ATATCAAAAATAGAGGAAGCTGCACAACCTTTGGGATTTGATGTGAAGAAAAGCAATAAC
AAGGTCTTCTTATATAACCTTTGTTTCTCATTTATCTTGATCCTCTATGCTTCAAGTGAC
AATTTAGCTTACTTTTAGCCATCAGTCAACAATAATTTAGGGCTCAAGAAAGGAAGTCAA
ATCACAGGAATGCTTCCATCTTGCATCCTAAATTTGAATTCTAGTTTATTTTATTCTCTC
TCTCTCTCTCTCCCCCCCCCCCCCCCCCCCCCCCCCCCGCTTCTACCTTACTCAAGTGTGT
AATATTAGTTGGAGTTCGATTAAAATTTGACACTGAACTTATTAAGGTTTCTCTTTGTTT
AATTTTATATATCTCAATAAACGCTATTGAATTTGTTTTTTGGAGTTGTCCCCTGTATAA
AGGCTGTCCATAATATGCATGAGGTCGTAAGTATTTGGGACTTGTATCATAAAACACAGG
ATTGACTTGGGATGAGAATATACTGTGTTAAGTACATAAAAGGTAAAATGTGCTTCATTT

CTGATGCAATATTGTTTGATTTATCCTTCTCAGATGAAACTTCAAGGGGAAAAAACTGGA
CGGAAAGGTCATTTATCTGTTGCAACTGAGGTAGGGCTCTCATTCTTATTCTTCAAGTCA
CCCCTTATAAAAATGTTGTTGCAGCTCAACAATGTCAGGTGATTTGTCCGAGTACTGAGA
TGATTCTGCTTTTGCAGATTTTTCAGGTGGCCCCTTCACTTTGCATGGTTGAACTGCGAA
AGTCTGGAGGAGATACTCTAGAATTTCACAAGGTATGAATATGGTTATGTCAAATGATCT
CCTGATTGTTTGGGTTGAACTTCAATCTATGCTTCTGTGGGTGAGAAATTGAGGGCTGAA
AATAGTTGAGCTACATTTACATCCTGTAGTGTGTTATTACACTGTTTGTCAGCTATTATG
TGGAAGCCTAAGTGGTTAGCCAGGGGTCAGAATAAAATGCATGTAGTTTCTACACATGGA
ATTGAACAGACTTTTAGTGGGTAGCATTGGGGTCTTATTGTTCAATGAAACAACTTGAAT
CTAGAGCTGCATCACTAAGTAATGTCCTGGAAACATCGATAGGGTTTACATCGGTGGACA
GGATAGTGATTGTCAAACTCTAGTCTTTCAAGTCTCATGTATCAGTTGAAGAACCTTTTG
CACACCTGCATAAAATAGTGTAAAAGTTACTCTTCAATTTATGCAAATCCAGTTCAATGT
TGCACTTAATGATCATGTTGTACACACGCATGATATGAATTAGTGCACAGAAGTCTGGCT
TTTTAAAATATAGGTCTTAATATAAGATGATTGAACTGTTTAAATTACTGTTGATGATGT
GGAACAGAAATATCTAGAGTGAACTACTAATTACCTATTAGGACAGTTTGATTTGGTATT
GTGCCAAGTTCCTCATTAGGGCTTAACTTGTACTCGAGAAACTTGAAGAACCCTTAGCTT
CGTGGTAGTCAATAAAGAGTGATACCACTTGCTTTACCTTTATATTTAACTCTGATGTAC
ACTCTGACAACTGACAAGACAGGTTTGAAATAATGTGTTGTCTCTCCAACTGCCAAATTA
AGTGCTGTGGTATAAAGTGGCAATTTGAGGGCGAGACATGTAGCATTGCTTAGATATAAT
AATGCTTAAATTATTAAATAAGCACTTTTATATTGATTTGTCAAACACAAAATCGAAGAA
AAATTATTTAATAATCACTTATTAAATATTTTAAGCAATACCAAATGGGGTCTAAATCGG
GCTTTTGCCTGAAAAGTTTGGTATTTAATGCCAATGTGAGCCAAAAAGTCCCTTATGTTG
CAACTCCAAGTTGTGTACCCTAATGTACCCTTGGAACAGAGTTTCATGAACTAGTATATT
ACTCAAGTTGCTTTAGGACCCCTAATGTTACTTATTGCTAGTTATGGATCATCTTTTTTC
AATACTGAAAATTCCTTGATTTTATCTTAAATATACAGTTCTACAAGAACCTCACGACAG
GGCTAAAAGATATTGTTTGGAAGCCAGCTGATGAAATAAAGGAGGAGTGACGGATGGTTT
GTATACTCTGTAAGTGCCTCTTCTTTTGGTCAATTACCATTCTACTGTGATAATTGCTTT
ACTGGGAGTCGGTCAATCCATGCAACATAAGCACATAGGATTATTTACCGTGTAAAAGGA
GAGAAAAGATGGTATATGTCTGCTAAATTGAAACTTATAGAAGGGAATCAACTTGTCAAC
AAACAGGGTTTGAACCAAGAAAAACAATTTTTGTAGATAGTGAAAATTTTTGGAAAGGAT
TTAGGCAATAAAGAACTAATGTAAACAAAATCAAGGAGTTGTGTAAATCTGAGCACTCTT
CTTTTTCGGTATCCAAGCTATTTTCAGAGTTTGAAAGGCATTATTAAATCTTGACTTGAT
ATCATTTTTCTGATGGTGTCATTCTACAGATGCTGTAGCTGCTTTTTTTCACGACTCTAG
ATCATGGGAGGGGCAAGATCCTTTTGTCAAAACTCAAAAAATGGAGTTGTTCTACAGACA
GATTTATCTTCTGGGTTTCATCATTGTATTTAGTTGCCTTGCTGTGTATTCCTTCCGCCC
TGTGTGTTTCCTTCAACTTTGACTTCTGTTACAA
13>NISIScaf80G0007
CTCTCTCTCTCTCTCTCTCTGTACTCCTCTGACCTCTCCCCTCTCCCTCTCTATATTTTC
CCTTTCCCCTCCCTTTCCTTCTTCAATTCTTCTTCTTTTTTTAATTCAATAGACTCTCCA
TAGCTTATCTGGCCTTTTGCCTTATCTGATAGCCACATATCCATCTATATATACTTGGAT
TCATTTCATTCACAAAGAGGAGAAAATCAAATATCAAAGGATTCATTCCCCGCCTCACAG
ACAGAGACAGGATTAACATTGGATGTTCAACAAAAAGTTAGGGGTGGGAGCAACTGCAAC
TGCAAATACAACTACAACTACAACTGAGGAGGATCCCAACAAGCAAGGAATGCGTCTTGG
AAAGTACCAACTTGGAAGGACTCTCGGGGAAGGCAATTTCGGTAAAGTTAAATACGCTAA

GAAATTGGATTCTGGCCTACCCTTTGCTGTCAAAATTCTTGAGAAGACCAAGATCTTCGA
CCTCAAAATCACTGATCAGGTTCCCTTCCCTTCCCTTCTTATTTCCTGCCTCAGTTTTAA
TCTTTCCTGGAAATTCAAAACCCAATACGATCATGTTGTTTCGTTTTTATTATGCGAGTT
AATTAATTAGTTGTCTACTTGTCTTCCCTGTTATAATTCACTATCTGTATCTGTATGAGG
TGGATTGATTTCTTAGTCTTACCAAAATATGTTTGAATTGGACTAAATGACAGATAAAGA
GGGAAATTGGGACTTTGAAGCTCCTTAAGCATCCGAATGTCGTCCGATTACATGAGGTAA
CCCTCCCCTCTTCTACCTCTCAATCTAACTTCCCAATCCCATCCCATCATCAGATGCTAT
GCTGTTCAAATTATGATTGGTTTAATCATCAACATCATGATACTTTTCTAATCTTCTTCT
TCTTCTTCTTCTTCTTTTTATTATTATTATTATCATTATTATTATAAAATAAAATAAACA
TATAAAATGAAATCCTGGCGGTTATGATAATGGATCGCATCACAGATATCGAATTTACAA
TAATAGATTTTAAAATATCTCGCTACCCCTCCTCTGCTTTTACAACAGGGCCTAAACAAA
TGCAACAACTGTACACCTTCTTCTTCAGAAATAAAATTAAAAAAAAAAAAAAAAGAAAAAA
AGAAAAAAAAAAAAAAAAGAAGAAAACAAAACTTGATTTGAAATTGTGTTGGAATGGTATT
GTTTGTATTCCTAGTATAGTAAAAAGTTATTTAATTCAGATTAAATCTGGGTTGGCATTG
GCAGATAATATTTGTATATATGAATGTTAAAAATTATATATTATTGTGATTGTGTCGGGC
TGACTGATATTATAAGAATTCTGATTGTTGAAGAGTGAGTGAATATAAACATCAGATCTG
GAGTCCAGACTGTCTGTCTTGTTACAATTGGGTTCACATGCACAGGGTCATCGGCATGGG
ATTGTTTTGATTGCTTTGAACCCTGACCCTGACCCACACCCTGACCAAACAACCAAATAC
GATACAAATACGTAACACGTTCCTTGACTCTCCTTCCTTCCCTTCCCTTCCCTTCCCTTC
CCTTGTATATAGATAATAATACATACGTGGGCCTTTGCCTCTTTTTTTGGGGTGGGTGGG
GGCCAGTCATTTGATTATAGGGTGGGGGAGGGACCCTCTGCTGTCATAAAATTCTGTGGT
TAATTTTGGCCGGATAACAACATCTGAACACTATTCCTATAGGCTCTTAATTCATTTGGG
CCCACCCCAGGCCACCCACAATTATTAGATGTGGCATTGGCAAATTATAACATGGAGTAT
GTAGTTCTGCTTCTGCATCCCCTCCCCAATTCAATTTTGTGTTTTGTCAATTGCCCATGT
ACGCATCATCTATTGTTGTCTTTTTCTGGGGTGGTTCCCATCTGGGGCTGTCATTGGGGG
ATGCGATGGGATGGGAGGGAATCTCACTTCCCAGATATTATCATCACATGGAAAACCCCC
CTTTTTTTGGGCTTTCCCTGCTGCCCCTGTCCAGCACACCACTTCTCTCCATCCATTTAT
TTGGATTCATTTCCACCACCCCGATACGAGTCTACGACCCTACTTAATTATACCTAAATC
CGATTAACTACTTCTTCTGATATCATTTATTACTTACTATCATGATTAATTATACTTTTA
CATTAATAAACGTGTAGGTCTTGGCAAGCAAAACAAGGATTTATATGGTGCTAGAATACG
TAACTGGTGGAGAGTTGTTTGACAGAATTGTAAGTACCCCCAAAAGAACGCACCATATAC
CATATGATTCTTCCTCAATTAATTAATAAAAAAAATATGCAAAAGCATTACCAAGTATAT
ATTATAATACAGGCATCTAAAGGTAGACTGCCTGAAGCTGAAGGAAGGAAGCTCTTCCAG
CAGTTAGTTGACGGTTTGAGTTATTGTCACAACAAAGGTGTTTTCCACCGGGATCTCAAG
GTTACTACCTCTTTTCTACATATTTAATCCAGTTATAATATACTTGGTTCAACAATTAGT
TAACGCTTTGGGTTATTGCAGTTGGAAAACATTCTTATTGATTCTAGAGGTAACATAAAG
ATATCTGATTTTGGCCTCAGTGCTCTACCCCAGCATTTTAGGGTAAGCTTCTCTCTTCCA
TGTTCATATTCATATATATATTAATCCATCAAAACTTTATATTCAATTATTGTATTTTCT
AGGATGACGGTTTACTGCATACAACTTGTGGAAGTCCAAACTATGTTGCACCTGAGGTCC
TTTCCAATAGAGGGTATAACGGTGCCACCTCAGATATCTGGTCAAGCGGTGTTATCCTAT
ACGTAATTCTCACTGGACACCTCCCTTTCGATGATAGGAATCTTGCTGTTCTCTATCAAA
AGGTATTGTAAAGTTGCACCTTATCTTCTTTTTCCCTTTTTGCATGTTGAAAATTCACAT
TCTTAAGTTAAGAACTTTATCATCTTTGACATCAGATTTTCAAGGGAGATGCTAAGATAC
CAAAATGGTTATCTCCTGGCGCCCAAAACATATTAAGAAGGATTTTGGATCCAAATCCTG

TTACTCGTTTGACCATGTCTCAAATCAAATCTGATGAATGGTTCAAGCAGGGGTACACTC
CTGCAGATCCCACTGAAGAAGAAGAAGAAGATATAATCATCGATGATGAAGCCTTCTCAA
TTCATGAATTGGTATGAGCTTAGAAGTCTTCTGATACTTACATGGTCATTTGATCGTAAT
AACTAAGTATGTTTTTTTTTTTTTTTTTGGCAGCATTCTGATGGGGAAAAGACACCAGAT
TCACCTGGCACCATCAATGCTTTCCAGTTGATTGGAATGTCATCATGCCTTGACCTCTCT
GGTTTCTTTGAGAAAGAGGTGCTTAGCAAACATCTTCTACATGTCTTTTGTAGTTGATTG
GCCTTAACAAAGTAGTTAGCAGACCTCTAAAATCTTCATCTCTTTTTTTAATTTTTAAAT
ATAGGATGTCTCTGAGAGGAAGATCAGATTCACATCCAATCTCTCCATGAAAGATCTGTT
GGCGAGGATTGATAGTATTGTGACAGAGATGGGATTTCGAGTCCAAAAGAAAAACGGAAG
GGTTAGTACTATTTTACCATAAATTTACTGGCCAGTACTTTTGTTTTGTGGTATGCATTC
ATATAAATTGAGTAAATATCATATATTGTAAATCAAATGTGGTGTTCTGGTACTGGTAGG
TGATTTCTGACTTTAATTTGCATGCTTTCTATGTACATGTAGTTGAAAGCCACGCAAGAT
CACAAGGGACAGAGTAGCTTAGGAAGTCTTTCAGTGGCAGCAGAGGTATTTTTGGGAATC
ATTCAGATAATTCAACGTGCATGTTGTTTAATTGATCTTTTGTATTTCACTCAATCAGTG
CTTGTGTTGTGCATTAACATAATTTTTGACTCGTATGGTTCAGGTATTTGAGATTAGCCC
ATCTCTATATGTGGTTGAATTGAGAAAATCATACGGAGACTCTGCTGTATAGACAGGTAC
CATTTTTTTTTTTTTTTTTTTTCTGTAGCTGTTAGCTCTATGAAGATCATCCCCCACCATA
TTTGCTGATAAAATATTTGGGTTGGTGCAGTTGTGCAAACGAATATCAAGTGATTTAGGC
GTCCCCTCAAGCCAAGGGTTGGTTGCTACACAAGTGTGAGAAAACGCTGGTTGTAAATTA
CAACCATGTCCGAAGCATCAAGCCTTGGGGCGGAGGAGACAGGAGTGGATATGGATTCCG
TTTGAGAGATGTAGTTAGGTCTTTGATTCTTTAAGGTGTCTCAAATTGGAGTGCCGGAAT
TGTAGATATACTAGTAACAGTGCGTGTGTATATATATATATATATATTTAGTTAGATAAT
ATAAATATCCAGGTTGTTAAGCAGTCAGGATTATCATATCAATTGTACGTATGCATTTTC
TATTTGAAATTTTGTCATTCTTATTACATCGATCTTGTTTAGCCTATGTTTCACAGCCAT
AGGGGGTTTGCTAAATTGATTTCTCATCTGATATACGACATTACTTTTTTTTTTTTTTTT
TTAATATTATATCCAATTAGTTTTTAATTAAACACATGATATATAACATAATATCTTAAA
ACCGAAAACATTCTTACATAAGACTTCAGTGAACATACGCTTCTCACAAATGCAGTCAAT
TTTTGGATAATAATTTTTACATAAGAACCCAAATCCCACATTCCATACTTGAAGAGAAAT
ATTAACAGCACTCTCTAATTTCTAATGCACTCACTCATTATATGACCACTTTATACTACT
AATAACACTTTATACTCTTAAATTAAAACCTTATTTCATTTATCAACAAACCACTCCTTT
TCAGAAAAAGTACTCTATGAATTTGTTTGTCCATGAAGAACAATCTTTTGTTATTTATCA
CTCCATGTTAAATTACAAATTTGCAATACTACGCCAATAACCTCTTAAGATGTTTAGTAT
TTGTTATAAAAAAAAATTCATGTACAAAAATGAGAGCTACATGCCAAAAAACAAATAAAG
CTTAAGAGGTATCGTCATTTGTTTATGGGGGAGAGAGAGTGTTGCTAGCATTCATCTTAC
TTTAAACTTAGAAGACTTAAAAGAGTGTAAAATGTTGTATAAGAATGAACCAGTCCCGAC
CTGGACTCCACTAATTATGATAAACCCTTTAACTAGTTGACATAAGTCTTTGTTCCCACA
CTAATCTCACACAACACAAAAGGCAATTCTTTACCTACAGGCTACAGCTACAGCTTTCTC
TCACAATCCAATCCCATGCATACAAAAGAACAAGCTTTTCGTCTATATTACAATAGTAGA
TGAAGGTCGCACTAAAGTTTCAAGATATACAGTGGTAAGGTATAACACATTAACATATTC
ACAGTAAGTAATTAATTTTCTATTCATGTACTACACAGTCGACTTTATCAAATGCTTGTC
TGAGTAGCAAGCCGGTTGCCAAGTTCCTTGCTATACACTTGAATTAAACCAGTGTTGTCT
GCCATCTG
14>NISIScaf97G0002
CCCTCTCTCTCTCTCTCTCTGTACTCCTCTGACCTCTCCCCTCTCCCTCTCTATATTTTC

CCTTTCCCCTCCCTTTCCTTCTTCAATTCTTCTTCTTTTTTTAATTCAATAGACTCTCCA
TAGCTTATCTGGCCTTTTGCCTTATCTGATAGCCACATATCCATCTATATATACTTGGAT
TCATTTCATTCACAAAGAGGAGAAAATCAAATATCAAAGGATTCATTCCCCGCCTCACAG
ACAGAGACAGGATTAACATTGGATGTTCAACAAAAAGTTAGGGGTGGGAGCAACTGCAAC
TGCAAATACAACTACAACTACAACTGAGGAGGATCCCAACAAGCAAGGAATGCGTCTTGG
AAAGTACCAACTTGGAAGGACTCTCGGGGAAGGCAATTTCGGTAAAGTTAAATACGCTAA
GAAATTGGATTCTGGCCTACCCTTTGCTGTCAAAATTCTTGAGAAGACCAAGATCTTCGA
CCTCAAAATCACTGATCAGGTTCCCTTCCCTTCCCTTCTTATTTCCTGCCTCAGTTTTAA
TCTTTCCTGGAAATTCAAAACCCAATACGATCATGTTGTTTCGTTTTTATTATGCGAGTT
AATTAATTAGTTGTCTACTTGTCTTCCCTGTTATAATTCACTATCTGTATCTGTATGAGG
TGGATTGATTTCTTAGTCTTACCAAAATATGTTTGAATTGGACTAAATGACAGATAAAGA
GGGAAATTGGGACTTTGAAGCTCCTTAAGCATCCGAATGTCGTCCGATTACATGAGGTAA
CCCTCCCCTCTTCTACCTCTCAATCTAACTTCCCAATCCCATCCCATCATCAGATGCTAT
GCTGTTCAAATTATGATTGGTTTAATCATCAACATCATGATACTTTTCTAATCTTCTTCT
TCTTCTTCTTCTTCTTCTTTTTATTATTATTATTATCATTATTATTATAAAATAAAATAA
ACATATAAAATGAAATCCTGGCGGTTATGATAATGGATCGCATCACAGATATCGAATTTA
CAATAATAGATTTTAAAATATCTCGCTACCCCTCCTCTGCTTTTACAACAGGGCCTAAAC
AAATGCAACAACTGTACACCTTCTTCTTCAGAAATAAAATTAAAAAAAAAAAAAAGAAAAA
AAGAAAAAAAACAAAACTTGATTTGAAATTGTGTCGGAATGGTATTGTTTGTATTCCTAGT
ATAGTAAAAAGTTATTTAATTCAGATTAAATCTGGGTTGGCATTGGCAGATAATATTTGT
ATATATGAATGTTAAAAATTATATATTATTGTGATTGTGTCGGGCTGACTGATATTATAA
GAATTCCGATTGTTGAAGAGTGAGTGAATATAAACATCAGATCTGGAGTCCAGACTGTCT
GTCTTGTTACAATTGGGTTCACATGCACAGGGTCATCGGCATGGGATTGTTTTGATTGCT
TTGAACCCTGACCCTGACCCACACCCTGACCAAACAACCAAATACGATACAAATACGTAA
CACGTTCCTTGACTCTCCTTCCTTCCCTTCCCTTCCCTTCCCTTCCCTTCCCTTGTATAT
AGATAATAATACATACGTGGGCCTTTGCCTCTTTTTTTGGGGTGGGTGGGGGCCAGTCAT
TTGATTATAGGGTGGGGGAGGGACCCTCTGCTGTCATAAAATTCTGTGGTTAATTTTGGC
CGGATAACAACATCTGAACACTATTCCTATAGGCTCTTAATTCATTTGGGCCCACCCCAG
GCCACCCACAATTATTAGATGTGGCATTGGCAAATTATAACATGGAGTATGTAGTTCTGC
TTCTGCATCCCCTCCCCAATTCAATTTTGTGTTTTGTCAATTGCCCATGTACGCATCATC
TATTGTTGTCTTTTTCTGGGGTGGTTCCCATCTGGGGCTGTCATTGGGGGATGCGATGGG
ATGGGAGGGAATCTCACTTCCCAGATATTATCATCACATGGAAAACCCCCCTTTTTTTGG
GCTTTCCCTGCTGCCCCTGTCCAGCACACCACTTCTCTCCATCCATTTATTTGGATTCAT
TTCCACCACCCCGATACGAGTCTACGACCCTACTTAATTATACCTAAATCCGATTAACTA
CTTCTTCTGATATCATTTATTACTTACTATCATGATTAATTATACTTTTACATTAATAAA
CGTGTAGGTCTTGGCAAGCAAAACAAGGATTTATATGGTGCTAGAATACGTAACTGGTGG
AGAGTTGTTTGACAGAATTGTAAGTACCCCCAAAAGAACGCACCATATACCATATGATTC
TTCCTCAATTAATTAATAAAAAAAATATGCAAAAGCATTACCAAGTATATATTATAATAC
AGGCATCTAAAGGTAGACTGCCTGAAGCTGAAGGAAGGAAGCTCTTCCAGCAGTTAGTTG
ACGGTTTGAGTTATTGTCACAACAAAGGTGTTTTCCACCGGGATCTCAAGGTTACTACCT
CTTTTCTACATATTTAATCCAGTTATAATATACTTGGTTCAACAATTAGTTAACGCTTTG
GGTTATTGCAGTTGGAAAACATTCTTATTGATTCTAGAGGTAACATAAAGATATCTGATT
TTGGCCTCAGTGCTCTACCCCAGCATTTTAGGGTAAGCTTCTCTCTTCCATGTTCATATT
CATATATATATTAATCCATCAAAACTTTATATTCAATTATTGTATTTTCTAGGATGACGG

TTTACTGCATACAACTTGTGGAAGTCCAAACTATGTTGCACCTGAGGTCCTTTCCAATAG
AGGGTATAACGGTGCCACCTCAGATATCTGGTCAAGCGGTGTTATCCTATACGTAATTCT
CACTGGACACCTCCCTTTCGATGATAGGAATCTTGCTGTTCTCTATCAAAAGGTATTGTA
AAGTTGCACCTTATCTTCTTTTTCCCTTTTTGCATGTTGAAAATTCACATTCTTAAGTTA
AGAACTTTATCATCTTTGACATCAGATTTTCAAGGGAGATGCTAAGATACCAAAATGGTT
ATCTCCTGGCGCCCAAAACATATTAAGAAGGATTTTGGATCCAAATCCTGTTACTCGTTT
GACCATGTCTCAAATCAAATCTGATGAATGGTTCAAGCAGGGGTACACTCCTGCAGATCC
CACTGAAGAAGAAGAAGAAGATATAATCATCGATGATGAAGCCTTCTCAATTCATGAATT
GGTATGAGCTTAGAAGTCTTCTGATACTTACATGGTCATTTGATCGTAATAACTAAGTAT
GTTTTTTTTTTTTTTTTGGCAGCATTCTGATGGGGAAAAGACACCAGATTCACCTGGCAC
CATCAATGCTTTCCAGTTGATTGGAATGTCATCATGCCTTGACCTCTCTGGTTTCTTTGA
GAAAGAGGTGCTTAGCAAACATCTTCTACATGTCTTTTGTAGTTGATTGGCCTTAACAAA
GTAGTTAGCAGACCTCTAAAATCTTCATCTCTTTTTTTAATTTTTAAATATAGGATGTCT
CTGAGAGGAAGATCAGATTCACATCCAATCTCTCCATGAAAGATCTGTTGGCGAGGATTG
ATAGTATTGTGACAGAGATGGGATTTCGAGTCCAAAAGAAAAACGGAAGGGTTAGTACTA
TTTTACCATAAATTTACTGGCCAGTACTTTTGTTTTGTGGTATGCATTCATATAAATTGA
GTAAATATCATATATTGTAAATCAAATGTGGTGTTCTGGTACTGGTAGGTGATTTCTGAC
TTTAATTTGCATGCTTTCTATGTACATGTAGTTGAAAGCCACGCAAGATCACAAGGGACA
GAGTAGCTTAGGAAGTCTTTCAGTGGCAGCAGAGGTATTTTTGGGAATCATTCAGATAAT
TCAACGTGCATGTTGTTTAATTGATCTTTTGTATTTCACTCAATCAGTGCTTGTGTTGTG
CATTAACATAATTTTTGACTCGTATGGTTCAGGTATTTGAGATTAGCCCATCTCTATATG
TGGTTGAATTGAGAAAATCATACGGAGACTCTGCTGTATATAGACAGGTACCATTTTTTT
ATTTATTTTTTTTTCTGTAGCTGTTAGCTCTATGAAGATCATCCCCCACCATATTTGCTG
ATAAAATATTTGGGTTGGTGCAGTTGTGCAAACGAATATCAAGTGATTTAGGCGTCCCCT
CAAGCCAAGGGTTGGTTGCTACACAAGTGTGAGAAAACGCTGGTTGTAAATTACAACCAT
GTCCGAAGCATCAAGCCTTGGGGCGGAGGAGACAGGAGTGGATATGGATTCCGTTTGAGA
GATGTAGTTAGGTCTTTGATTCTTTAAGGTGTCTCAAATTGGAGTGCCGGAATTGTAGAT
ATACTAGTAACAGTGCGTGTATATATATATATATATATATTTAGTTAGATAATATAAATA
TCCAGGTTGTTAAGCAGTCAGGATTATCATATCAATTGTACGTATGCATTTTCTATTTGA
AATTTTGTCATTCTTATTACATCGATCTTGTTTAGCCTATGTTTCACAGCCATAGGGGGT
TTGCTAAATTGATTTCTCATCTGATATACGACATTACTTTTTTTTTTTTTTTTTTTTTAA
TATTATATCCAATTAGTTTTTAATTAAACACATGATATATAACATAATATCTTAAAACCG
AAAACATTCTTACATAAGACTTCAGTGAACATACGCTTCTCACAAATGCAGTCAATTTTT
GGATAATAATTTTTACATAAGAACCCAAATCCCACATTCCATACTTGAAGAGAAATATTA
ACAGCACTCTCTAATTTCTAATGCACTCACTCATTATATGACCACTTTATACTACTAATA
ACACTTTATACTCTTAAATTAAAACCTTATTTCATTTATCAACAAACCACTCCTTTTCAG
AAAAAGTACTCTATGAATTTGTTTGTCCATGAAGAACAATCTTTTGTTATTTATCACTCC
ATGTTAAATTACAAATTTGCAATACTACACCAATAACCTCTTAAGATGTTTAGTATTTGT
TATAAAAAAAAAATAAAAAAAAAATTCATGTACAAAAATGAGAGCTACATGCCAAAAAAACA
AATAAAGCTTAAGAGGTATCGTCATTTGTTTATGGGGGAGAGAGAGTGTTGCTAGCATTC
ATCTTACTTTAAACTTAGAAGACTTAAAAGAGTGTAAAATGTTGTATAAGAATGAACCAG
TCCCGACCTGGACTCCACTAATTATGATAAACCCTTTAACTAGTTGACATAAGTCTTTGT
TCCCACACTAATCTCACACAACACAAAAGGCAATTCTTTACCTACAGGCTACAGCTACAG
CTTTCTCTCACAATCCAATCCCATGCATACAAAAGAACAAGCTTTTCGTCTATATTACAA

TAGTAGATGAAGGTCGCACTAAAGTTTCAAGATATACAGTGGTAAGGTATAACACATTAA
CATATTCACAGTAAGTAATTAATTTTCTATTCATGTACTACACAGTCGACTTTATCAAAT
GCTTGTCTGAGTAGCAAGCCGGTTGCCAAGTTCCTTGCTATACACTTGAATTAAACCAGT
GTTGTCTGCCATCTG

1>NISI03G1060 [mRNA]
ATGGAAACCATGGGGAGCATATTGATGGAACGTTATGAATTAGGAAGGCT
ACTAGGTCAAGGGACTTTTGCCAAGGTTCATTATGCAAGGAATCTTAAGA
CTGGGATGAGCGTTGCCATTAAGATAATTGACAAAGAGAGGGTGATCAGA
TATGGGTTGATGAATCAGACTAAGCGAGAAATTTCTGTTATGAGACAGAT
TAAACATCCAAATGTAGTCGAGCTGTATGAGGTCATGGCAACCAAAACCA
AGATTTACTTTGTGATAGAATATGTTAAGGGTGGCGAGCTTTTCAACAGG
TTGGCCAACGGGAAGCTCAACGAGGATGCTGCAAGGAAATATTTTCAACA
GCTAATAAGTGCAGTTGATTACTGCCACAGTAGAGGTGTGTATCACCGGG
ATATAAAGCCAGAAAACCTTCTATTGGATGAGAATGAAAATCTAAAGGTT
TCAGATTTTGGATTGAGTGCACTTGTGGATTCCAAGCGTAAAGATGGGTT
GCTCCATACAACGTGTGGGACCCCTGCTTATGTTGCTCCAGAGGTAATCA
ACAGAAAAGGCTATGATGGATCCAAGGCTGATATATGGTCATGTGGAGTG
GTCTTGTATGTTCTATTGGCTGGACATCTCCCATTCCATGATTCAAATCT
GATGGAGATGTATAGGAAGATTGGTAGGGCGGATTTCAAATACCCTAACT
GGTTTGCCGCAGATGTGCGCAGGTTAATCACAAAGATCTTGGATCCAAAC
CTGGTGACGAGGATATCTATGGACAAGATAATGGAAAGTTCTTGGTTCAA
AAAGGGGTTAGAAAAACCCATAACTATTGATTTAGAAGGGAAACCAGAAA
TCCCTGCGGATACTGACGCAACTTTCACTTTAAATGAAAGCAGCGGTGGT
CGTACTGTGCCCAAGGCGGAGAATGAGTTGACAAAGCCTTCCAACTTGAA
TGCTTTTGATATAATCTCTTTCTCCACTGGCTTCGATTTGTCTGGTTTGT
TTGAGGATAGGGAAGAAAGGAAGGAAATGAGATTCACATCCAACAAGCCA
GCTGCAACCATCATATCAAAGCTTCAGGATATTGCCAAGCGTCTGAAACT
CAAAGTAAAGAAGAAAGATGGAGGGTTGCTAAAAATGGAGGGCCCAAAGG
AAGGTTGGAAAGGAGTGCTGGGTATCGATGCAGAAATATTTGAGATCACT
CCATTTTTGCATATGGTGGAAATGAAAAAGAGCAGCGGAGACACACTCGA
GTACCAGAAGATGATGAAACAAGAGATAAGGCCAGCTCTTAAGGACATTG
TGTGGACATGGCAAGGGGAGCAGCTACAGCAGCAGTGCTCGGAACATATA
GCACTGCCATCACCAGTACAACAGGAAACAACTGCCTAG
2>NISI03G1063 [mRNA]
ATGGAGGAGAGAACGGTTGATGGCGGCGAGGTATTGGGAGGAGGGAACCT
GTTCGAGAAGTACGAATTGGGCAGATTAGTCGGTCGGGGAGCGTTCGCGA
AGGTTTATCACGGCCGAGATATTCGTACGGGACAAAGCGTGGCGATTAAG
GCCGTTGGCAAAAACAAAGTCGTTAAAGAAGGATTTATGGCGCACATTAA
GAGGGAAATCTCTATTATGCGCCGGTTGCGCCATCCTCACGTCGTCAAAC
TGCTAGAGGTCATGGCTACCAAAACCAAGGTTTATTTTGTGATGGAGTTC
GCCAAAGGTGGAGAACTCTTCACCAAGGTTTCCAAGGGAAGGTTTAGCGA
AGATCTCAGCCGTAAATACTTCCAGCAGCTAATCTCAACCGTTGGTTATT
GTCATTCTAGAGGAGTTTTTCACAGGGATTTGAAACCGGAGAATTTATTG

CTAGATGAAAATTGGGACCTGAAGATAACCGATTTCGGACTCAGTGCGCT
AAAGGATCAGACCCGATCCGATGGAATGCTTCACACTTTGTGCGGCACTC
CTGCTTACGTGGCACCCGAGATTTTGGCGAAAAAAGGTTACGACGGCGCT
AAGGTGGACATTTGGTCATGCGGCGTCGTTTTGTTCGTCCTCAACTCCGG
TTATCTACCGTTCAATGATCATAATATTATGGTCATGTACCGGAAGATCT
ATAAAGGTGAATTTCGGTGCCCCAAGTGGACGTCTCCGGATCTTAAACAC
CTCTTATCACGGCTACTCGACCCGAACCCAGATACAAGGATCACTATTGA
CGAGATCTTAAAGGATCCATGGTTCAGAAAAGGTTACAAGGAGGTAAATT
TCCATTCCGAAGATTCTGATTTGAAAGACTTGGGAGTTAACGACAACCGC
AAATGTTTAAATGCTTTTGATATAATCACTTTCTCTCCGGGTTTTAGTTT
AACCGGCTTGTTCAACGACTACACCGATGCCGAGAGATTCTTATCGGCTG
AAAAACCAGATAAAATTATAGAGAAAGTCGAGGAGATGGCGAAGAAGGAG
AATTTGACAGTGATGACGAAGAAAGGTTATGGGATAAAGTTGGAAGGACA
TGACGGTAATTTCTCCTTGGTCACAGCTGTTCACCGGTTAACGGATAAAC
TGGTTATTGTGGATATTAAGAAGAGAGAGAGAGAGAAGTTGGATCTGGTCAA
GAATTCTGGAAAAATAACTTGAGACCTCGGCTTCGTGGTTTGATTTATCC
ACCGGAAACGCCGGTTGCCGACAACTCATAG
3>NISI03G1369 [mRNA]
ATGCCGGAGATTGAACATGTCCCCGCGGATTACGACCGCAATTGCAACGC
TGCCGACGGTGCCTTGTTTGGAAAGTATGAGCTCGGCAAGCTCCTCGGCT
GCGGAGCCTTCGCTAAGGTGTACCATGCGCGTGACGTCCGTACGAACCAG
AGCGTGGCGATTAAGATCATTAGCAAGAAGAAGATCAACGTTAATCTGAT
GTCGAACATCAAGCGTGAGATCTCGATCATGAGGCGGTTGAACCATCGCC
ATATCGTGAAGCTCCACGAGGTTCTGGCGTCGAAAACGAAGATTTATTTC
GTCGTGGAGTTCGCCAAGGGCGGCGAGTTGTTCGCCAAGGTGGCGAAAGG
AAGGTTCAGCGAGGATCTCAGCAGGAAGTACTTCCAGCAGTTGATATCCG
CCGTTGGTTATTGCCATTCGCGCGGCGTCTATCACCGTGATCTGAAGCCG
GAGAATCTCCTGATCGACGAGAACGGGAATTTGAAAGTTTCAGATTTCGG
ACTCAGCGCTCTGACGGATCAGATCCGAACCGACGGGTTGTTGCACACGC
TGTGTGGGACCCCTGCTTACGTGGCACCAGAGATATTGTCGAAGAAAGGA
TACGACGGAGCCAAGGTGGATATCTGGTCATGCGGCGTCATTCTGTTTGT
TTTAACGGCCGGTTACCTGCCGTTTAACGACCCGAATCTCATGGCCATGT
ACAAGAAGATATACAAAGGCGAATTCCGGTGTCCGAAATGGATGTCCAAC
GATCTTAAACGGCTGTTAAACCGTCTCCTTCATATCAATCCTAATACAAG
GATTACCGTCGATCAGATTCTCGGAGATCCATGGTTCAGAAGGGGCGGGG
TCAAGGAAATCAAATTCCACGACGACGAAAACGCCGCCGTTCCGGATAAA
ACCGGTAAGGAGGGGTTCGGTGCGAGGAATTTGAACGCGTTTGATATAAT
CTCATTTTCGTCCGGTTTGGACCTGTCTGGTTTGTTCGATACGTCGTGCA
ACTCGTTCGAGAATAATACTGGCGAACGTTTCATCTCGCGAGAGTCGCCT
GATAATTTGTTGGAGACGGTGACGGAGTTCGCCAAGGTTGAGAAATTAAG
GTTGAAGACGAGGAAAGAATGGGGGGTGGAGTTGGAAGAACAAAACGGTA
ATTTCATCATCGGGGTGGACGTTTACCGGTTAACGGAGGAACTAGTGGTC
GTGGAGGCCAACAGAAGAGCGGGTGACGCCGCATCTTACACTGAGGTGTG
GAAGAATAAGCTGAGACCGCAACTTCTTGTGCGTCAACAGGAAGCTTCGG

TTTCTGGTAATCATTAA
4>NISI05G0308 [mRNA]
ATGGCGGCGGCGGCGGTAATTGCGGCAGTAGAGAAAAGCACTTCCAGGGA
TAGAAGCACTTTACTGCATGGCAAATACGAGCTTGGGCGGCTACTCGGCC
ATGGCACCTTCGCGAAGGTGTATCACGCGCGTCACTTGCAGACAGGAAGG
AGCGTGGCAATGAAAGTTGTGGGGAAGGAGAAGGTGATTAAAGTCGGGAT
GATGGAGCAGATCAAAAGAGAGATCTCCGTTATGAAGATGGTGAAACACC
GTAACATCGTTGAGTTACACGAAGTCATGGCGAGTAAATCGAAGATTTAC
TTTGCGATGGAGCTCGTGCGCGGCGGCGAGTTGTTTTCGAAGATCGCCAA
AGGTCGATTAAGAGAAGACGTGGCCAGAATGTATTTCCAGCAGTTAATCT
CCGCCATCGATTTCTGTCATAGCCGCGGCGTTTACCACCGCGATTTGAAG
CCGGAGAATCTCCTGCTAGACGAAGACGGTAATCTGAAGGTAACTGATTT
TGGATTAAGTGCTTTCTCCGAGCATCTAAAGCAGGATGGGCTTTTGCATA
CGACTTGTGGAACGCCTGCTTACGTGGCGCCGGAGGTCATCAGCAAAAAT
GGTTACGACGGCGCCAAATCGGATATTTGGTCCTGCGGCGTGATTCTTTA
CGTTCTCCTCGCCGGTTTTTTGCCGTTTCAAGACGATAACATCAACTTGA
CGAAAAAGCCATTCTACGACGAGATGTACGAGAAATCAAAACAGCCGGAA
ACTCTAAACGCGTTTCATATAATTTCATTATCGGAAGGGTTCGATCTGTC
GCCGTTGTTCGAGGAGAAGAAGAAAGAGGAGAAAGAAGAGATAAGATTCG
CGACGATGAGGCCTGCGAGTAGCGTGATTTCTAGATTCGAGGAGGTGGCT
AAGTCGGTGCAGTTCAATGTGAAGAAGAGCGATACAAGAGTGAGATTACA
AGGTAAAGAGAGCGGTAGAAAAGGGAAGCTGGCAATAAATGTCGATATAT
TCGCCGTAACGCCATCGTTCATGGTGGTGGAAGTAAAGAAGGATAACGGT
GACACTTTGGAGTATAACCAGTTCTGCAGTAAAGAACTGCGGCCCGCACT
TAAGGATATTGTCTGGATGTCAGCGACCGAGAATTCCACCATTAATGCTT
AA
5>NISI05G0518 [mRNA]
ATGGGGTTTGCAAACATCATAGGGAAGTATCATCTAGGCAGAACGATCGG
GGAAGGCAGTTTTGCCAAAGTGAAGCTGGGAGTAGATACAACAAATGTTA
TAGCAATAGATGTTTCACTCTTTCTAATAGCACTACGGAAACAGGTACAA
AGAGAGATAAGAATGATGAAGCTTCTACGTCATCCAAACATTGTACGTAT
AAACGAGGTTATTGGCACAAAGACGAAGATATATATAATAATGGAATATG
TATCTGGAGGACAACTCTCGGATAAGCTGTCTTATCTCAAGGAAATGAGT
GAAGCAGAAGCAAGAAAGGTCTTCCAGCAATTGATCGACGTGGTTGACTA
TTGCCATAACAGAGGAGTTTACCACAGAGATCTAAAGCCAGAAAACTTGC
TATTGGATGGTCAAGGAAATCTAAAAGTATCCGACTTTGGACTCAGTACT
TTGCGGCAGCCTGGAGATGTACTAACAACAGCCTGTGGCTCCCCATGTTA
CGTGGCACCAGAGCTGCTTGCAAGTAGAGGCTATGAAGGAGCAGCTGCAG
ATGTTTGGTCTTGTGGAGTAATCCTCTTTGAACTACTTGCTGGTTATCTG
CCATTCAATGACCGTAACCTTATGGTCTTGTATAGGAAGATAGCAGGAGC
AGAATACAGATTTCCACATTGGTTTACAGAAAGCCAGAAGAATCTAATCT
CCAGGATACTTAATCCAAATCCTAAGAAGAGAACGACAATACAAGAGATC
ATTGAGGATAAATGGTTTCAAACAGATTATGAACCTTCTTGTGGACGTGA
ATACGATGAGAAAATCTACTTGGACGATATTTATGCCGCTTTTGCAGTCA

AACGAGTTTTTAAACCATCACCTAATGACCTGAATTTGCAGCAGGAGAAT
GGTATGTCAAAATCGTCAAGTTTTATAAATGCATTCCAGTTAATAGCAAT
GTCACAAGACCTAGATTTGTCGGGACTGTTTGAAGGGCATGATGACAAGA
AGGAGAAAACAAGGCTTGGATCCAAGTTTCCAGTCAACGAAACAATAAAG
AAAATAGAAGCTGCAGCAATGGATGTGAGTCTAATGGTTGAGAGGACAAA
CAGCTTTAAAATGAAAATTCATCCAAAACAGAAGCAGAAGATGAGTAGAT
GCGCAAGATCATATTATGACCTCTCAGCAGAGAAGTTTATCAAGTCTGCT
AACAGAAAAACCCAATACGTCAAATCAAACACAAGAATTCAGTATAGGCA
GCGTCAATACAAGAAGTACACCAAAAATTGA
6>NISI06G1821 [mRNA]
ATGGTGGTGAGAAAAGTCGGAAAGTACGAGGTCGGAAGGACGATCGGGGA
AGGAACATTCGCCAAAGTGAAGTTCGCTCAAAACACGGAGACAGGGGAAA
GTGTTGCCATGAAAGTTCTCGATCGAAGTACCATAATCAAACACAAGATG
GCTGATCAGGTTCTCGCTAGTCGTACTAAGATTTACATTATCTTGGAGTT
CATCACAGGCGGTGAATTGTTTGATAAGATAGTTCATCACGGACGTCTTA
GCGAGGCTGAAGCTAGGACGTATTTCCAACAGCTTATTGATGGTGTAGAT
TTTTGTCACAGTAAGGGTGTCTACCACAGAGATTTGAAGCCTGAAAATCT
TTTACTTGATTCTCAAGGAAATCTAAAGATATCAGATTTTGGTCTAAGTG
CATTCCCTGAGCAAGAAAATAGCCTACTTCGCACCACATGTGGGACGCCT
AACTATGTAGCACCTGAGGTGCTAAGTCACAAGGGTTATAATGGTGCTGT
GGCTGATGTGTGGTCTTGTGGGGTCATCCTTTATGTTTTAATGGTTGGAT
ATCTTCCGTTTGATGAGCTTGATCTCACCACTCTGTACAAAGCTAGTTTT
GTGATAGACAGTAGATTAGATACTGGGAGTTTATATCATTATGAAACTCG
TGGTTTTCTGCAGGTTGAGAAAGCAGAATTTTCATGCCCATCTTGGTTCC
CAGTGGGGGCAAAATCTTTGATCCATAGAATATTGGACCCAAATCCTGAA
ACTCGTATTACCATTGAACAGATAAGGAATGATGAGTGGTTTCAGAAGGG
TTATGTTCCTATGAGACTTCCAGAACACGAGGATGTGAACTTGGACGATA
TAAATGCTGTTTTTGATGATCCCGAGGTTGGTAAGGAAGGGCAGGCAAAC
GAGCAATGTGGAAGTGGGACAGAGGACATGGGTCCTTTAATTCTCAATGC
GTTTGACTTGATTATTTTATCTCAAGGCTTAAACCTCGCATCACTTTTTG
ATCGTGGGAAGGAAACTGTGAAGCATCAGACCCGCTTCATTTCACAGAAG
CCGGCGAAGGTTGTTTTATCAAGTATGGAGGTTGTTGCACAATCCATGGG
TTTTAAGACACATATTCGCAATTATAAGATGAGAGTTGAAGGCCTTTCTG
CAGATAAAGCTGGTCATTTCTCTGTCATCCTGGAAATTTTTGAAGTGGCA
CCGACGTTTTTTATGGTGGACATTCAGAAAGCAGCTGGAGATGCAAGTGA
ATACCACAAGTTTTACAAAAACTTTTGTAGCAATCTTGAGGATATCATCT
GGAAACCCCCAAATGAACCATGCAAATCAAGGATCACCAAGTCAAAGAGT
AGAAAGCGTTGA
7>NISI07G1958 [mRNA]
ATGGAATACAAAGGGAAGGTGCTGATGGAGAAGTATGAGTTGGGGAGATT
GTTGGGCCAGGGGACCTTTGCTAAGGTTTACTTTGCTAGGAATCTTGAAA
CCAGCCAAAGTGTAGCCGTTAAGGTTATTGACAAGGAGAAGATCCTTAAA
GCAGAATTGACTGAGCAAACCCTAACAGAAGTATCTGTTATGAGACTAAT
CAAACATCCAAATGTGTTGCAACTTTATGAGGTCATGGCCACCAAGACCA

AGATTTACTTTGTCATGGAATATGCCAAAGGTGGTGAGCTCTTTAACAAG
CTAGCAAAAGGAAGGCTTAGAGAAGACAGAGCAAGGAAGTATTTCCAGCA
GTTGATAAGTGCTATTGATTTTTGCCACAGCAGAGGTGTATACCACCGTG
ATCTAAAACCAGAGAACTTACTATTGGATGAGAATGGAACACTAAAGGTT
TCTGATTTTGGGTTGAGTGCTCTCTCTGATTCGAAGAAACAAGATGGGTT
GCTTCACACTACTTGTGGAACCCCTGCCTATGTTGCTCCTGAAATCATTC
GTAGAAAAGGCTATGATGGAGCAAAATCAGATATCTGGTCTTGTGGGGTT
ATCTTGTATGTTCTTTTAGCCGGTTATCTCCCATTCCATGATTCAAATTT
GATGGCCATGTATAGAAAAATCAGCAAGGGAGACTATGTGATCCCTAGTT
GGTTTTCAACAGATGTGCGAAGATTGCTGACGAGAATCCTCGACCCTAAC
CCAAAAACCAGGGTTTCCCTTGCTAAAGTTATGGAAAACCCATGGTTTAA
GAAAGGATTGAATTCAAAACCCCTTGAAGCTCAAACAGAAGTTATTGAGA
ACGTACCTTTGGATGGTGATACAACTTTGGTTCCTCCTGAAAATGGTGCT
ATTAACGATCCAAAGAGAGAGTTGATTAAACCTGCTAAGATGAATGCATT
TGATATCATCTCTCTTTCAACTGGGTTTGATTTATCTGGTTTGTTTGTGG
ACAACAACCAGAAGGAGGAAGCAAAATTCACGACGACACTCTCAGCCTTA
GATATTATATCTAAACTAGAAGGTATTGCTAGAGATCTGAGGCTGCAAGT
AACAAAGAAGGACAGAGGGATGTTAAAATTGGAGAGACCAAAGGAAGGTA
GAAGGGGGGGGTTGTCCATTGATGCAGACATATATGAGTTGAGTCCTTCC
TTTCATTTGGTTGAAATGAAGAAGTCTGCTGGTGACACTCTGGAATATCA
TATCATGTTGAAACAAGACATTAGACCAGCTCTCAAGGATATTGTTTGGG
CTTGGCAAGGGGAGAAGCCCCTGCTACAACAATACCATCGGCACTTTTTT
TAG
8>NISI09G0114 [mRNA]
ATGAGTCAGCCTAAAATAAAGCGTAGGGTGGGTAAATACGAGGTTGGCAG
AACCATAGGCGAAGGAACGTTTGCAAAGGTGAGGTTTGCTAGGAATTCTG
AGACTGGGGAAGCCGTAGCTCTCAAGATTCTTGATAAAGAGAAGGTTCTT
AAGCACAAGATGGCTGAACAGATCAAGCGGGAAATTGCAACGATGAAGTT
AGTAAAGCACCCAAATGTTGTTCAGTTGTATGAGGTGATGGCAAGCAAAA
CGAAGATATTCATAGTGTTGGAGTTTGTCACTGGAGGAGAGCTCTTTGAC
AAAATTGTAAACCATGGACGGATGAGAGAAGATGAGGCACGTAGATATTT
CCAGCAGCTTATAAATGCCGTTGATTATTGCCATAGCCGAGGTGTTTATC
ATAGAGACCTCAAGCCGGAGAATCTGCTTTTGGATGCCTATGGAAACCTT
AAAGTTTCTGATTTCGGATTGAGCGCTTTGTCTCAACAATTGAGGAATGA
TGGCCTTCTCCACACAACCTGTGGAACTCCAAACTACGTTGCTCCAGAGG
TTCTGAATGATAGAGGATATGATGGGGCAACCGCAGATTTGTGGTCGTGT
GGAGTCATACTCTTTGTACTGCTTGCTGGGTACTTGCCTTTTGATGATAA
TAATCTTATGACCCTTTATAAAAAAATCTCTGCTGCTGAATTCACATGCC
CCTCATGGCTCTCTCTTCCTGCCATGAAATTGATATCTCGTATCTTGGAT
CCCAACCCCGTGACACGAATTTCTATTGCGGAAATTCTGGAAGATGAATG
GTTTAAGACAGATTATAAAACCCCAGTGTTCGTAGAGAAAGAACATGCCA
ATGTGGATGATGTTGAAGCTGTTTTTAAGGATTCGGAAGAGCATCATGTA
ACAGAGAGGAAAGAAGAACAGCCAGTGGCAATGAATGCCTTCGATTTGAT
TTCTTTGTCAAAGGGTCTAAACCTTGGCAATCTGTTTGATGTAGCACAGG

GATTCAAGAGGGAAACAAGGTTCACATCCAAACGTCCAGCTAATGAGATA
ATCAGTAAAATTGAAGAAGCGGCTAAGCCTCTTGGGTTTGATGTTCATAA
GAAAAACTACAAGATGAGGCTTCAAAATGTGAAAGCGGGAAGAAAGGGAA
ATCTTAATATAGCGACAGAGATATTTCAAGTGGCACCCTCTCTTCATATG
GTCGAGGTGAGAAAGGCAAAAGGCGACACATTGGAGTTTCATAAGTTCTA
TAAGAATCTGTCAAGCAGACTGGAGGGAGTAGTGTGGAAAACAGAAGAGC
AAATGCAAGAAATGGAGTAA
9>NISI09G0120 [mRNA]
ATGAGTCAGCCTAAAATAAAGCGTAGGGTGGGTAAATACGAGGTTGGCAG
AACCATAGGCGAAGGAACGTTTGCAAAGGTGAGGTTTGCTAGGAATTCTG
AGACTGGGGAAGCCGTAGCTCTCAAGATTCTTGATAAAGAGAAGGTTCTT
AAGCACAAGATGGCTGAACAGATCAAGCGGGAAATTGCAACGATGAAGTT
AGTAAAGCACCCAAATGTTGTTCAGTTGTATGAGGTGATGGCAAGCAAAA
CGAAGATATTCATAGTGTTGGAGTTTGTCACTGGAGGAGAGCTCTTCGAC
AAAATTGTAAACCATGGACGGATGAGAGAAGATGAGGCACGTAGATATTT
CCAGCAGCTTATAAATGCCGTTGATTATTGCCATAGCCGAGGTGTTTACC
ATAGAGACCTCAAGCCGGAGAATCTGCTTTTGGATGCCTATGGAAACCTT
AAAGTTTCTGATTTCGGATTGAGTGCTTTGTCTCAACAATTGAGGAATGA
TGGCCTTCTCCACACAACCTGTGGAACTCCAAACTACGTTGCTCCAGAGG
TTCTGAATGATAGAGGATATGATGGGGCAACCGCAGATTTGTGGTCGTGT
GGAGTCATACTCTTTGTACTGCTTGCTGGGTACTTGCCTTTTGATGATAA
TAATCTTATGACCCTTTATAAAAAAATCTCTGCTGCTGAATTCACATGCC
CCTCATGGCTCTCTCTTCCTGCCATGAAATTGATATCTCGTATCTTGGAT
CCCAACCCCGTGACTCGAATTTCTATTGCGGAAATTCTCGAGGATGAATG
GTTTAAGACAGATTATAAAACCCCAGTGTTCGTAGAGAAAGAACATGCCA
ATGTGGATGATGTTGAAGCTGTTTTTAAGGATTCGGAAGAGCATCATGTA
ACAGAGAGGAAAGAAGAACAGCCAGTGGCAATGAATGCCTTCGATTTGAT
TTCTTTGTCAAAGGGTCTAAACCTTGGCAATCTGTTTGATGTAGCACAGG
GATTCAAGAGGGAAACAAGGTTCACATCCAAACGTCCAGCTAATGAGATA
ATCAGTAAAATTGAAGAAGCGGCTAAGCCTCTTGGGTTTGATGTTCATAA
GAAAAACTACAAGATGAGGCTTCAAAATGTGAAAGCGGGAAGAAAGGGAA
ATCTTAATATAGCGACAGAGATATTTCAAGTGGCACCCTCTCTTCATATG
GTCGAGGTGAGAAAGGCAAAAGGCGACACATTGGAGTTTCATAAGTTCTA
TAAGAATCTGTCAAGCAGACTGGAGGGAGTAGTGTGGAAAACAGAAGAGC
AAATGCAAGAAATGGAGTAA
10>NISI10G0044 [mRNA]
ATGACGTCTCGGACGGGCGGAGCGAGGACGCGTGTGGGAAAGTACGAGTT
AGGGAGGACACTGGGAGAAGGGAGCTTCGCGAAGGTGAAGTTTGCCAGAC
ACACTGAAACAGGGGAGAATGTCGCCATCAAAATCCTTGACAAAGATAAA
GTTCTTAGGCATAAGATGATCGGTCAGATTAAACGCGAAATTTCTACCAT
GAAGCTCATTAGACATCCAAATGTCATCCGTATGTATGAGGTGATGGCAA
GCAAGACAAAAATATATATTGTTCTAGAATTTGTCACTGGTGGTGAACTT
TTTGACAAAATTGCTAGTAGAGGCAGGTTGAAGGAAGATGAAGCTAGAAA
ATATTTTCAGCAGCTTATTAATGCTGTGGATTACTGCCATAGCAGAGGTG

TTTATCATAGGGACTTAAAGCCTGAGAATTTACTATTGGATGCTAATGGA
GTATTGAAGGTTTCAGATTTTGGGTTGAGTGCTTTACCTCAGCAAGTTAG
AGAAGATGGTTTACTTCACACAACATGTGGAACACCTAATTATGTTGCTC
CTGAGGTAATCAATAATAAAGGTTACGACGGCGCTAAGGCAGATTTGTGG
TCATGTGGTGTCATTCTTTATGTCTTAATGGCTGGTTATTTGCCTTTTGA
AGATTCCAATCTCATGAATTTATATAAAAAGATATTTAAGGCTGACTTCA
AGTTCCCTCCTTGGTTCTCATCAAGTGCAAAGAAACTAATCACAAGAATC
CTGGACCCTAATCCTTCAACTCGGATAACAATTGCCGAGGTCATTGAGAA
TGAGTGGTTTAAGAAGGGATATAAGCCCCCTGTTTTTGAACAACCTGATG
TTAGTCTTGTTGACGTGGATGCTATCTTTAATGAATCGGTGGATTCTCAA
AACCTTGTGGTGGAAAGGCGTGAAGAAGGGCCTAGAGCACCGGTGACTAT
GAATGCATTTGAGCTGATTTCCACATCTCAGGGTCTCAACCTGAGTACTC
TTTTTGAAAAACAAATGGGACTTGTCAAACGGGAGACAAGGTTTACTTCC
AAATGTCCTGCTAATGAAGTAATATCAAAAATAGAGGAAGCTGCACAACC
TTTGGGATTTGATGTGAAGAAAAGCAATAACAAGATGAAACTTCAAGGGG
AAAAAACTGGACGGAAAGGTCATTTATCTGTTGCAACTGAGATTTTTCAG
GTGGCCCCTTCACTTTGCATGGTTGAACTGCGAAAGTCTGGAGGAGATAC
TCTAGAATTTCACAAGTTCTACAAGAACCTCACGACAGGGCTAAAAGATA
TTGTTTGGAAGCCAGCTGATGAAATAAAGGAGGAGTGA
11>NISI10G0902   [mRNA]
ATGGCAAGCTCGGCCGCCGCGAGCGCCAACGTCGGTGGCAAAGAGCAGCA
GAACCAGAGCCCGCTTCTCGGCCGCTATGAGGTCGGGAGACTTCTAGGCC
ATGGGACTTTTGCGAAAGTCTACCAGGCTAAGAACGTCAAGACGGGAGAA
GGCGTCGCGATCAAGGTGATCGACAAGGAAAAGATATTGAAGAGTGGACT
AATCGCTCATATCAAGCGGGAGATCTCAATTCTGCGTATGGTACGCCATC
CGAACATCGTGCAGCTCTTCGAGGTTATGGCGACCAAGTCGAAGATCTAC
TTCGTCATGGAGTATGTTCGCGGCGGTGAACTTTTTAACAAAGTCGCCAA
AGGAAGATTAAAGGAAGATCTCGCGAGGAAATACTTCCAGCAGCTAATTT
CGGCAGTAGGGCTTTGCCATGCGCGTGGAGTCTACCACCGCGACCTGAAA
CCTGAAAATTTGCTGCTGGACGATAACGGCGATTTGAAGGTTTCCGACTT
CGGACTTAGTGCTGTGTCCGATCAGATTCGGCAAGACGGTTTGTTTCATA
CTTTTTGTGGAACTCCGGCGTATGTTGCTCCGGAGGTTTTAGCTAGGAAA
GGATACTGTGCGGCCAAAGTAGACATTTGGGCGTGCGGTGTCATATTGTT
TGTTTTAATGGCCGGATATTTACCGTTTCACGACCAAAACATAATGTCTA
TGTATAAAAAGATATATAAGGGTGAATTTAGGTGCCCTAAATGGTTTTCG
CCAGATTTAGTCAGGCTTATGAAGAGGCTTCTCGCTACAAACCCTGATAC
TAGAATTACCATTCCTGAAATTATGGAGAATAGGTGGTTTAAAAAAGGGT
TTAAGCATATTAAATTCTATATAGAGGATGACAAGCTCTGTAATGTTGTT
GATGATGATGCCGATGTGGAGTCATTATCTGAGCAGTCAATGTCTGAATC
AGATTCTGAATTGGAGACCAGAAGGAAAGTTACTTCTTTGCCCAGACCGG
CAAGTTTGAATGCATTTGATATTATATCGTTTTCTCCTGGGTTTGATTTA
TCGGGACTATTTGAAGAAGGAGGAGGAGCAAGATACGTGTCTGGTGCTCC
AGTTTCAAAAATTATATCAAAATTGGAGGAGATTGCTAAGGTGGTGAGTT
TTACAGTGAGGAAGAAGGATTGTAGAGTGAGTTTGGAAGGGTCTAGGGAA

GGTACGAAGGGGCCATTAACTATAGCGGCTGAAATTTTTGAGTTAACATC
TAAGCTGGTGATGGTGGAGGTGACCAAAAAAGGAGGGGATAGAGGTGAGT
ATGAGGAGTTTTGTAATAAGGAATTGAAACCTGGGTTGGATAATTTAATG
GCCGAGGAATCAGAACCTGCTGCTGTTCTGATTTGCATCTGCCATCAGAT
ACAGAATAAGGAGTGGGAGAAGGAATCAAAAACGGGAGCAAAGGGAAACA
CATTGGTGCTTGGACTGCTGAATCCTGAAGCAAACAGAAGGTGCTCTCAC
AAAGCTCGAGTTTTGATTCCTCTGAGACACCTGCCTGAACGAGAAACGCA
CAACCCTCTCTTTTCCCGAAGATCAAAGAGTTCTTCTGCGTTTCGTCGGT
TGCTAAACAGGTTCAGACATAGCTGCAGGTCATCAAGAAAACTGGCGAAG
GAGGTGGTGGGTCTTGATTTTCAACCTATGTATGTAGTCCTATTGAATAA
GAGACCAAAGTATATGTAA
12>NISI11G0565 [mRNA]
ATGAATAAGGTACCGGGGACGAGGACACGTGTGGGGAAATATGAAATAGG
AAGGACAATTGGTGAGGGTAGCTTTGCCAAGGTGAAATTCGCCAAGATTG
AGACCGGAGAGTTTTTCGCCATTAAAGTGCTCGACCGTGATCAAGTCCTC
CGTCACAAGATGGTCGAACAGATAAAGAGAGAGATATCAACAATGAAGCT
GATCAAACATCCTAATGTCGTGAAAATGATTGAGGTTATGGCAAGCAAAA
CAAAGATCTACATTGTTCTCGAGTTTGTCGATGGGGGTGAGCTCTTTGAT
AAAATTGCAAGGAGTGGGAAACTCAAAGAAGATGAAGCAAGGAGATATTT
CCACCAGCTCATTAATGCTGTGGACTATTGTCACAGTAGAGGGGTGTTCC
ACAGAGATTTGAAGCCGGAGAATCTTCTTCTTGACAGATCTGGCGCTCTG
AAAATTTCAGATTTCGGTTTAAGTGCGCTGTCGCAGCAAGTGCGGGAAGA
TGGGCTGCTTCACACAGCTTGTGGGACTCCAAATTATGTTGCTCCTGAGG
TGCTTAATGACAAAGGCTATGATGGTACTGCATCGGATGTTTGGTCCTGT
GGAGTCATTCTCTTTGTCCTGATGGCAGGATACTTACCTTTTGACGAGCC
AAGTCTAATGTCCTTATATAGAAAAATATGCAAGGCTGAGTTCTCTTGTC
CATCATGGTTCTCACCTGGTGCTAAGAAATTGATCAAGCGTATTCTTGAC
CCAAATCCTCATACTCGAATTACTATTTCGGAAATATTAGAGGATGAATG
GTTTAAGAAGGGGTACAAGCCACCACAATTTGATAAGGAGGAAGATGTTA
ATCTAGATGATGTGGATGCCGTTTTCAATGACTCGAAGGAATATCTTGTA
ACAGAAAGGAAGGAGAAACCTGTATCAATGAATGCTTTTGAGCTAATCTC
GAGGTCACAGAGTTTTAACCTCGAGAACTTATTTGAGAAGCAGACGGGTC
TTGTGAAGCGAGAAACGCGTTTTACTTCCCAACGCCCGGCAAATGAGATC
ATGTCTAAAATTGAGGAAACTGCAAAGCCTTTGGGCTTCAATGTTCGCAA
AGGAAACTATAAGATGAAGTTGCAAGGTGACAAAAGTGGAAGGAAAGGCC
AGCTCTCTGTAGCTACTGAGGTGTTTGAGGTGGCTCCCTCTGTGCACATG
GTGGAGGTCCGTAAAACTGGTGGCGACACACTAGAATTTCACAAGTTCTA
CAAAACTTTCTCATCAGGACTGAAAGATGTAGTCTGGCAAACAGAAGAAA
ATGACGAAAAAGCTGTAAAGAAATCACGTTAG
13>NISIScaf80G0007 [mRNA]
ATGTTCAACAAAAAGTTAGGGGTGGGAGCAACTGCAACTGCAAATACAAC
TACAACTACAACTGAGGAGGATCCCAACAAGCAAGGAATGCGTCTTGGAA
AGTACCAACTTGGAAGGACTCTCGGGGAAGGCAATTTCGGTAAAGTTAAA
TACGCTAAGAAATTGGATTCTGGCCTACCCTTTGCTGTCAAAATTCTTGA

GAAGACCAAGATCTTCGACCTCAAAATCACTGATCAGGTCTTGGCAAGCA
AAACAAGGATTTATATGGTGCTAGAATACGTAACTGGTGGAGAGTTGTTT
GACAGAATTGATGACGGTTTACTGCATACAACTTGTGGAAGTCCAAACTA
TGTTGCACCTGAGGTCCTTTCCAATAGAGGGTATAACGGTGCCACCTCAG
ATATCTGGTCAAGCGGTGTTATCCTATACGTAATTCTCACTGGACACCTC
CCTTTCGATGATAGGAATCTTGCTGTTCTCTATCAAAAGATTTTCAAGGG
AGATGCTAAGATACCAAAATGGTTATCTCCTGGCGCCCAAAACATATTAA
GAAGGATTTTGGATCCAAATCCTGTTACTCGTTTGACCATGTCTCAAATC
AAATCTGATGAATGGTTCAAGCAGGGGTACACTCCTGCAGATCCCACTGA
AGAAGAAGAAGAAGATATAATCATCGATGATGAAGCCTTCTCAATTCATG
AATTGCATTCTGATGGGGAAAAGACACCAGATTCACCTGGCACCATCAAT
GCTTTCCAGTTGATTGGAATGTCATCATGCCTTGACCTCTCTGGTTTCTT
TGAGAAAGAGGATGTCTCTGAGAGGAAGATCAGATTCACATCCAATCTCT
CCATGAAAGATCTGTTGGCGAGGATTGATAGTATTGTGACAGAGATGGGA
TTTCGAGTCCAAAAGAAAAACGGAAGGTTGAAAGCCACGCAAGATCACAA
GGGACAGAGTAGCTTAGGAAGTCTTTCAGTGGCAGCAGAGGTATTTGAGA
TTAGCCCATCTCTATATGTGGTTGAATTGAGAAAATCATACGGAGACTCT
GCTGTATAG
14>NISIScaf97G0002 [mRNA]
ATGTTCAACAAAAAGTTAGGGGTGGGAGCAACTGCAACTGCAAATACAAC
TACAACTACAACTGAGGAGGATCCCAACAAGCAAGGAATGCGTCTTGGAA
AGTACCAACTTGGAAGGACTCTCGGGGAAGGCAATTTCGGTAAAGTTAAA
TACGCTAAGAAATTGGATTCTGGCCTACCCTTTGCTGTCAAAATTCTTGA
GAAGACCAAGATCTTCGACCTCAAAATCACTGATCAGATAAAGAGGGAAA
TTGGGACTTTGAAGCTCCTTAAGCATCCGAATGTCGTCCGATTACATGAG
GTCTTGGCAAGCAAAACAAGGATTTATATGGTGCTAGAATACGTAACTGG
TGGAGAGTTGTTTGACAGAATTGATGACGGTTTACTGCATACAACTTGTG
GAAGTCCAAACTATGTTGCACCTGAGGTCCTTTCCAATAGAGGGTATAAC
GGTGCCACCTCAGATATCTGGTCAAGCGGTGTTATCCTATACGTAATTCT
CACTGGACACCTCCCTTTCGATGATAGGAATCTTGCTGTTCTCTATCAAA
AGATTTTCAAGGGAGATGCTAAGATACCAAAATGGTTATCTCCTGGCGCC
CAAAACATATTAAGAAGGATTTTGGATCCAAATCCTGTTACTCGTTTGAC
CATGTCTCAAATCAAATCTGATGAATGGTTCAAGCAGGGGTACACTCCTG
CAGATCCCACTGAAGAAGAAGAAGAAGATATAATCATCGATGATGAAGCC
TTCTCAATTCATGAATTGCATTCTGATGGGGAAAAGACACCAGATTCACC
TGGCACCATCAATGCTTTCCAGTTGATTGGAATGTCATCATGCCTTGACC
TCTCTGGTTTCTTTGAGAAAGAGGATGTCTCTGAGAGGAAGATCAGATTC
ACATCCAATCTCTCCATGAAAGATCTGTTGGCGAGGATTGATAGTATTGT
GACAGAGATGGGATTTCGAGTCCAAAAGAAAAACGGAAGGTTGAAAGCCA
CGCAAGATCACAAGGGACAGAGTAGCTTAGGAAGTCTTTCAGTGGCAGCA
GAGGTATTTGAGATTAGCCCATCTCTATATGTGGTTGAATTGAGAAAATC
ATACGGAGACTCTGCTGTATATAGACAGTTGTGCAAACGAATATCAAGTG
ATTTAGGCGTCCCCTCAAGCCAAGGGTTGGTTGCTACACAAGTGTGA

1>NISI03G1060.1 [protein]
METMGSILMERYELGRLLGQGTFAKVHYARNLKTGMSVAIKIIDKERVIR
YGLMNQTKREISVMRQIKHPNVVELYEVMATKTKIYFVIEYVKGGELFNR
LANGKLNEDAARKYFQQLISAVDYCHSRGVYHRDIKPENLLLDENENLKV
SDFGLSALVDSKRKDGLLHTTCGTPAYVAPEVINRKGYDGSKADIWSCGV
VLYVLLAGHLPFHDSNLMEMYRKIGRADFKYPNWFAADVRRLITKILDPN
LVTRISMDKIMESSWFKKGLEKPITIDLEGKPEIPADTDATFTLNESSGG
RTVPKAENELTKPSNLNAFDIISFSTGFDLSGLFEDREERKEMRFTSNKP
AATIISKLQDIAKRLKLKVKKKDGGLLKMEGPKEGWKGVLGIDAEIFEIT
PFLHMVEMKKSSGDTLEYQKMMKQEIRPALKDIVWTWQGEQLQQQCSEHI
ALPSPVQQETTA
2>NISI03G1063.1 [protein]
MEERTVDGGEVLGGGNLFEKYELGRLVGRGAFAKVYHGRDIRTGQSVAIK
AVGKNKVVKEGFMAHIKREISIMRRLRHPHVVKLLEVMATKTKVYFVMEF
AKGGELFTKVSKGRFSEDLSRKYFQQLISTVGYCHSRGVFHRDLKPENLL
LDENWDLKITDFGLSALKDQTRSDGMLHTLCGTPAYVAPEILAKKGYDGA
KVDIWSCGVVLFVLNSGYLPFNDHNIMVMYRKIYKGEFRCPKWTSPDLKH
LLSRLLDPNPDTRITIDEILKDPWFRKGYKEVNFHSEDSDLKDLGVNDNR
KCLNAFDIITFSPGFSLTGLFNDYTDAERFLSAEKPDKIIEKVEEMAKKE
NLTVMTKKGYGIKLEGHDGNFSLVTAVHRLTDKLVIVDIKKREREVGSGQ
EFWKNNLRPRLRGLIYPPETPVADNS
3>NISI03G1369.1 [protein]
MPEIEHVPADYDRNCNAADGALFGKYELGKLLGCGAFAKVYHARDVRTNQ
SVAIKIISKKKINVNLMSNIKREISIMRRLNHRHIVKLHEVLASKTKIYF
VVEFAKGGELFAKVAKGRFSEDLSRKYFQQLISAVGYCHSRGVYHRDLKP
ENLLIDENGNLKVSDFGLSALTDQIRTDGLLHTLCGTPAYVAPEILSKKG
YDGAKVDIWSCGVILFVLTAGYLPFNDPNLMAMYKKIYKGEFRCPKWMSN
DLKRLLNRLLHINPNTRITVDQILGDPWFRRGGVKEIKFHDDENAAVPDK
TGKEGFGARNLNAFDIISFSSGLDLSGLFDTSCNSFENNTGERFISRESP
DNLLETVTEFAKVEKLRLKTRKEWGVELEEQNGNFIIGVDVYRLTEELVV
VEANRRAGDAASYTEVWKNKLRPQLLVRQQEASVSGNH
4>NISI05G0308.1 [protein]
MAAAAVIAAVEKSTSRDRSTLLHGKYELGRLLGHGTFAKVYHARHLQTGR
SVAMKVVGKEKVIKVGMMEQIKREISVMKMVKHRNIVELHEVMASKSKIY
FAMELVRGGELFSKIAKGRLREDVARMYFQQLISAIDFCHSRGVYHRDLK
PENLLLDEDGNLKVTDFGLSAFSEHLKQDGLLHTTCGTPAYVAPEVISKN
GYDGAKSDIWSCGVILYVLLAGFLPFQDDNINLTKKPFYDEMYEKSKQPE
TLNAFHIISLSEGFDLSPLFEEKKKEEKEEIRFATMRPASSVISRFEEVA
KSVQFNVKKSDTRVRLQGKESGRKGKLAINVDIFAVTPSFMVVEVKKDNG
DTLEYNQFCSKELRPALKDIVWMSATENSTINA
5>NISI05G0518.1 [protein]
MGFANIIGKYHLGRTIGEGSFAKVKLGVDTTNVIAIDVSLFLIALRKQVQ
REIRMMKLLRHPNIVRINEVIGTKTKIYIIMEYVSGGQLSDKLSYLKEMS
EAEARKVFQQLIDVVDYCHNRGVYHRDLKPENLLLDGQGNLKVSDFGLST

LRQPGDVLTTACGSPCYVAPELLASRGYEGAAADVWSCGVILFELLAGYL
PFNDRNLMVLYRKIAGAEYRFPHWFTESQKNLISRILNPNPKKRTTIQEI
IEDKWFQTDYEPSCGREYDEKIYLDDIYAAFAVKRVFKPSPNDLNLQQEN
GMSKSSSFINAFQLIAMSQDLDLSGLFEGHDDKKEKTRLGSKFPVNETIK
KIEAAAMDVSLMVERTNSFKMKIHPKQKQKMSRCARSYYDLSAEKFIKSA
NRKTQYVKSNTRIQYRQRQYKKYTKN
6>NISI06G1821.1 [protein]
MVVRKVGKYEVGRTIGEGTFAKVKFAQNTETGESVAMKVLDRSTIIKHKM
ADQVLASRTKIYIILEFITGGELFDKIVHHGRLSEAEARTYFQQLIDGVD
FCHSKGVYHRDLKPENLLLDSQGNLKISDFGLSAFPEQENSLLRTTCGTP
NYVAPEVLSHKGYNGAVADVWSCGVILYVLMVGYLPFDELDLTTLYKASF
VIDSRLDTGSLYHYETRGFLQVEKAEFSCPSWFPVGAKSLIHRILDPNPE
TRITIEQIRNDEWFQKGYVPMRLPEHEDVNLDDINAVFDDPEVGKEGQAN
EQCGSGTEDMGPLILNAFDLIILSQGLNLASLFDRGKETVKHQTRFISQK
PAKVVLSSMEVVAQSMGFKTHIRNYKMRVEGLSADKAGHFSVILEIFEVA
PTFFMVDIQKAAGDASEYHKFYKNFCSNLEDIIWKPPNEPCKSRITKSKS
RKR
7>NISI07G1958.1 [protein]
MEYKGKVLMEKYELGRLLGQGTFAKVYFARNLETSQSVAVKVIDKEKILK
AELTEQTLTEVSVMRLIKHPNVLQLYEVMATKTKIYFVMEYAKGGELFNK
LAKGRLREDRARKYFQQLISAIDFCHSRGVYHRDLKPENLLLDENGTLKV
SDFGLSALSDSKKQDGLLHTTCGTPAYVAPEIIRRKGYDGAKSDIWSCGV
ILYVLLAGYLPFHDSNLMAMYRKISKGDYVIPSWFSTDVRRLLTRILDPN
PKTRVSLAKVMENPWFKKGLNSKPLEAQTEVIENVPLDGDTTLVPPENGA
INDPKRELIKPAKMNAFDIISLSTGFDLSGLFVDNNQKEEAKFTTTLSAL
DIISKLEGIARDLRLQVTKKDRGMLKLERPKEGRRGGLSIDADIYELSPS
FHLVEMKKSAGDTLEYHIMLKQDIRPALKDIVWAWQGEKPLLQQYHRHFF
8>NISI09G0114.1 [protein]
MSQPKIKRRVGKYEVGRTIGEGTFAKVRFARNSETGEAVALKILDKEKVL
KHKMAEQIKREIATMKLVKHPNVVQLYEVMASKTKIFIVLEFVTGGELFD
KIVNHGRMREDEARRYFQQLINAVDYCHSRGVYHRDLKPENLLLDAYGNL
KVSDFGLSALSQQLRNDGLLHTTCGTPNYVAPEVLNDRGYDGATADLWSC
GVILFVLLAGYLPFDDNNLMTLYKKISAAEFTCPSWLSLPAMKLISRILD
PNPVTRISIAEILEDEWFKTDYKTPVFVEKEHANVDDVEAVFKDSEEHHV
TERKEEQPVAMNAFDLISLSKGLNLGNLFDVAQGFKRETRFTSKRPANEI
ISKIEEAAKPLGFDVHKKNYKMRLQNVKAGRKGNLNIATEIFQVAPSLHM
VEVRKAKGDTLEFHKFYKNLSSRLEGVVWKTEEQMQEME
9>NISI09G0120.1 [protein]
MSQPKIKRRVGKYEVGRTIGEGTFAKVRFARNSETGEAVALKILDKEKVL
KHKMAEQIKREIATMKLVKHPNVVQLYEVMASKTKIFIVLEFVTGGELFD
KIVNHGRMREDEARRYFQQLINAVDYCHSRGVYHRDLKPENLLLDAYGNL
KVSDFGLSALSQQLRNDGLLHTTCGTPNYVAPEVLNDRGYDGATADLWSC
GVILFVLLAGYLPFDDNNLMTLYKKISAAEFTCPSWLSLPAMKLISRILD
PNPVTRISIAEILEDEWFKTDYKTPVFVEKEHANVDDVEAVFKDSEEHHV

TERKEEQPVAMNAFDLISLSKGLNLGNLFDVAQGFKRETRFTSKRPANEI
ISKIEEAAKPLGFDVHKKNYKMRLQNVKAGRKGNLNIATEIFQVAPSLHM
VEVRKAKGDTLEFHKFYKNLSSRLEGVVWKTEEQMQEME
10>NISI10G0044.1 [protein]
MTSRTGGARTRVGKYELGRTLGEGSFAKVKFARHTETGENVAIKILDKDK
VLRHKMIGQIKREISTMKLIRHPNVIRMYEVMASKTKIYIVLEFVTGGEL
FDKIASRGRLKEDEARKYFQQLINAVDYCHSRGVYHRDLKPENLLLDANG
VLKVSDFGLSALPQQVREDGLLHTTCGTPNYVAPEVINNKGYDGAKADLW
SCGVILYVLMAGYLPFEDSNLMNLYKKIFKADFKFPPWFSSSAKKLITRI
LDPNPSTRITIAEVIENEWFKKGYKPPVFEQPDVSLVDVDAIFNESVDSQ
NLVVERREEGPRAPVTMNAFELISTSQGLNLSTLFEKQMGLVKRETRFTS
KCPANEVISKIEEAAQPLGFDVKKSNNKMKLQGEKTGRKGHLSVATEIFQ
VAPSLCMVELRKSGGDTLEFHKFYKNLTTGLKDIVWKPADEIKEE
11>NISI10G0902.1 [protein]
MASSAAASANVGGKEQQNQSPLLGRYEVGRLLGHGTFAKVYQAKNVKTGE
GVAIKVIDKEKILKSGLIAHIKREISILRMVRHPNIVQLFEVMATKSKIY
FVMEYVRGGELFNKVAKGRLKEDLARKYFQQLISAVGLCHARGVYHRDLK
PENLLLDDNGDLKVSDFGLSAVSDQIRQDGLFHTFCGTPAYVAPEVLARK
GYCAAKVDIWACGVILFVLMAGYLPFHDQNIMSMYKKIYKGEFRCPKWFS
PDLVRLMKRLLATNPDTRITIPEIMENRWFKKGFKHIKFYIEDDKLCNVV
DDDADVESLSEQSMSESDSELETRRKVTSLPRPASLNAFDIISFSPGFDL
SGLFEEGGGARYVSGAPVSKIISKLEEIAKVVSFTVRKKDCRVSLEGSRE
GTKGPLTIAAEIFELTSKLVMVEVTKKGGDRGEYEEFCNKELKPGLDNLM
AEESEPAAVLICICHQIQNKEWEKESKTGAKGNTLVLGLLNPEANRRCSH
KARVLIPLRHLPERETHNPLFSRRSKSSSAFRRLLNRFRHSCRSSRKLAK
EVVGLDFQPMYVVLLNKRPKYM
12>NISI11G0565.1 [protein]
MNKVPGTRTRVGKYEIGRTIGEGSFAKVKFAKIETGEFFAIKVLDRDQVL
RHKMVEQIKREISTMKLIKHPNVVKMIEVMASKTKIYIVLEFVDGGELFD
KIARSGKLKEDEARRYFHQLINAVDYCHSRGVFHRDLKPENLLLDRSGAL
KISDFGLSALSQQVREDGLLHTACGTPNYVAPEVLNDKGYDGTASDVWSC
GVILFVLMAGYLPFDEPSLMSLYRKICKAEFSCPSWFSPGAKKLIKRILD
PNPHTRITISEILEDEWFKKGYKPPQFDKEEDVNLDDVDAVFNDSKEYLV
TERKEKPVSMNAFELISRSQSFNLENLFEKQTGLVKRETRFTSQRPANEI
MSKIEETAKPLGFNVRKGNYKMKLQGDKSGRKGQLSVATEVFEVAPSVHM
VEVRKTGGDTLEFHKFYKTFSSGLKDVVWQTEENDEKAVKKSR
13>NISIScaf80G0007.1 [protein]
MFNKKLGVGATATANTTTTTTEEDPNKQGMRLGKYQLGRTLGEGNFGKVK
YAKKLDSGLPFAVKILEKTKIFDLKITDQVLASKTRIYMVLEYVTGGELF
DRIDDGLLHTTCGSPNYVAPEVLSNRGYNGATSDIWSSGVILYVILTGHL
PFDDRNLAVLYQKIFKGDAKIPKWLSPGAQNILRRILDPNPVTRLTMSQI
KSDEWFKQGYTPADPTEEEEEDIIIDDEAFSIHELHSDGEKTPDSPGTIN
AFQLIGMSSCLDLSGFFEKEDVSERKIRFTSNLSMKDLLARIDSIVTEMG
FRVQKKNGRLKATQDHKGQSSLGSLSVAAEVFEISPSLYVVELRKSYGDS

AV
14>NISIScaf97G0002.1   [protein]
MFNKKLGVGATATANTTTTTTEEDPNKQGMRLGKYQLGRTLGEGNFGKVK
YAKKLDSGLPFAVKILEKTKIFDLKITDQIKREIGTLKLLKHPNVVRLHE
VLASKTRIYMVLEYVTGGELFDRIDDGLLHTTCGSPNYVAPEVLSNRGYN
GATSDIWSSGVILYVILTGHLPFDDRNLAVLYQKIFKGDAKIPKWLSPGA
QNILRRILDPNPVTRLTMSQIKSDEWFKQGYTPADPTEEEEEDIIIDDEA
FSIHELHSDGEKTPDSPGTINAFQLIGMSSCLDLSGFFEKEDVSERKIRF
TSNLSMKDLLARIDSIVTEMGFRVQKKNGRLKATQDHKGQSSLGSLSVAA
EVFEISPSLYVVELRKSYGDSAVYRQLCKRISSDLGVPSSQGLVATQV
