# Supplementary figures and images for: Genome-Wide Investigation and Expression Analysis of the Nitraria sibirica Pall. CIPK Gene Family
Source: Int J Mol Sci. 2022 Sep 30;23(19):11599. doi: 10.3390/ijms231911599 (PMC9569540; doi:10.3390/ijms231911599)

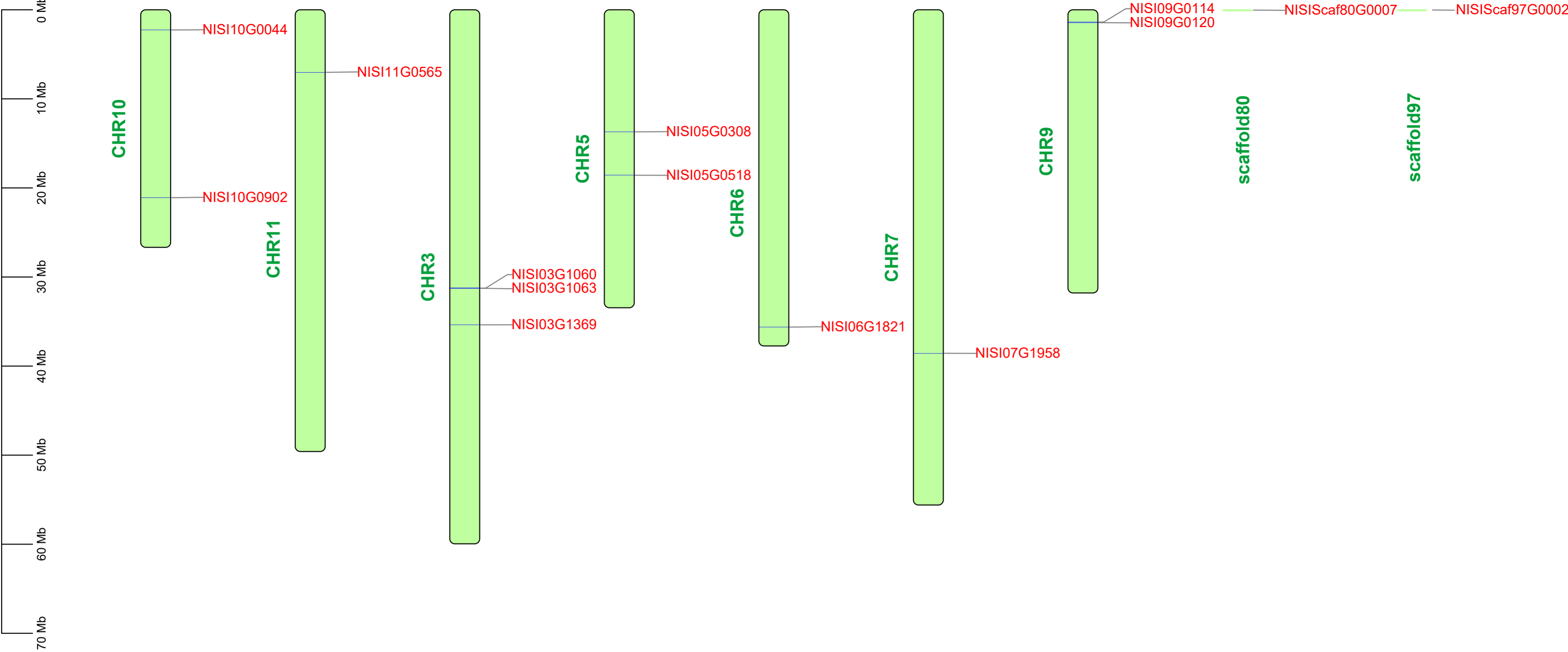

Supplement: Supplementary file 1 [file ijms-23-11599-s001.zip › Figure S1 Chromosome localization of NsCIPKs.pdf]

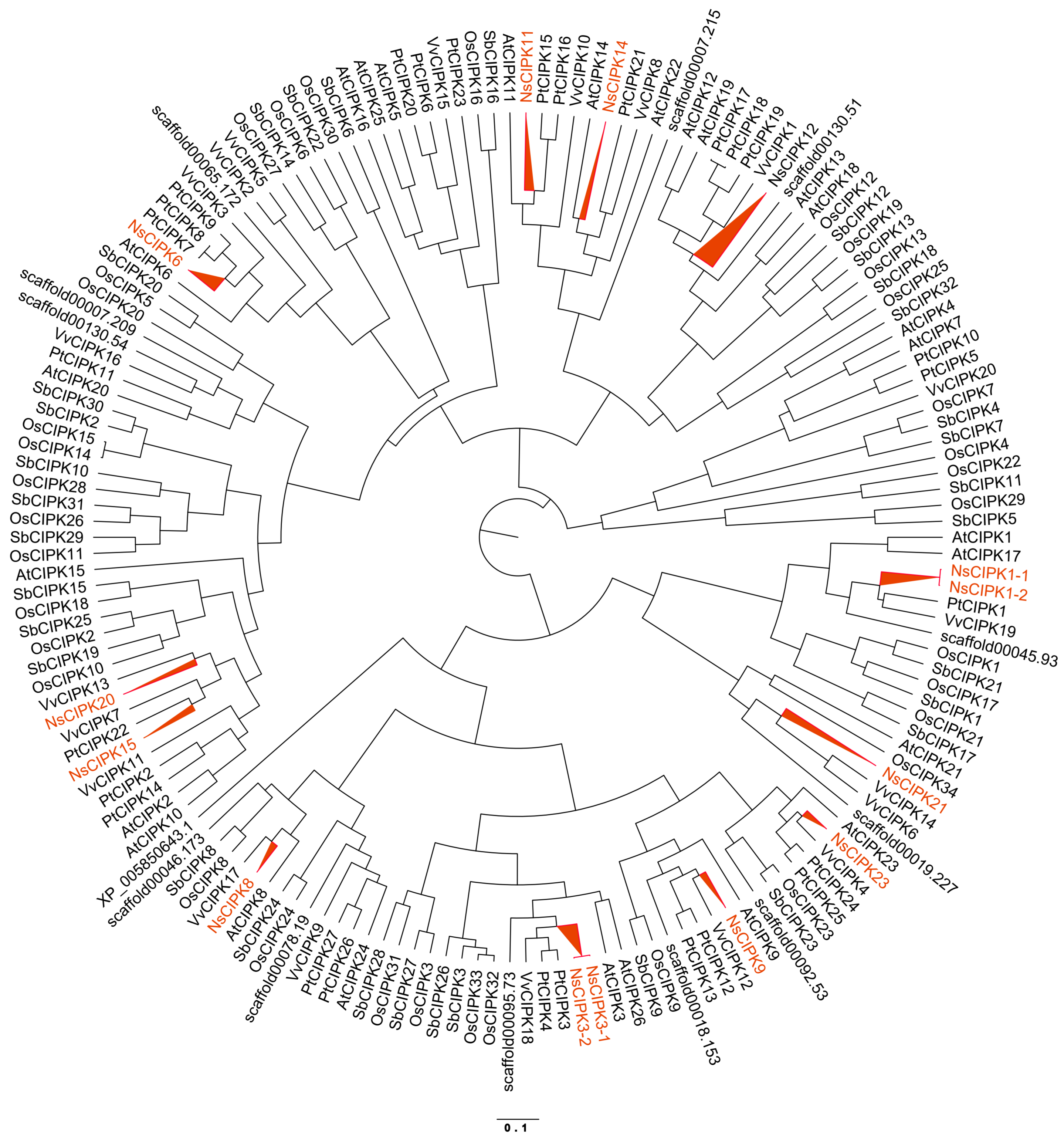

Supplement: Supplementary file 1 [file ijms-23-11599-s001.zip › Figure S3 Prediction Phylogenetic relationships between CIPKs from seven species.pdf]

Motif 1

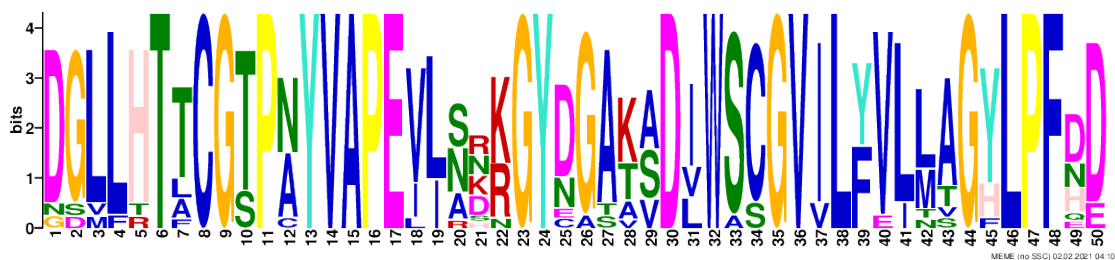

Motif 2

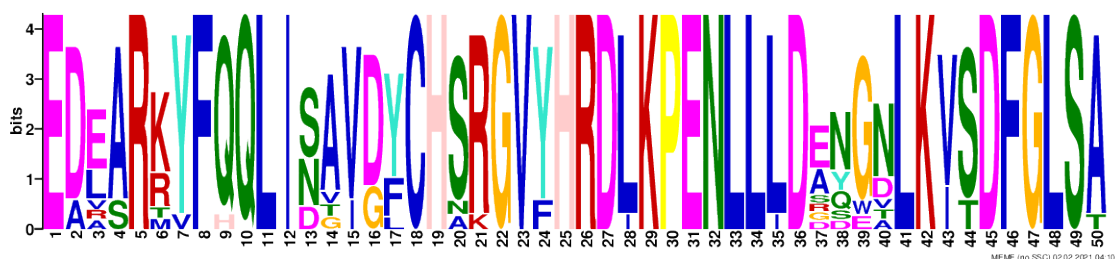

Motif 3

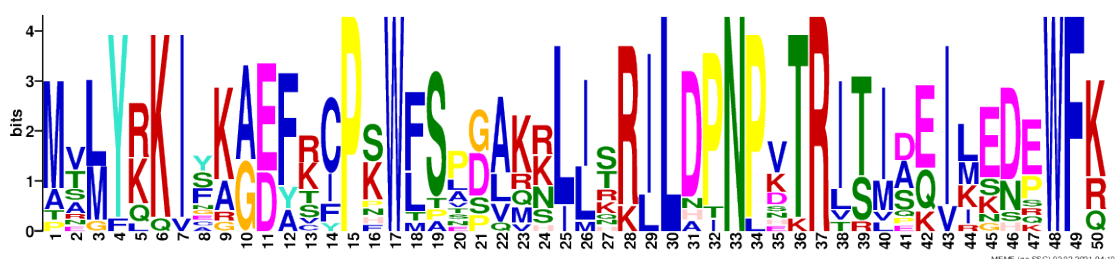

Motif 4

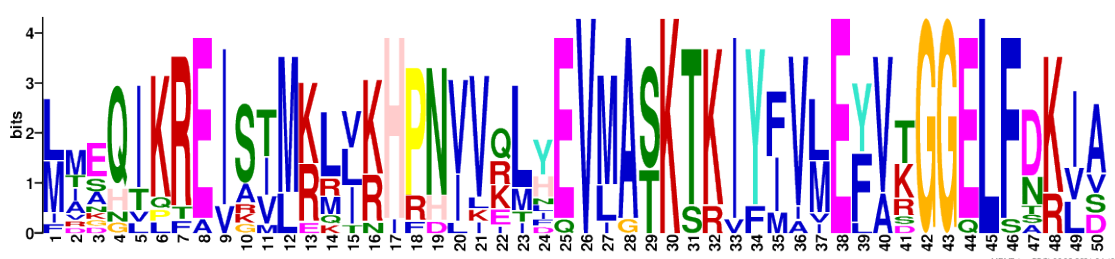

Motif 5

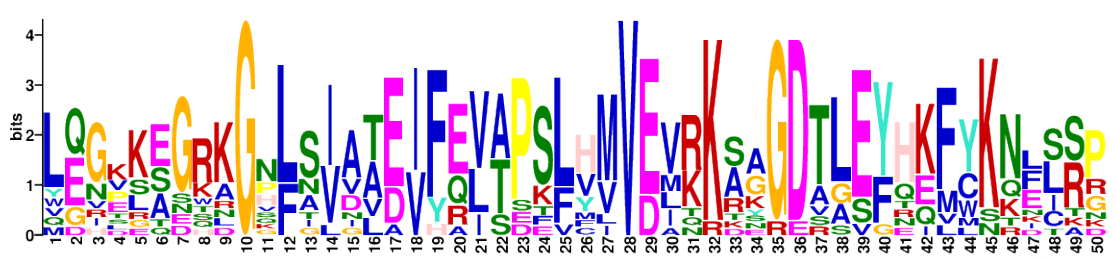

Motif 6

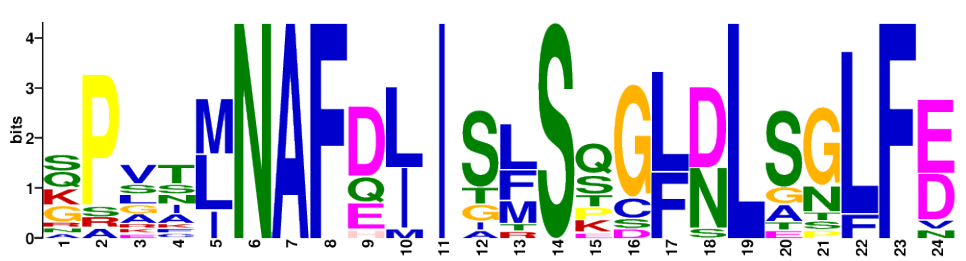

Motif 7

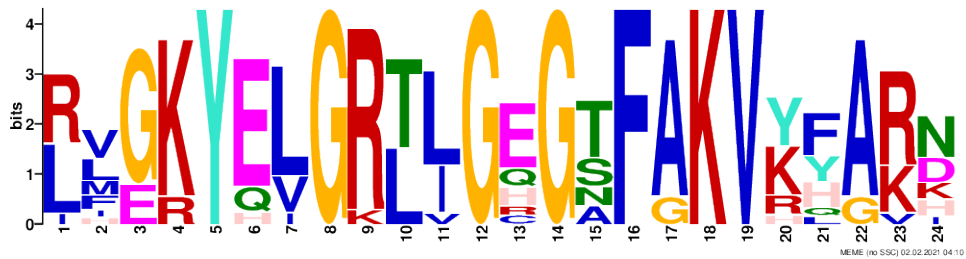

Motif 8

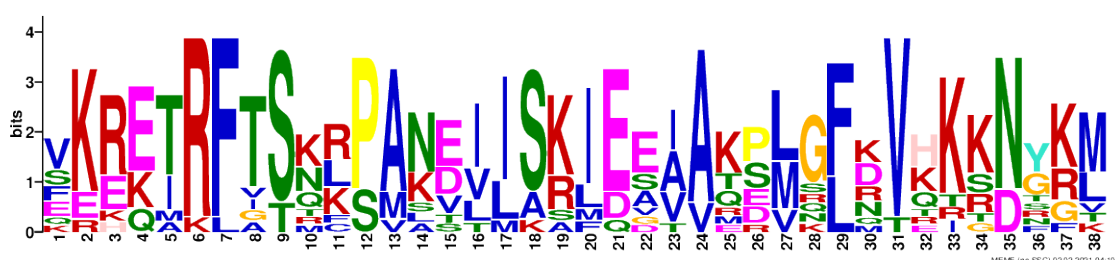

Motif 9

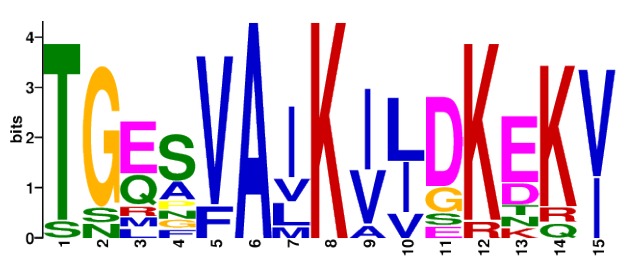

Motif 10

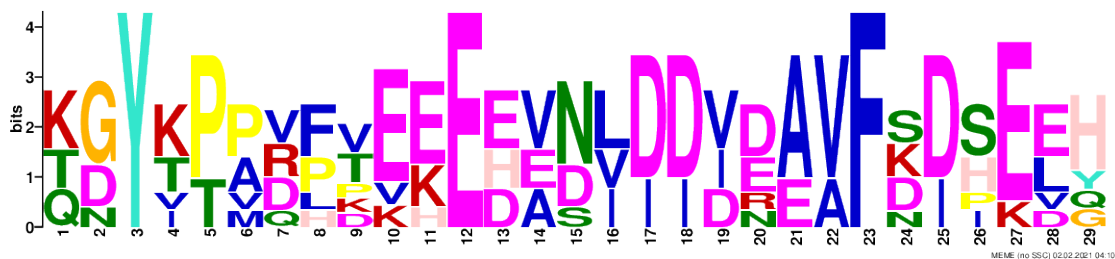

Motif 11

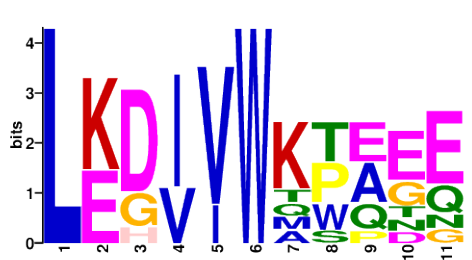

Supplement: Supplementary file 1 [file ijms-23-11599-s001.zip › Figure S5 Motif sequence distribution of NsCIPK gene family.pdf]
